# Deep Proteomics Network and Machine Learning Analysis of Human Cerebrospinal Fluid in Japanese Encephalitis Virus Infection

**DOI:** 10.1021/acs.jproteome.2c00563

**Published:** 2023-05-23

**Authors:** Tehmina Bharucha, Bevin Gangadharan, Abhinav Kumar, Ashleigh C. Myall, Nazli Ayhan, Boris Pastorino, Anisone Chanthongthip, Manivanh Vongsouvath, Mayfong Mayxay, Onanong Sengvilaipaseuth, Ooyanong Phonemixay, Sayaphet Rattanavong, Darragh P. O’Brien, Iolanda Vendrell, Roman Fischer, Benedikt Kessler, Lance Turtle, Xavier de Lamballerie, Audrey Dubot-Pérès, Paul N. Newton, Nicole Zitzmann, SEAe Consortium

**Affiliations:** † Department of Biochemistry, University of Oxford, OX1 3QU, Oxford, U.K.; □ Kavli Institute for Nanoscience Discovery, University of Oxford, OX1 3QU, Oxford, U.K.; ‡ Lao-Oxford-Mahosot Hospital-Wellcome Trust Research Unit (LOMWRU), Microbiology Laboratory, Mahosot Hospital, Vientiane, 0100 Lao PDR; § Department of Infectious Disease, Imperial College London, London W12 0NN, U.K.; ∥ Department of Mathematics, Imperial College London, London W12 0NN, U.K.; ⊥ Unité Des Virus Emergents UVE, Aix Marseille Univ, IRD190, INSERM 1207, IHU Méditerranée Infection, Marseille 13005, France; # Institute of Research and Education Development (IRED), University of Health Sciences, Ministry of Health, Vientiane 43130, Lao PDR; ∇ Centre for Tropical Medicine & Global Health, Nuffield Department of Medicine, University of Oxford, Oxford OX3 7LG, U.K.; ○ Target Discovery Institute, Centre for Medicines Discovery, Nuffield Department of Medicine, University of Oxford, Oxford OX3 7FZ, U.K.; ◆ Chinese Academy of Medical Sciences Oxford Institute, Nuffield Department of Medicine, University of Oxford, Oxford OX3 7BN, U.K.; ¶ Institute of Infection, Veterinary and Ecological Sciences, Faculty of Health and Life Sciences, University of Liverpool, Liverpool L69 7BE, U.K.; & Tropical and Infectious Disease Unit, Liverpool University Hospitals NHS Foundation Trust (Member of Liverpool Health Partners), Liverpool L69 7BE, U.K.; ■ Biology of Infection Unit, Institut Pasteur, 75015 Paris France

**Keywords:** central nervous system infection, neurological infection, encephalitis, flavivirus, Japanese encephalitis virus, diagnosis, clinical proteomics, mass spectrometry, tandem mass tagging, data-independent acquisition, network analysis, machine learning analysis, predictive modeling., Lao PDR

## Abstract

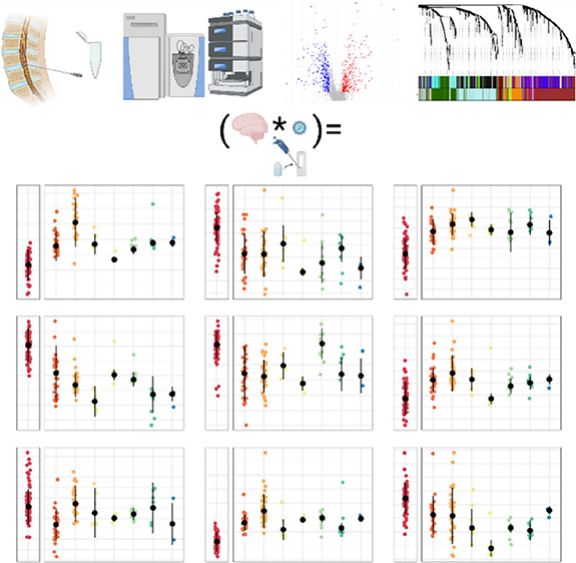

Japanese encephalitis virus is a leading cause of neurological infection in the Asia-Pacific region with no means of detection in more remote areas. We aimed to test the hypothesis of a Japanese encephalitis (JE) protein signature in human cerebrospinal fluid (CSF) that could be harnessed in a rapid diagnostic test (RDT), contribute to understanding the host response and predict outcome during infection. Liquid chromatography and tandem mass spectrometry (LC–MS/MS), using extensive offline fractionation and tandem mass tag labeling (TMT), enabled comparison of the deep CSF proteome in JE vs other confirmed neurological infections (non-JE). Verification was performed using data-independent acquisition (DIA) LC–MS/MS. 5,070 proteins were identified, including 4,805 human proteins and 265 pathogen proteins. Feature selection and predictive modeling using TMT analysis of 147 patient samples enabled the development of a nine-protein JE diagnostic signature. This was tested using DIA analysis of an independent group of 16 patient samples, demonstrating 82% accuracy. Ultimately, validation in a larger group of patients and different locations could help refine the list to 2–3 proteins for an RDT. The mass spectrometry proteomics data have been deposited to the ProteomeXchange Consortium via the PRIDE partner repository with the dataset identifier PXD034789 and 10.6019/PXD034789.

## Introduction

Japanese encephalitis virus (JEV) is a mosquito-borne flavivirus and a leading cause of neurological infection as Japanese encephalitis (JE) in Asia and the Pacific. It is of considerable public health importance, with recent estimates based on sparse data suggesting 1.5 billion people at risk with 42,000 cases per year.^1,2^ It is an emerging disease, with recent evidence of JEV in multiple territories in Australia.^3^ Patients may experience devastating socioeconomic consequences; JE predominantly affects children in poor rural areas with a 20–30% case fatality rate and 30–50% of survivors suffer long-term disability.^4^ Although no specific treatment is available, several vaccines are available and recommended by the World Health Organization (WHO).^5,6^ Although recent efforts have strengthened JEV vaccination programs, still only 15 of 24 endemic countries include JEV vaccine in routine immunization policies, and even then, it is not uniformly nationwide, with vaccine coverage in targeted areas reported to be as low as 39%.^7^ JEV is a zoonosis, and sustained vaccine coverage is essential to control disease.

A fundamental limitation in the control of JE is the poor accuracy of existing diagnostic tests, requirement for lumbar puncture and laboratory capacity for diagnosis.^8^ Surveillance data suggest that only 11 of 24 countries meet the minimum surveillance standards, equivalent to diagnostic testing in a sentinel site.^7^ This is a threat to vaccine implementation, as accessible and accurate diagnostics are essential to understand epidemiology and effectiveness of vaccination, identify associated research knowledge gaps, and facilitate public engagement. This also has implications for appropriate risk-assessment for travelers. Aside from JEV control, diagnosis is crucial for patients, families, and health-workers, to be able to institute appropriate supportive and rehabilitation care, stop unnecessary antibiotics, or if the test is negative to prompt further investigation.

The gold-standard JEV test is a neutralization assay. However, this requires paired acute and convalescent sera, is laborious, time-consuming, requires specialist skills, high-level isolation facilities for viral cell culture, and may not define the infecting virus in secondary flavivirus infections.^8^ The WHO recommended diagnostic test is anti-JEV IgM antibody capture ELISA (MAC-ELISA) of cerebrospinal fluid (CSF). There are limited data from field studies comparing CSF MAC-ELISA with neutralization assays. The manufacturer of the only available commercial kit for clinical diagnosis (InBios) quotes a sensitivity of >90% for well-characterized CSF samples, but sensitivity in the field is as low as 53%.^9^ There are also increasingly recognized problems with specificity related to prior vaccination and cross-reactivity with other flaviviruses.^10,11^ Reported specificity is >90%; however, a study by our group demonstrated that 13% of patients with anti-JEV IgM detected in CSF by MAC-ELISA had another pathogen detected that may have explained the presentation.^10^

Detection of JEV RNA would be highly specific, but the period of viraemia is brief and hard to capture clinically, often occurring before the onset of neurological symptoms and signs. RT-qPCR remains insensitive irrespective of the analytical sensitivity or gene targets.^12^ For this reason, the application of metagenomics is not likely to significantly improve JEV RNA detection.

Non-targeted, discovery-based liquid chromatography–tandem mass spectrometry (LC–MS/MS) proteomics represents an underused technology for improving diagnosis of JE from the analysis of clinical samples.^13,14^ Such an approach is based on the hypothesis that there is a protein signature in the CSF specific for JE and that diagnostic protein biomarkers could be harnessed in an antibody-based point-of-care test. Furthermore, deep proteomics exploration provides insights into disease processes and potential therapeutic targets. Network science and machine learning are two complementary disciplines enabling insights into complex high-dimensional data.^15,16^ Networks, composed of nodes and links, are naturally attuned to problems where features have a relational structure^17^ and have a track record of success in understanding networks of biological interactions.^18^ Weighted correlation network analysis (WGCNA) was developed for the analysis of transcriptomics datasets but is increasingly used in proteomics research, enabling assignment of lengthy lists of proteins into modules with biological insights.^19−21^ On the other hand, machine learning can uncover signals in data related to outcome variables and identify predictive markers of disease, a vital exploratory process for constructing diagnostics.^22^ Used in conjunction, network science and machine learning provide novel characterization of disease states and can identify robust predictive markers of disease.^23^

Herein, we aimed to test the hypothesis that there is a diagnostic protein signature of JE by performing LC–MS/MS in patient samples recruited as part of the Laos CNS study, incorporating differential expression, network, and machine learning analysis. A subsidiary aim was to utilize the data in the same workflow to evaluate proteins associated with outcome of JE. We first performed a pilot feasibility tandem mass tag labeling (TMT) LC–MS/MS study (*n* = 15) and then a larger verification TMT LC–MS/MS study (*n* = 148) including a sample size based on a power calculation. These data were combined in the final analysis (*n* = 163). The results were verified by data-independent acquisition (DIA) LC–MS/MS in 16 (10%) of the samples. Weighted correlation network analysis (WGCNA) was used to explore the data. For the purposes of feature selection and training a machine learning model for classifying JE vs non-JE patients, the TMT LC−MS/MS data was used excluding the patients analyzed by DIA LC−MS/MS (*n* = 147).. The model was tested using the DIA LC–MS/MS data (*n* = 16), providing an independent group of patient samples and alternative methodological analysis.

## Experimental Section

### Patient Samples

**Figure 1:**
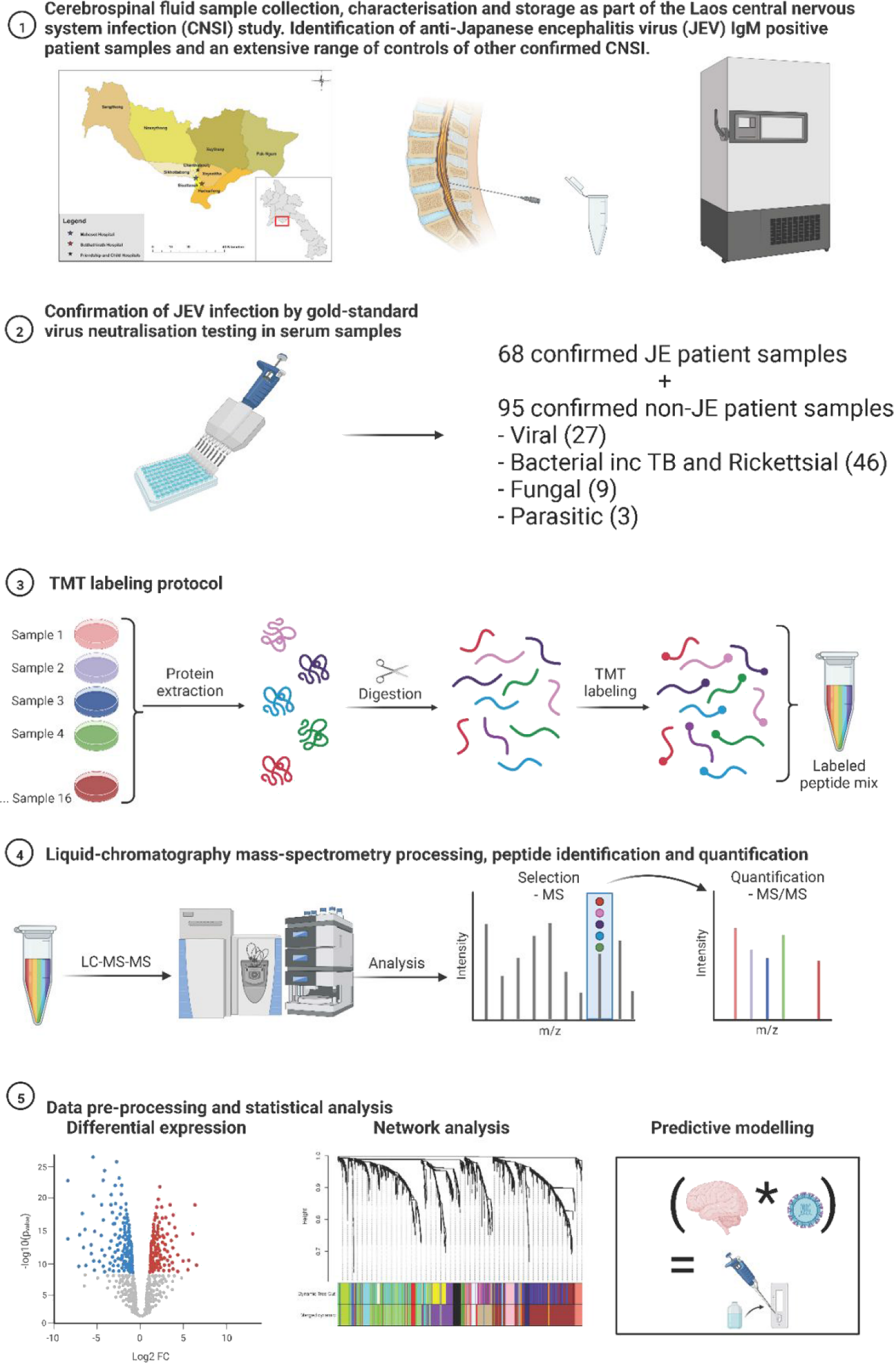
Schematic representation of the study methods. Patient cerebrospinal fluid (CSF) samples were collected during the Laos central nervous system infection study and underwent extensive diagnosis testing. Patients positive by anti-JEV IgM were confirmed by the gold-standard virus seroneutralization. The CSF of 163 patients with confirmed JE (*n* = 68) and non-JE (*n* = 95) neurological infections was analyzed by LC–MS/MS, all were tested by tandem mass tagging (TMT) and 16 were also tested by DIA LC-MS/MS. Data were analyzed by conventional univariate analysis, network analysis, and machine learning to identify diagnostic protein biomarkers of JE. The figure was created with BioRender.com.

A prospective study of central nervous system (CNS) infection has been conducted at Mahosot Hospital, Vientiane, Laos, since 2003. Methods and results from 2003 to 2011 have been described.^24^ Patients from 2014 to 2017 were included in the Southeast Asia Encephalitis Project.^25^ Inpatients of all ages were recruited for whom diagnostic lumbar puncture was indicated for suspicion of CNS infection because of altered consciousness or neurologic findings and for whom lumbar puncture was not contraindicated. There was no formal definition for CNS infection; patient recruitment was at the discretion of the responsible physician, reflecting local clinical practice. The laboratory also received samples from patients from other hospitals in Vientiane: Friendship, Children’s, and Setthathirat Hospitals. Written informed consent was obtained from patients or responsible guardians. Ethical clearance was granted by the Ethical Review Committee of the former Faculty of Medical Sciences, National University of Laos and the Oxford University Tropical Ethics Research Committee. The confirmed etiology was determined by the results of a panel of diagnostic tests which included tests for the direct detection of pathogens in CSF or blood, specific IgM in CSF, seroconversion, or a 4-fold rise in antibody titer between admission and follow-up serum samples.^24^ Pathogen detection was confirmed after critical analysis of test results to rule out possible contamination. JEV infection was confirmed, as recommended by the World Health Organization, by detection of anti-JEV IgM by ELISA in CSF or seroconversion in paired serum samples. All anti-JEV IgM positive samples were subsequently confirmed by the gold-standard virus neutralization assay see cited reference (26). Power analysis was performed to estimate the sample size that would be required using different values. A schematic representation of the study design and methods is illustrated in Figure 1.

### LC–MS Sample Preparation

CSF samples were diluted 1:5 in 9 M urea and vortexed intermittently at room temperature for 30 min, to solubilize and denature proteins, inactivating any pathogens and rendering the sample acellular. Protein concentration was assessed with a Nanodrop assay ND-1000 spectrophotometer (Thermo Scientific) by measuring the absorbance at 280 nm and normalized by aliquoting different volumes of each sample dependent on the protein concentration, and then the total volume equalized with 7.5 M urea. An equal volume of 100 mM dithiothreitol (DTT) in 50 mM ammonium bicarbonate (AmBic) was added as a reducing agent, and the samples were vortexed and incubated at 56 °C for 45 min. An equal volume of 100 mM iodoacetamide (IAA) in 50 mM AmBic was added as an alkylating agent, vortexed, and incubated at room temperature for 1 h in the dark. 50 mM AmBic was added to each sample to reduce the urea concentration to below 1 M. Digestion was performed with trypsin in a ratio of 1:20 m:m protein:trypsin (Promega, P/N V5072 for the pilot study; V5117 for the larger study); first 75% of the total amount of trypsin added and incubated at 37 °C for 18 h overnight and then the remaining 25% added and incubated at 37.5 °C for 6 h. The samples were frozen at −20 °C to quench the trypsin digestion reaction. A pooled aliquot of each sample was analyzed by label-free LC–MS to verify protein digestion.

Reverse-phase (RP) C18 solid-phase extraction (SPE) was used to desalt the digested proteins, as per the manufacturer’s instructions (Waters P/N WAT023590 for the pilot study; Thermo Scientific P/N 60109-001 for the larger study). The total eluate was dried completely using a vacuum concentrator (Savant SpeedVac or Eppendorf concentrator) and for the samples to be labeled by tandem mass tag (TMT), resuspended in 100 mM triethylammonium bicarbonate (TEAB). The samples were vortexed, centrifuged, and sonicated for 3 min, and then this was repeated. The Pierce Quantitative Colorimetric Peptide Assay (Thermo Scientific, UK) was performed as per the manufacturer’s instructions. The samples were normalized for peptide concentration with TEAB to make up a final volume of 100 μL required for TMT labeling. TMT labeling was performed as per the manufacturer’s instructions, in two batches of TMT 11-plex (Thermo Scientific, P/N A37724) for the pilot study and 10 batches of 16-plex (Thermo Scientific, P/N A44520) for the larger study. For the larger study, in order to examine technical variability and adjust for batch effects, each batch contained one reference pool and the batches 9 and 10 had two replicate samples. A pooled sample was analyzed by LC–MS to verify labeling efficiency.

### Offline High-pH Reverse-Phase Fractionation

For the pilot study, offline high-pH reverse-phase fractionation was performed using a Hypersil Gold column (Thermo Scientific, P/N 25002-202130). The mobile phase A was water adjusted with ammonium hydroxide to pH 10 and B was 10 mM ammonium bicarbonate in 80% acetonitrile (ACN) adjusted with ammonium hydroxide to pH 10 and a flow rate of 300 μL/min. The samples were separated into 91 fractions with each fraction collected every 60 s from the start of the run and using the gradient shown in Supplementary Data (S1 Data). For the larger study, offline high-pH reverse-phase fractionation was performed using an Xbridge BEH C18 column (Waters P/N 186006710). The mobile phase A was water adjusted to pH 10 with ammonium hydroxide and B was 90% ACN adjusted to pH 10 with ammonium hydroxide, at a flow rate of 200 μL/min. Fractions were collected every 60 s from the start of the run (100 fractions) and then concatenated into 44 fractions using the gradient shown in Supplementary Data (S1 Data). The samples analyzed by DIA LC–MS/MS were not processed by offline fractionation.

### Liquid Chromatography-Mass Spectrometry

Online peptide desalting was performed with a Dionex Ultimate 3000 nano UHPLC (Thermo Scientific) using 100% of loading mobile phase A = 0.05% TFA in water at a flow rate of 10 μL/min for 4.6 min. The online desalting column (trap column) used was a C18 column (Thermo Scientific P/N 160454). At 4.6 min, the flow from the nanopump was diverted to the trap column in a backward flush direction. For online low-pH reverse-phase fractionation, the trapped peptides were eluted from the column over the gradient time specified in Supplementary Data (S1 Data). For the pilot study, Accucore C18 columns (Thermo Scientific P/N 16126-507569) were used with a nanosource, at a flow rate of 250 nL/min. For the larger study, EASY-Spray PepMap C18 columns (Thermo Scientific P/N ES903) were used with an EASY-Spray source and a flow rate of 300 nL/min. Mobile phase A was 0.1% FA and B was 0.1% FA in 80% ACN. MS was performed with a Q Exactive benchtop hybrid quadrupole-Orbitrap MS (Thermo Scientific); the settings are described in detail in Supplementary Data (S1 Data). For the CSF samples processed by DIA LC–MS/MS, samples were analyzed using a Dionex Ultimate 3000 nano UPLC (Thermo Scientific) coupled to an Orbitrap Fusion Lumos mass spectrometer (Thermo Scientific). Briefly, peptides were trapped on a PepMap C18 trap columns (Thermo) and separated on an EasySpray column (50 cm, P/N ES803, Thermo) over a 60-min linear gradient from 2% buffer B to 35% buffer B (A: 5% DMSO, 0.1% formic acid in water. B: 5% DMSO, 0.1% formic acid in acetonitrile) at a flow rate of 250 nL/min. The instrument was operated in data-independent mode as previously described.^27^

### Data Processing and Statistical Analysis

The sample size was estimated using a power calculation based on a *t* test and multiple testing correction, with data from the pilot study and the R package “FDRsampsize”.^28^

TMT LC–MS/MS analysis protein identification, quantification, missing value imputation and batch correction: Thermo raw files were imported into Proteome Discoverer v2.5 (Thermo Scientific, UK) for peptide identification using the SEQUEST algorithm^29^ searching against the SwissProt *Homo sapiens* and pathogen databases according to the included samples with precursor mass tolerance 10 ppm and fragment mass tolerance 0.02 Da. Carbamidomethylation of cysteine, TMT at N-termini and lysine were set as fixed modifications, and oxidation of methionine was set as a variable modification. False discovery rate (FDR) estimation was performed using the Percolator algorithm.^30^ The criteria for protein identification included FDR < 1%, ≥2 peptides per protein, ≥1 unique peptide per protein, ≤2 missed cleavages and ≥6 and ≤144 peptide length (amino acids), coisolation threshold <50%, average S/N threshold >10, and at least two channels with quantification data. Protein quantification was performed in R v 4.1.2 with the package MSstatsTMT.^31^ Proteins with >50% missing data were removed, and the data were imputed with the package DreamAI.^32^ To incorporate peptide count per protein, jitter was added proportional to 1/median peptide count for each protein. The pilot and larger study data were merged and normalized with the package RobNorm,^33^ and then batch correction was performed with the function ComBat^34^ in the package sva without modifiers as covariates.^35^ The protein list was filtered to remove potential contaminant proteins from the skin or red blood cells, see Supplementary Data S5_contaminants for the list of proteins removed. The effectiveness of batch correction was performed by visualizing the processed data using principal component analysis and hierarchical clustering in MetaboAnalyst v5.0.^36^

#### TMT LC–MS/MS Data Differential Protein Expression

Differential expression between the protein abundance in the JE vs non-JE patient samples was performed using a *t* test and Benjamini–Hochberg correction for multiple testing.

#### TMT LC–MS/MS Data Protein Set Enrichment Analysis

Functional analysis of human proteins identified in JE vs non-JE patient samples was performed using the WebGestalt online tool^37^ using gene set enrichment analysis (GSEA) and gene ontology.

#### TMT LC–MS/MS Data Network Analysis

WGCNA was performed using the package WGCNA: constructing a signed weighted coexpression network with a soft power threshold of 12 to produce a power distribution, that is, scale-free topology; applying hierarchical clustering to detect modules of highly interconnected proteins with a minimum module size of five, deepSplit 4 and merge threshold 0.3; classifying intramodular hub proteins as the five proteins with the highest module membership for each module; and then correlating the modules with patient sample data.^38^

#### Data-Independent Acquisition (DIA) Data Processing

For robustness, final verification was performed on 10% of the samples independently processed via a separate mass spectrometry pipeline using label-free DIA LC–MS/MS. DIA data were analyzed using DIA-NN software (v0.8) with the library-free approach as previously described,^39^ using the default settings as recommended. Briefly, for the library-free processing, a library was created from human UniProt SwissProt database (downloaded 24/2/21 containing 20,381 sequences) using deep learning. Trypsin was selected as the enzyme (1 missed cleavage), with carboamidomethylation of C as a fixed modification, oxidation of methionine as a variable modification, and N-term M excision. Identification and quantification of raw data were performed against the in silico library applying 1% FDR at precursor level and match between runs (MBR). The DIA-NN “report.proteingroup” matrix output was further analyzed. Missing values were imputed with half the minimum value for each protein.

Feature selection and predictive modeling: This was performed using the TMT LC–MS/MS data without the samples processed by DIA (*n* = 147) with the Boruta algorithm (using the random forest classifier) using the package Boruta^40^ and with Lasso (least absolute shrinkage and selection operator) regression using the package glmnet.^16,41^ A final list of proteins based on the intersect between Boruta and Lasso was selected.^42^ Classification of JE vs non-JE was performed with selected proteins using several different machine learning models (random forest, support vector machine, logistic regression, and naïve bayes with the package caret and caretEnsemble).^43^ Models were trained using tenfold cross-validation repeated 10 times evaluated on AUC-ROC. The ensemble model was tested with the DIA LC–MS/MS data (*n* = 16). An analysis of feature importance was performed to identify proteins that best predicted the outcome (alive/died) in JE patients, however due to the small sample size this was considered an exploratory analysis. Feature selection was performed with Boruta and Lasso, and then fivefold cross-validation was performed on the entire TMT LC–MS/MS dataset using different machine learning models. Protein involvement in biological, molecular, and cellular processes was explored using gene ontology using the webserver STRING,^44^ the R package WebGestaltR 0.4.4,^45^ and tissue expression correlated with the Human Protein Atlas (HPA).^46,47^

## Results

### Patient Data

Power analysis was performed to estimate the sample size that would be required to compare differential expression of proteins in JE vs non-JE using different values: with 1,000–3,000 biomarkers to be tested, 50–150 finally verified, effect size 0.8, power 90%, FDR < 5%, the total sample size with an equal number of JE cases and non-JE controls of 122. Overall, including the pilot and larger study, 163 patients were included: 68 JE and 95 Non-JE; see Table 1, Supplementary Data S2 and S3.

**Table 1:** Summary of Included Patients’ Demographics, Clinical Presentations, and Details of Diagnosis

		JE^a^	non-JE viral infections^b^	bacterial infections^c^	Tuberculosis^d^	Rickettsial infections^e^	scrub typhus^f^	fungal infections^g^	parasitic infections^h^	total (*N* = 163)
age (years)	median	16	22	38	53	48	20	33	25	22
	IQR	10.0, 23.2	6.0, 35.0	20.0, 55.0	32.5, 55.5	32.0, 53.0	11.8, 24.0	27.0, 47.0	24.5, 42.0	12.0, 41.5
sex	female	24 (35.3%)	10 (37.0%)	9 (28.1%)	1 (14.3%)	1 (20.0%)	4 (33.3%)	0 (0.0%)	0 (0.0%)	49 (30.1%)
ethnicity	Lao loum	35 (51.5%)	25 (92.6%)	23 (71.9%)	7 (100.0%)	5 (100.0%)	11 (91.7%)	8 (88.9%)	3 (100.0%)	117 (71.8%)
	Lao sung	16 (23.5%)	1 (3.7%)	3 (9.4%)	0 (0.0%)	0 (0.0%)	0 (0.0%)	0 (0.0%)	0 (0.0%)	20 (12.3%)
	Lao theung	1 (1.5%)	0 (0.0%)	1 (3.1%)	0 (0.0%)	0 (0.0%)	0 (0.0%)	0 (0.0%)	0 (0.0%)	2 (1.2%)
	Lao unspecified	16 (23.5%)	1 (3.7%)	5 (15.6%)	0 (0.0%)	0 (0.0%)	1 (8.3%)	1 (11.1%)	0 (0.0%)	24 (14.7%)
comorbidities	N-Miss	0	0	0	0	0	1	0	0	1
	yes	3 (4.4%)	5 (18.5%)	3 (9.4%)	4 (57.1%)	1 (20.0%)	0 (0.0%)	5 (55.6%)	0 (0.0%)	21 (12.9%)
	HIV	0 (0.0%)	3 (11.1%)	0 (0.0%)	0 (0.0%)	0 (0.0%)	0 (0.0%)	5 (55.6%)	0 (0.0%)	8 (4.9%)
duration of illness (days)	median	4	3	3	6	2	5.5	5	1	4
	IQR	3.8, 5.2	1.0, 5.5	2.0, 5.2	3.0, 7.0	2.0, 4.0	2.8, 8.2	2.0, 7.0	0.5, 4.0	2.0, 6.0
seizures	yes	29 (42.6%)	9 (33.3%)	6 (18.8%)	0 (0.0%)	2 (40.0%)	3 (25.0%)	1 (11.1%)	0 (0.0%)	50 (30.7%)
antibiotics prior to LP	N-Miss	13	1	3	2	0	3	3	0	25
	yes	44 (80.0%)	15 (57.7%)	12 (41.4%)	4 (80.0%)	2 (40.0%)	8 (88.9%)	1 (16.7%)	1 (33.3%)	87 (63.0%)
GCS	N-Miss	7	0	1	0	0	1	0	0	9
	Median	11	14	12	10	10	15	15	15	12
	IQR	8.0, 13.0	9.5, 15.0	10.0, 15.0	8.5, 12.5	7.0, 14.0	14.0, 15.0	12.0, 15.0	15.0, 15.0	9.0, 15.0
blood WCC (10^6^/μL)	N-Miss	9	1	5	0	0	1	0	0	16
	Median	12.8	11.3	12.8	11.5	8.3	13	8.4	7.5	11.7
	IQR	8.4, 17.4	7.8, 12.9	9.4, 17.5	8.6, 12.1	7.0, 13.0	10.0, 13.6	4.8, 14.6	7.4, 8.9	8.1, 15.1
blood CRP (mg/L)	N-Miss	50	11	18	5	1	5	3	1	94
	Median	45	45.1	184.5	20.3	41.6	102.5	33.5	9.8	46.7
	IQR	24.7, 73.1	4.5, 93.1	106.3, 196.5	16.5, 24.0	3.9, 103.4	33.6, 131.3	10.6, 45.5	5.1, 14.6	19.4, 112.6
CSF opening pressure (cm of H_2_O)	N-Miss	11	3	0	0	0	1	0	0	15
	Median	23	20	23.5	24	15	23	25.5	27	22
	IQR	18.0, 26.0	16.2, 27.4	11.4, 32.8	18.8, 28.0	11.5, 20.0	20.0, 25.0	12.0, 38.0	22.5, 28.0	16.8, 28.2
CSF color	N-Miss	4	1	3	0	0	4	0	1	13
	clear	50 (78.1%)	18 (69.2%)	6 (20.7%)	6 (85.7%)	3 (60.0%)	7 (87.5%)	6 (66.7%)	1 (50.0%)	97 (64.7%)
	red	1 (1.6%)	3 (11.5%)	1 (3.4%)	0 (0.0%)	1 (20.0%)	0 (0.0%)	0 (0.0%)	0 (0.0%)	6 (4.0%)
	turbid	13 (20.3%)	4 (15.4%)	20 (69.0%)	1 (14.3%)	0 (0.0%)	0 (0.0%)	3 (33.3%)	1 (50.0%)	42 (28.0%)
	yellow	0 (0.0%)	1 (3.8%)	2 (6.9%)	0 (0.0%)	1 (20.0%)	1 (12.5%)	0 (0.0%)	0 (0.0%)	5 (3.3%)
CSF WCC (10^6^/L)	N-Miss	3	3	2	1	2	1	1	0	13
	median	110	147.5	655	192.5	25	60	140	500	150
	IQR	50.0, 215.0	36.2, 255.0	206.2, 1573.8	98.8, 316.2	17.5, 35.0	47.5, 132.5	32.5, 260.0	492.5, 1115.0	51.2, 340.0
CSF % neutrophils	N-Miss	3	3	3	1	2	2	1	0	15
	median	25	29	92.7	30.2	89	67	93.6	72	45.5
	IQR	9.3, 48.4	12.4, 62.8	74.0, 97.2	21.8, 76.6	69.5, 94.5	58.9, 78.0	75.2, 100.0	66.5, 85.0	15.0, 89.6
CSF % lymphocytes	N-Miss	3	3	3	1	2	2	1	0	15
	median	75	71	7.3	69.8	11	33	6.4	28	54.5
	IQR	51.6, 93.3	37.2, 87.6	2.8, 26.0	23.4, 78.2	5.5, 30.5	22.0, 41.1	0.0, 24.8	15.0, 33.5	10.4, 85.0
CSF RCC (10^6^/L)	N-Miss	2	0	3	0	0	0	0	0	5
	median	0	0	0	0	75	14.5	5	75	0
	IQR	0.0, 0.0	0.0, 27.5	0.0, 135.0	0.0, 12.5	5.0, 1005.0	0.0, 37.5	0.0, 18.0	37.5, 85.0	0.0, 19.5
CSF total protein (mg/dL)	N-Miss	6	1	1	0	1	2	0	0	11
	median	60	58.5	111	128	72.5	93.5	39	96	76.5
	IQR	38.2, 98.8	30.5, 117.5	57.5, 259.5	97.5, 270.0	60.0, 75.2	57.8, 139.5	20.0, 88.0	59.0, 108.0	38.8, 120.0
CSF glucose (mmol/L)	N-Miss	4	2	2	0	1	2	0	0	11
	median	3.7	3.3	3	2	5.1	3.4	2.6	2.9	3.4
	IQR	2.9, 4.7	1.9, 4.9	1.7, 5.3	1.4, 4.6	3.7, 5.8	3.1, 6.5	1.8, 2.9	2.6, 4.2	2.3, 4.8
duration of admission (days)	N-Miss	34	3	5	0	1	3	3	0	49
	median	13.5	8.5	11	20	9.5	5	19.5	8	11
	IQR	10.2, 17.0	7.0, 12.2	6.0, 16.0	8.0, 21.5	6.5, 12.0	5.0, 8.0	14.2, 27.8	7.5, 10.0	7.0, 16.0
outcome	N-Miss	26	3	7	0	1	2	5	0	44
	died	9 (21.4%)	2 (8.3%)	7 (28.0%)	1 (14.3%)	0 (0.0%)	1 (10.0%)	0 (0.0%)	0 (0.0%)	20 (16.8%)

a JE= Japanese encephalitis"; Miss = missing value.

b LP = lumbar puncture.

c GCS = Glasgow Coma Scale.

d WCC = white cell count.

e CRP = c-reactive protein.

f CSF = cerebrospinal fluid.

g RCD = red cell count.

h IQR = interquartile range.

JE patients were confirmed by the assays with the highest diagnostic confidence; detection of JEV RNA, or detection of anti-JEV IgM in CSF or by seroconversion and confirmed by virus neutralization tests (VNT). Non-JE patients included a range of different categories of infection that are common in the region. None of the patients had dual infections. Details of patient demographics, clinical presentations, laboratory investigations and outcome are reported in Supplementary Data S1 and S2.

### Protein Profiling in CSF Reveals Differential Expression in JE

5,070 proteins were identified, including 4,805 human proteins and 265 pathogen proteins, see Supplementary Data S4 for MSstatsTMT output for the pilot and larger studies. The pathogen proteins were bacterial or parasitic proteins. 2,244 human proteins were identified in more than half of the samples included in both the pilot and larger studies. 68 proteins deemed to be contaminants were removed from the list, see Supplementary Data S5, resulting in a filtered list of 2,176 proteins. Data processing and batch correction were effective, see Supplementary Data S7.

**Figure 2:**
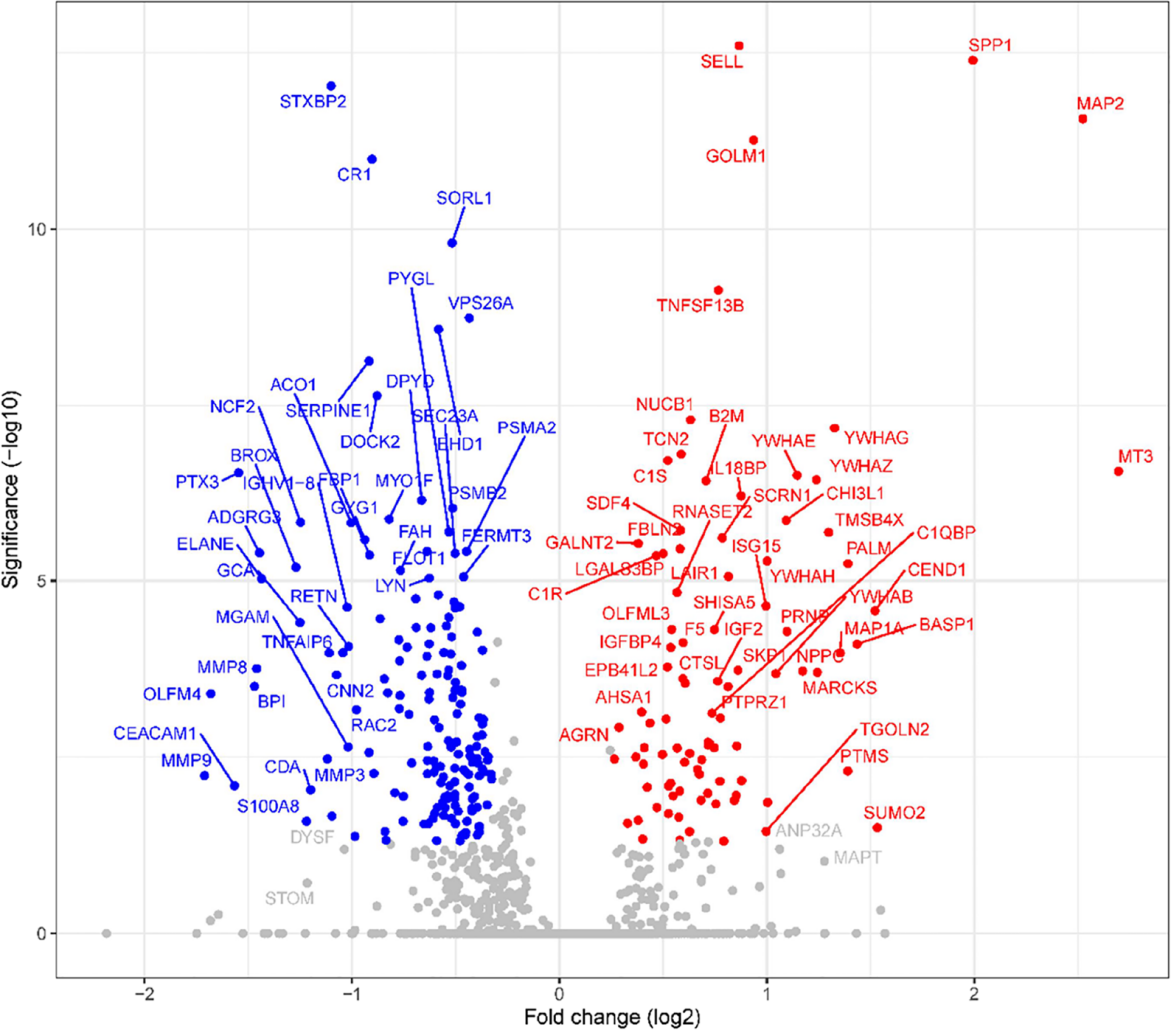
Differential CSF proteome between JE and non-JE neurological infections. Volcano plot of the identified proteins illustrating the statistical significance (*t* test *p* values with multiple testing correction) against the magnitude of change (fold change) for JE vs non-JE neurological infections. Proteins with differential expression are colored and labeled by gene names, upregulated proteins in red, and downregulated proteins in blue.

268 proteins showed differential expression (167 > 1.2-fold change, FC, and 101 < 0.8 FC) based on the performance of a *t* test and Benjamini–Hochberg multiple testing correction with *p* value <0.05, illustrated by the volcano plots in Figure 2.

### Molecular Pathways Associated with JE in CSF

**Figure 3:**
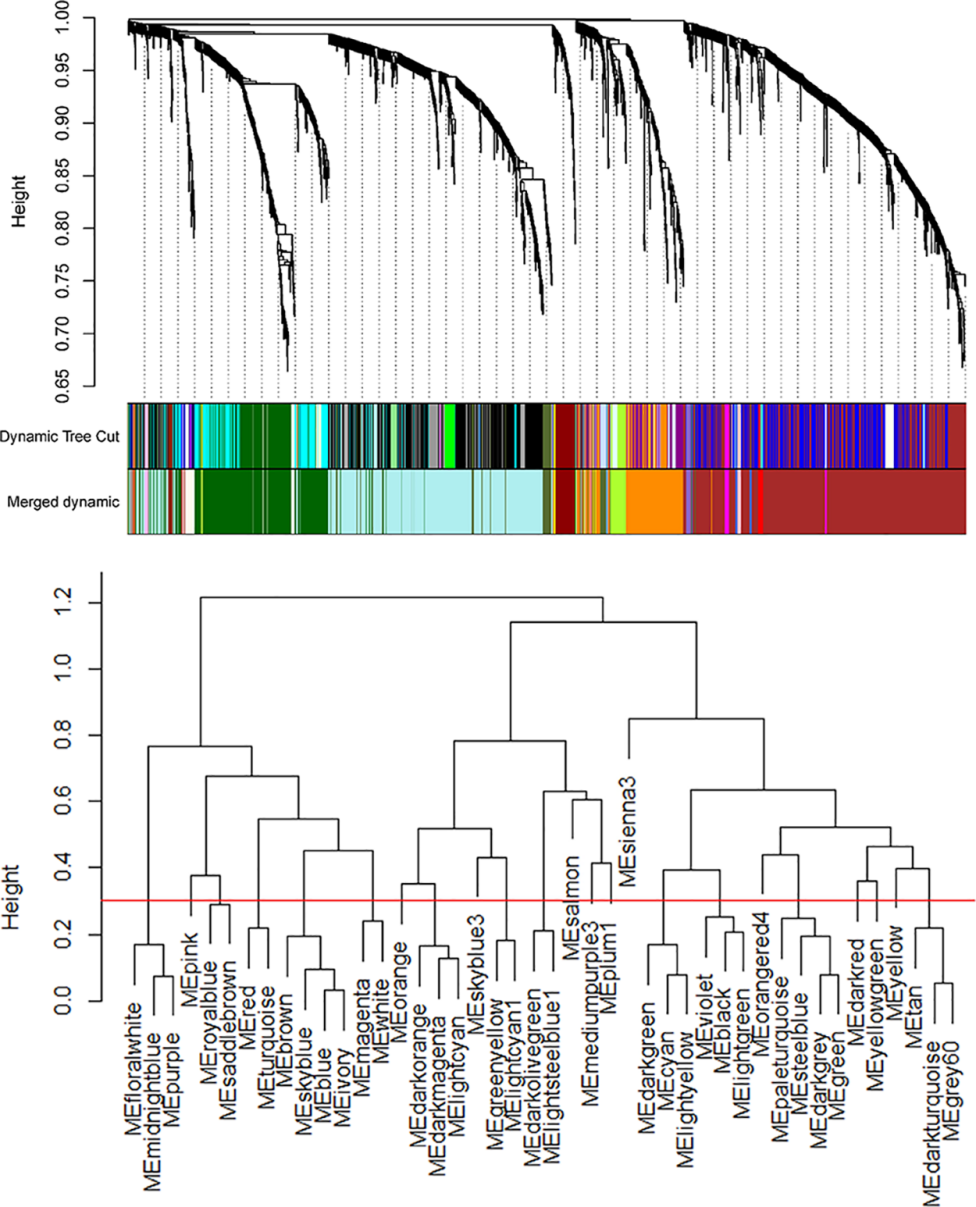
WGCNA identified 20 modules enriched in the CSF of JE patients. Cluster dendrogram (top panel) and clustering of module eigengenes (bottom panel). Expression of 2176 proteins was analyzed using the R package WGCNA, enabling unsupervised construction of correlation-based clusters, called “modules.” Modules with close correlation (threshold = 0.3) were merged, producing 20 modules. The red line in the figure indicates the threshold for merging modules together, here the threshold was 0.3.

**Figure 4:**
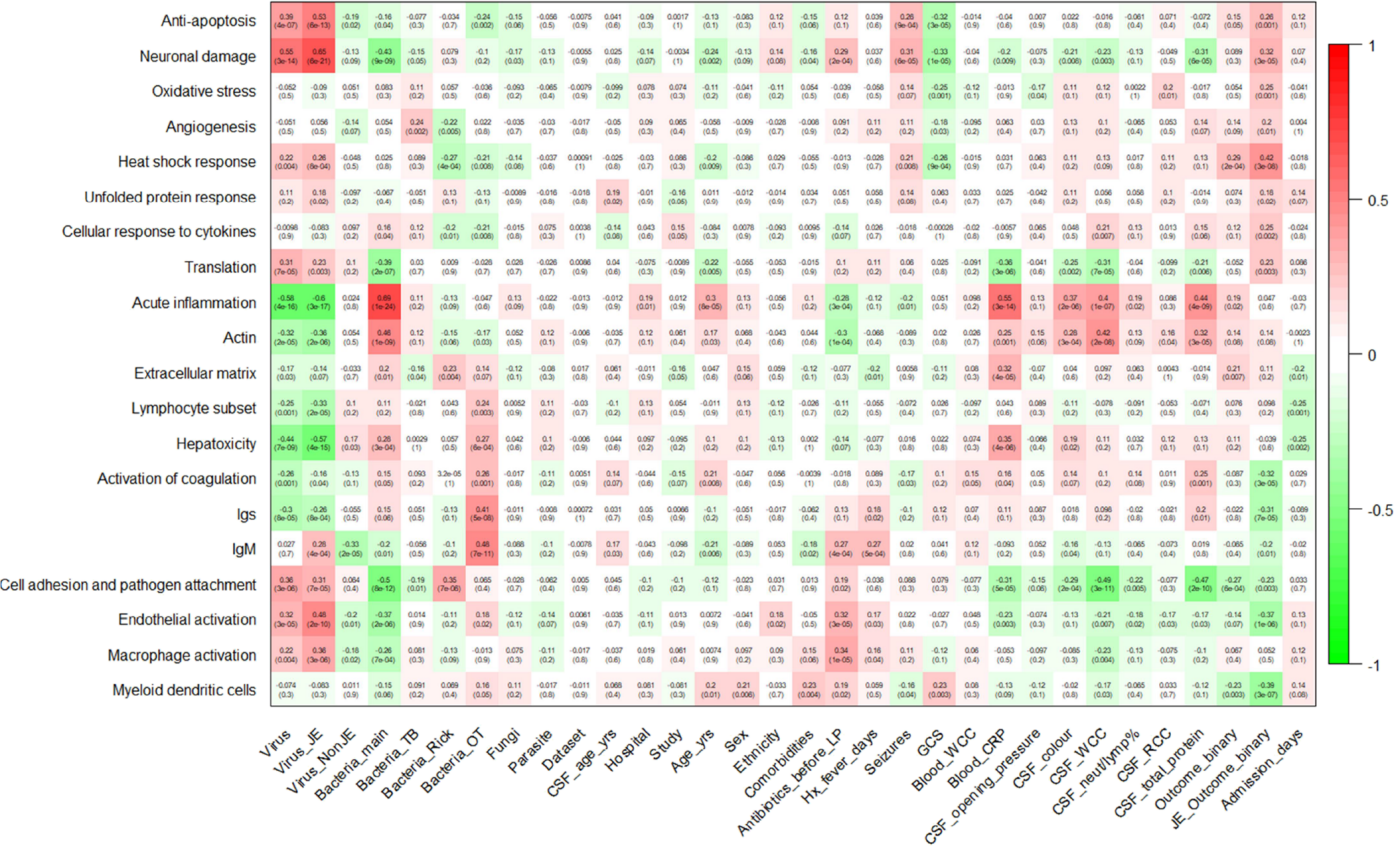
WGCNA module-trait relationships reveal enriched molecular pathways associated with patient clinical conditions. The 20 modules were allocated a descriptor (*y* axis), according to the clinical and biological associations of the proteins. The correlation with module protein expression was analyzed against patient data (*x* axis). Virus_JE = Japanese encephalitis; Virus_NonJE = non-JE viral infections; Bacteria_main = bacterial infections; Bacteria_TB = tuberculosis; Bacteria_Rick = rickettsial infections; Bacteria_OT = scrub typhus; Fungi = fungal infections; Parasite = parasitic infections; CSF = cerebrospinal fluid; LP = lumbar puncture; GCS = Glasgow Coma Score; WCC = white cell count; CRP = c-reactive protein; RCC = red cell count.

2,176 proteins from 163 patient samples were used in protein set enrichment analysis, detailed in Supplementary Data S8, and to build a weighted gene expression network, detailed in Figures 3 and 4 and Supplementary Data S9–S11. A single outlier was identified, see Supplementary Data S9, and removed. Further analysis revealed that this sample had higher overall protein abundances, in spite of peptide normalization prior to TMT labeling and downstream normalization in MSstatsTMT and RobNorm during data processing. 44 modules were identified, and then closely related modules merged into 20 modules, see the tree diagram illustrating the cluster dendrogram and the modules in Figure 3 (top and bottom panels respectively). Module-trait relationships are shown in Figure 4; suggesting that 15 modules were associated with JE (*p* value <0.05), 9 upregulated (red) and 6 downregulated (green). 10 of the modules included proteins in the top five intramodular proteins, that is, proteins with the highest modular membership, with significant differences in abundance between the JE and non-JE group.

**Table 2:** Predictive Modeling Scores with 95% Confidence Intervals

classification task	data	AUC-ROC	accuracy	sensitivity	specificity	positive predictive value	negative predictive value
JE diagnosis (JE vs non-JE)	training set^a^ (*n* = 147)	98.7 (98.0–99.4)	97.0 (95.7–98.0)	99.8 (98.9–100)	94.2 (91.8–96.1)	94.5 (92.2–96.3)	99.8 (98.8–100)
	test set^b^ (*n* = 16)	95.5 (86.6–100)	81.3 (54.4–96.0)	100 (47.8–100)	72.7 (39.0–94.0)	62.5 (24.5–91.5)	100 (63.1–100)
JE outcome (dead vs alive)	training set^c^ (*n* = 42)	88.5 (84.7–92.2)	86.3 (83.5–88.8)	42.0 (32.2–52.3)	93.7 (91.4, 95.5)	52.5 (41.0–63.8)	90.6 (88.1–92.8)

a The training set included patient samples processed by TMT LC–MS/MS; performance metrics are calculated by cross-validation.

b The test set included patient samples processed by label-free DIA LC–MS/MS.

c The training set included all the JE patients included in the TMT LC–MS/MS analysis for which outcome data was available; performance metrics are calculated by cross-validation.

### Diagnostic Protein Signature of JE in CSF

#### Feature Selection

**Figure 5:**
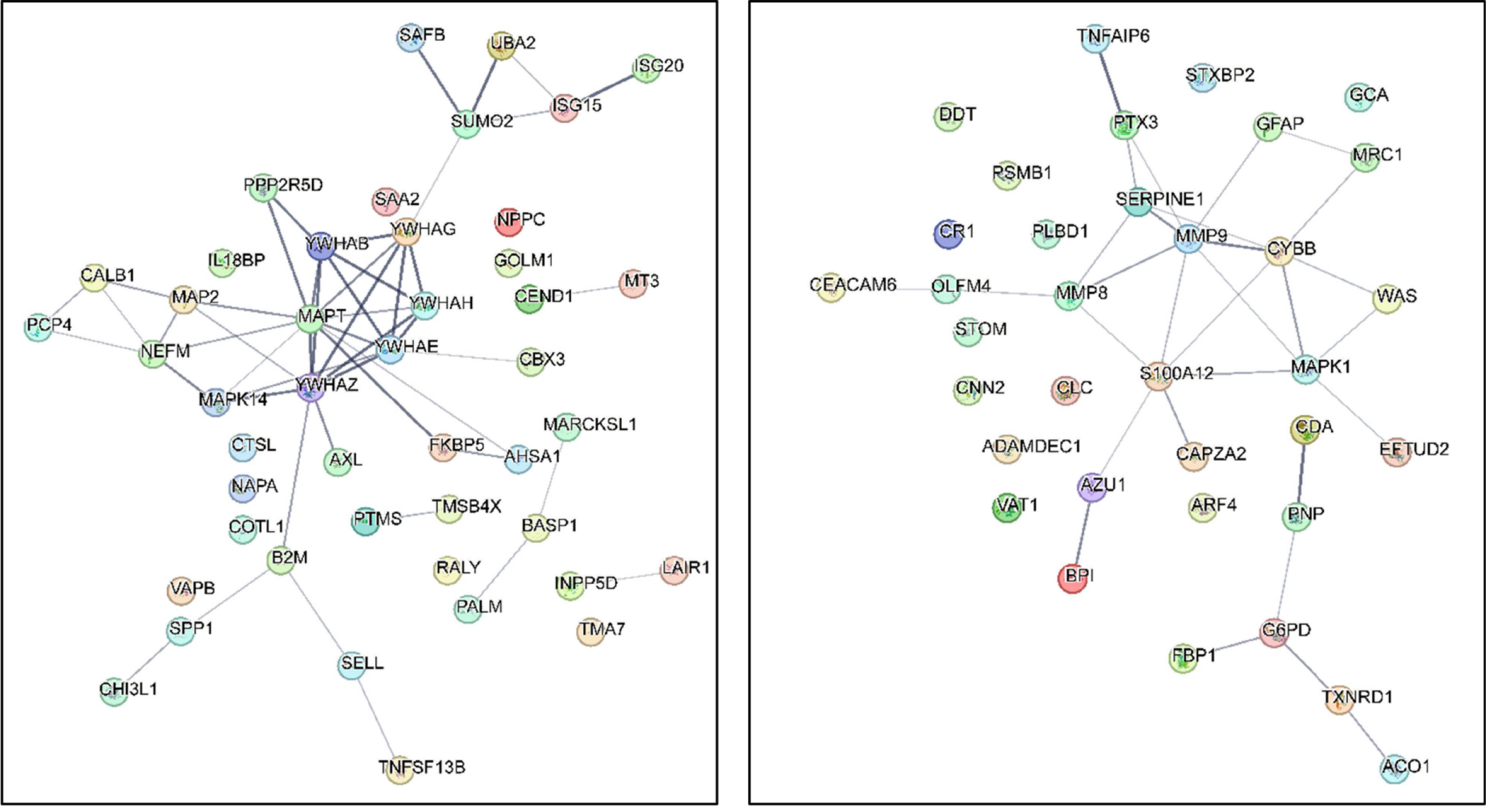
Molecular signatures enriched in JE patient CSF. STRING functional protein association network for upregulated (left) and downregulated (right) proteins. The protein clusters are derived by hierarchically clustering the full STRING network of 83 proteins identified as being important in diagnosing JE using an average linkage algorithm. The smallest clusters generated consist of five proteins and largest used in the enrichment analysis contain 200 proteins. In order to reduce redundancy, clusters with a size difference of less than five proteins toward their child cluster are removed.

1,736 proteins were present in both the TMT and DIA LC–MS/MS data. In total, 83 proteins were identified by at least one of the feature selection procedures as important in classifying JE vs non-JE; 68 proteins identified with the Boruta algorithm and 24 with Lasso, see Supplementary Data S12. The proteins were associated with 10 different WGCNA modules, all of which had been identified as associated with JE through WGCNA. Forty seven were upregulated and 36 downregulated in JE in comparison to other neurological infections. Functional enrichment analysis in STRING demonstrated interactions between the proteins, Figure 5. Twenty-two proteins were secreted proteins: Immunoglobulin lambda variable 3–9 (IGLV3–9), immunoglobulin heavy variable 3–74 (IGHV3–74), Golgi membrane protein 1 (GOLM1), Cathepsin L (CTSL), CEA cell adhesion molecule 8 (CEACAM8), phospholipase B domain containing 1 (PLBD1), Cerebellin 1 precursor (CBLN1), secreted phosphoprotein 1 (SPP1), Natriuretic peptide C (NPPC), microtubule-associated protein tau (MAPT), Chitinase 3 like 1 (CHI3L1), ISG15 ubiquitin like modifier (ISG15), Interleukin 18 binding protein (IL18BP), Beta-2-microglobulin (B2M), TNF superfamily member 13b (TNFSF13B), bactericidal permeability increasing protein (BPI), Pentraxin 3 (PTX3), matrix metallopeptidase 9 (MMP9), S100 calcium binding protein A12 (S100A12), Azurocidin 1 (AZU1), Olfactomedin 4 (OLFM4), and matrix metallopeptidase 8 (MMP8). 15 proteins were associated with increased expression in the brain: Brain abundant membrane attached signal protein 1 (BASP1), Aldolase, fructose-bisphosphate C (ALDOC), CBLN1, Metallothionein 3 (MTX3), MAP2, Tyrosine 3-monooxygenase/tryptophan 5-monooxygenase activation protein gamma (YWHAG), Tyrosine 3-monooxygenase/tryptophan 5-monooxygenase activation protein eta (YWHAH), MARCKS like 1 (MARCKSL1), Secernin 1 (SCRN1), SPP1, MAPT, CHI3L1, Paralemmin (PALM), Reticulon 1 (RTN1), Purkinje cell protein 4 (PCP4), Cytidine/uridine monophosphate kinase 2 (CMPK2), NPPC, Glial fibrillary acidic protein (GFAP), cell cycle exit, and neuronal differentiation 1 (CEND1). Thus, three proteins were secreted and showed an increased expression in the brain: SPP1, MAPT, CHIL3, NPPC, and CBLN1.

JEV has a predilection for the thalamus and substantia nigra of the basal ganglia.^26^ One of the proteins were “group enriched” in the thalamus, MMP9, from the HPA database. Four proteins were associated with the GO term “substantia nigra development”, associated with BASP1, Glucose-6-phosphate dehydrogenase (G6PD), YWHAH, and 14-3-3 protein epsilon (14-3-3epsilon). The HPA database includes mRNA expression data from 13 brain regions, including the basal ganglia and thalamus; substantia nigra expression on its own is not reported (https://www.proteinatlas.org/humanproteome/brain).

**Figure 6:**
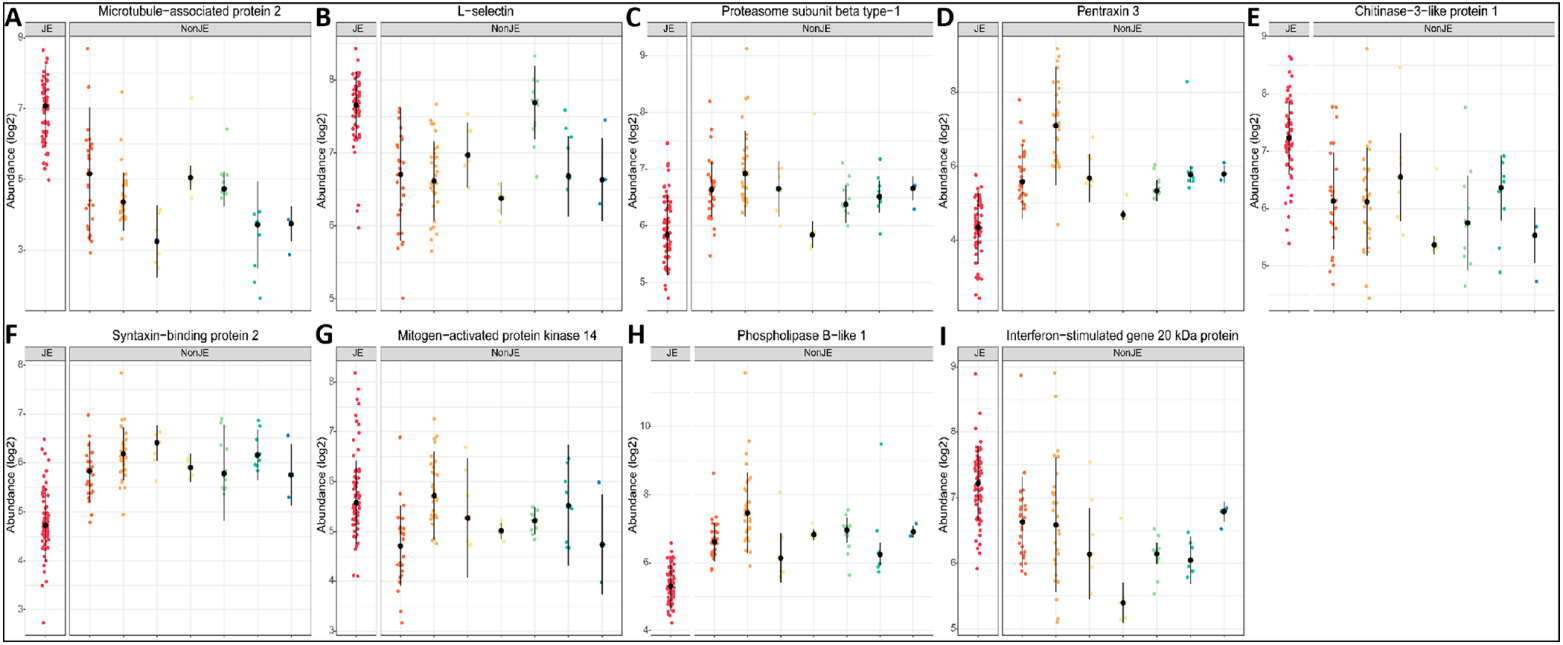
Differential expression across samples in nine proteins as a diagnostic CSF signature of JEV infection. Differential expression across samples in nine proteins as a diagnostic signature of Japanese encephalitis virus infection. Expression profiles of the proteins, microtubule-associated protein 2/P11137/MAP2 (A), L-selectin/P14151/SELL (B), Proteasome subunit beta type-1/P20618/PMSB-1 (C), Pentraxin-3/P26022/PTX3 (D), Chitinase-3-like protein 1/P36222/CH3L1 (E), Syntaxin-binding protein 2/Q15833/STXBP2 (F), Mitogen-activated protein kinase 14/Q16539/MAPK14 (G), Phospholipase B-like 1/Q6P4A8/PLBD1 (H), and Interferon-stimulated gene 20 kDa protein/Q96AZ6/ISG20 (I), are indicated for the categories virus (JE—red and non-JE—dark-orange), bacteria (mainstream—light-orange, TB—yellow, Rickettsia spp.—light-green, *Orientia tsutsugamushi*—dark-green), fungi (turquoise) and parasites (blue).

Feature selection identified a final set of nine proteins which together exhibited high predictive performance (Figure 6). When examined using the ensemble model, using 10-fold cross-validation, JE classification demonstrated an AUC-ROC of 98.7 (98.0–99.4), in addition to high sensitivity and specificity—metrics in Table 2 and ROC in Supplementary Data S13 and S14.

Data acquired by DIA LC–MS/MS of 16 samples were used to verify the nine-protein JE diagnostic predictive model. The test metrics are reported in Table 2.

### Establishing CSF Molecular Signatures as Predictors of the JE Outcome

#### Feature Selection

Subgroup analysis was performed using 42 JE samples for which outcome data at hospital discharge (died vs alive) were available. Seven proteins were identified as important in predicting outcome using the Boruta algorithm and two proteins using Lasso, such that two proteins were identified by both Boruta and Lasso, see Supplementary Data S15. In view of the small sample size, the data were not split into a training and test set. These proteins were used to train different models with fivefold CV repeated ten times evaluated on ROC and then combined in an ensemble model with cross-validation scores reported in Table 2, see the list of proteins in Supplementary Data S15 and ROC in Data S16. There were five JE patients in the DIA LC–MS analysis of which 3 had outcome data, and this was considered too small to report test metrics.

## Conclusions

We performed deep untargeted analysis of well-characterized patient CSF samples from a large number of different confirmed neurological infections. To our knowledge, the highest number of proteins in CSF identified to date has been 3,174;^48^ thus, this research represents a notable improvement in terms of the numbers of proteins identified, and this serves as a marker of the depth of analysis and prospects for biomarker identification.^49^ Offline fractionation into 90 fractions in the pilot study, and 100 fractions concatenated into 44 in the larger study, with two-hour online LC gradients and multiplexing with TMT-16plex contributed to the depth of analysis. Furthermore, the diverse range of neurological infections also augmented the variety of proteins identified.

WGCNA identified 20 clusters of highly correlated proteins and provided insight into the proteins and how they associate with disease mechanisms. The modules were allocated a descriptor, according to gene ontology analysis, as well as the clinical and biological significance of the proteins. For example, one module was associated with IgM (proteins in the module included immunoglobulin heavy constant mu and immunoglobulin J chain) and correlated with JE and *Orientia tsutsugamushi* (OT), as well as the duration of illness. Other important modules associated with upregulation in JE included neuronal damage, antiapoptosis, heat shock response, unfolded protein response, cell adhesion, and macrophage and dendritic cell activation. In contrast, in comparison to other non-JE neurological infections, there was an association with downregulated acute inflammatory response, hepatotoxicity, activation of coagulation, extracellular matrix, and actin regulation.

Predictive modeling using the nine-protein ensemble model enabled classification of JE and non-JE samples with a CV accuracy of 97.0 (95% CI 95.7–98.0) using TMT labeled DDA data, and 81.3% (95% CI 54.4–96.0) in verification with 16 (10%) of the samples by DIA. DIA is a label-free method of analysis, with ongoing improvements in depth and throughput; in this case providing a complementary method to verify the TMT data rather than performing traditional targeted LC–MS/MS proteomics such as parallel reaction monitoring. Three proteins selected as the best disease classifiers were not “significant”, i.e., *p* value <0.05 with *t*-test and adjustment for multiple testing, highlighting the limitations of univariate analysis in biomarker identification.^50^ Biomarker discovery is a lengthy process, akin to the pharmaceutical pipeline.^13^ The work demonstrates important CSF proteins in classifying JE vs non-JE. However, there is no doubt that the protein signature needs to be validated with orthogonal antibody-based methods in additional patient groups. It will also be useful to compare this with protein profiling in other body fluids. This will inform the use of a smaller subset of proteins in an ELISA or rapid diagnostic test to be tested alongside the existing anti-JEV IgM assay.

To date, to our knowledge, two studies have utilized unbiased techniques to examine the CSF proteome in human patients with confirmed JEV infection; while they demonstrate the feasibility of the methods, the patients were not confirmed by seroneutralization and included relatively small numbers of patients (10 and 26 JE patients).^51,52^ There have been a handful of studies utilizing ELISA methods to target specific proteins; however, these rarely used power calculations in their experimental design, nor did they include adequate controls.^53−58^ Analysis of the transcriptome and proteome in animal models^59−63^ and cell culture^53,59,64−69^ have been performed; however, the comparability to human CSF and comparison with other neurological infections is limited. Furthermore, mRNA expression does not directly correlate with that of the corresponding protein.^70^

As expected, while we included JEV proteins in the search database, we did not identify any JEV proteins. This is compatible with previous publications; non-structural protein 1 is the major secreted protein during flavivirus infections, harnessed widely as a diagnostic biomarker for dengue virus infection, but not a useful diagnostic biomarker for JE.^71^ The data provide useful interrogation of the host response to JEV infection. The identified proteins fit well into the existing literature on the host response in JEV and other closely associated flavivirus infections, most importantly West Nile virus infection.^72,73^ MAPT and MAP2 are both closely associated microtubule stabilizing proteins specific to neuronal cells.^74^ Both proteins were identified in this study as being biomarkers of JE in CSF, and the high levels in comparison to other neurological infections are striking. The association of the former has previously been demonstrated by ELISA, in one of the only studies of this type.^75^ The role of actin, microtubule, and intermediate filament cytoskeletal re-organization in flavivirus infection has been described^76^ and upregulation of MAPT and MAP2 may represent neuronal damage following transneural spread of JEV. Other proteins that were associated with JE in this study, all within the red WGCNA module, that may reflect neuronal damage include Paralemmin, Calbindin 1, MAP2, Parvalbumin, Secernin 1, and cell cycle exit and neuronal differentiation. The upregulation of ISG15 and ISG20 fit in with the known upregulation of a host of ISGs as part of the innate immune response to a viral infection.^77,78^ Additional functional enrichments reflecting different WGCNA modules have previously been described anti-apoptosis,^79^ heat shock response,^80,81^ unfolded protein response,^82^ translation,^83^ IgM,^84^ cell adhesion and pathogen attachment,^85^ endothelial activation,^86^ and macrophage activation.^87,88^ In comparison to other neurological infections, there was a downregulation in acute phase response proteins and neutrophil enriched proteins, as has been seen by other studies.^89−91^ In these, however, the sample size for the analysis of proteins predictive of outcome was less substantial and not supported by an a priori power calculation.

Incomplete coverage and missing data between LC–MS runs is an ongoing issue in the field.^32^ It is notable that comparing with other similar studies in the literature, the important proteins may not be exactly the same but are closely related. These issues are now being improved by DIA methods. Further limitations are that the demographics of the cases and controls were not perfectly matched and that we did not include CSF from healthy people in Laos on ethical grounds, or from cohorts from elsewhere on the basis that samples that have undergone different storage conditions may not be comparable. The latter is also the reason that there are no samples from neurological flaviviruses occurring in other geographical areas, such as West Nile virus (WNV) and Zika virus (ZIKV). Furthermore, for the purposes of the objective of finding a diagnostic protein signature of JE, the utmost importance was comparing JE with controls of a wide range of other neurological infections. The analysis of proteins predictive of different categories of infectious etiologies was not sufficiently powered and has not been reported. It is important to keep in mind that the comparison is between different neurological infections in the analysis of proteins that are up- and downregulated.

An RDT to detect JE in less accessible areas is urgently needed. This study demonstrates the feasibility of an unbiased LC–MS approach in the identification of novel protein biomarkers of neurological infections. It also represents a novel workflow for biomarker discovery research involving a large set of patient samples processed by TMT LC–MS/MS and then verified by an alternative DIA LC–MS/MS pipeline. Additional data using antibody-based methods will allow the nine-protein signature to be refined. This could be performed by purchasing or developing ELISA assays and comparing the specific protein abundance in JE and non-JE patients. These data will need to be validated in a larger group of patients, in different locations and in field settings. Ultimately, this could enable the selection of 2–3 proteins for the development of an RDT such as a lateral flow test. We would envisage, as per WHO guidelines, that any testing for JE will be performed contemporaneously with testing for Dengue virus infection and any other endemic flavivirus such as ZIKV and WNV.

## Supporting Information Available

S1_Data: LC–MS methods; S2_Patient metadata: Detailed table of the patient metadata; S3_Patient metadata: Summary of included patients’ demographics, clinical presentations and details of diagnosis separated into training and test groups; S4_MSstatsTMT output: Results of MSstatsTMT analysis for the pilot (tab 1) and larger (tab 2) study; S5_Contaminants: List of potential protein contaminant proteins that were excluded from downstream analysis; S6_Combined data processed: Normalized and batch corrected data from the pilot and larger study; S7: Assessment of data processing and effectiveness of batch correction; S8_ JE vs non-JE protein set enrichment analysis: Results of protein set enrichment analysis performed using the WebGestalt online tool; S9_WGCNA sample clustering to detect outliers: WGCNA hierarchical clustering of the samples to identify outliers; S10_WGCNA output: Results of WGCNA analysis; S11 WGCNA summary of modules: WGCNA eigengene dendrogram to illustrate groups of correlated modules; S12 Boruta Lasso proteins: List of proteins identified as important in the classification of JE vs non-JE by either Boruta or Lasso algorithms; S13_Predictive modeling to identify a JE diagnostic protein signature; S14_ROC: Receiver operator characteristic curve of the nine-protein model built to classify JE and non-JE patients; S15_Boruta proteins outcome: List of proteins identified as important in predicting the outcome (dead vs alive) of JE patients; and S16 ROC outcome: Receiver operator characteristic curve of the model built to predict the outcome (dead vs alive) of JE patients (ZIP)

## Notes

Ethical clearance for the Laos CNS study was granted by the Ethical Review Committee of the former Faculty of Medical Sciences, National University of Laos (now University of Health Sciences) and the Oxford University Tropical Ethics Research Committee, Oxford, UK.

## Notes

The mass spectrometry proteomics data have been deposited to the ProteomeXchange Consortium^92^ via the PRIDE partner repository with the dataset identifier PXD034789 and 10.6019/PXD034789. All other data underlying this article are available in the article and in its online Supplementary Material.
