# Supplementary material for: Deep Proteomics Network and Machine Learning Analysis of Human Cerebrospinal Fluid in Japanese Encephalitis Virus Infection
Source: J Proteome Res. 2023 May 23;22(6):1614–29. doi: 10.1021/acs.jproteome.2c00563 (PMC10246887; doi:10.1021/acs.jproteome.2c00563)
Supplement: Supplementary file 1 — pr2c00563_si_001.zip [file pr2c00563_si_001.zip › S1_Data.docx]

S1 Data: LC-MS methods

Table 1: Concatenation of low pH reverse phase fractions into samples run by LC-MS

| Samples | Time collected (min) | Time collected (min) | Time collected (min) |
| --- | --- | --- | --- |
| Sample 1 | 1 | 2 | 52 |
| Sample 2 | 3 | 4 | 53 |
| Sample 3 | 5 | 6 | 54 |
| Sample 4 | 7 | 8 | 55 |
| Sample 5 | 9 | 10 | 56 |
| Sample 6 | 11 | 12 | 57 |
| Sample 7 | 13 | 14 | 58 |
| Sample 8 | 15 | 59 |  |
| Sample 9 | 16 | 60 |  |
| Sample 10 | 17 | 61 |  |
| Sample 11 | 18 | 62 |  |
| Sample 12 | 19 | 63 |  |
| Sample 13 | 20 | 64 |  |
| Sample 14 | 21 | 65 |  |
| Sample 15 | 22 | 66 |  |
| Sample 16 | 23 | 67 |  |
| Sample 17 | 24 | 68 |  |
| Sample 18 | 25 | 69 |  |
| Sample 19 | 26 | 70 |  |
| Sample 20 | 27 | 71 |  |
| Sample 21 | 28 | 72 |  |
| Sample 22 | 29 | 73 |  |
| Sample 23 | 30 | 74 |  |
| Sample 24 | 31 | 75 |  |
| Sample 25 | 32 | 76 |  |
| Sample 26 | 33 | 77 |  |
| Sample 27 | 34 | 78 |  |
| Sample 28 | 35 | 79 |  |
| Sample 29 | 36 | 80 |  |
| Sample 30 | 37 | 81 |  |
| Sample 31 | 38 | 82 |  |
| Sample 32 | 39 | 83 |  |
| Sample 33 | 40 | 84 |  |
| Sample 34 | 41 | 85 |  |
| Sample 35 | 42 | 86 |  |
| Sample 36 | 43 | 87 |  |
| Sample 37 | 44 | 88 |  |
| Sample 38 | 45 | 89 |  |
| Sample 39 | 46 | 90 |  |
| Sample 40 | 47 | 91 | 92 |
| Sample 41 | 48 | 93 | 94 |
| Sample 42 | 49 | 95 | 96 |
| Sample 43 | 50 | 97 | 98 |
| Sample 44 | 51 | 99 | 100 |

Table 2: Gradient used for offline high pH reverse-phase fractionation

| Pilot study: | |
| --- | --- |
| Time (min.) | % B |
| 0 | 2 |
| 70 | 60 |
| 80 | 90 |
| 90 | 95 |
| 98 | 95 |
| 99 | 2 |
| 109 | 2 |
|  | |
| Larger study: | |
| Time (min.) | % B |
| 0 | 2 |
| 12 | 2 |
| 72 | 45 |
| 80 | 95 |
| 90 | 95 |
| 92 | 2 |
| 100 | 2 |

Table 3: Gradient used for online nanoflow liquid chromatography

| Pilot study: | |
| --- | --- |
| Time (min.) | % B |
| 0 | 2 |
| 4.6 | 2 |
| 105.0 | 36 |
| 125.0 | 60 |
| 126.0 | 95 |
| 131.0 | 95 |
| 132.0 | 2 |
| 147.0 | 2 |
|  | |
| Larger study: | |
| Time (min.) | % B |
| 0 | 4 |
| 4.5 | 4 |
| 10 | 8 |
| 105 | 36 |
| 125 | 60 |
| 126 | 98 |
| 131 | 98 |
| 132 | 4 |
| 133 | 98 |
| 134 | 98 |
| 135 | 4 |
| 138 | 98 |
| 141 | 98 |
| 144 | 4 |
| 145 | 98 |
| 146 | 4 |
| 171 | 4 |

Table 4: Mass spectrometry settings

| Pilot study: |
| --- |
| MS nano-flow tune file settings  Spray voltage (kV) 2.4, capillary temp (^o^C): 275.  Global settings  Lock mass: best, Chrom peak width: 20 s, Time duration: 147.10 min.  General settings  Run time 0-147.1 min., polarity: +ve, in-source CID: 0.0 ev, default charge: 2.  Full-MS settings  Microscans: 1, resolution: 70,000, AGC target: 3e6, maximum IT: 60 ms, number of scans: 1, scan range: 375 to 1500 m/z, spectrum data type: profile.  Data dependent (DD) MS^2^ settings  Microscans: 1, resolution: 35,000, AGC target: 1e5, maximum IT: 60 ms, loop count: 10, MSX count: 1, TopN: 10, isolation window: 2.0 m/z, isolation offset: 0.0 m/z, fixed first mass: 120.0 m/z, NCE/stepped nce: 27 spectrum data type: profile.  DD settings  Minimum AGC target: 2.00e3, intensity threshold: 3.3e4, apex trigger: none, charge exclusion: unassigned, peptide match: preferred, exclude isotope: on, dynamic exclusion: 30s. |
|  |
| Larger study: |
| MS nano-flow tune file settings  Spray voltage (kV) 1.9, capillary temp (^o^C): 275.  Global settings  Lock mass: best, Chrom peak width: 20 s, Time duration: 171 min.  General settings  Run time 0-171 min., polarity: +ve, in-source CID: 0.0 ev, default charge: 2.  Full-MS settings  Microscans: 1, resolution: 70,000, AGC target: 3e6, maximum IT: 50 ms, number of scans: 1, scan range: 350 to 1500 m/z, spectrum data type: profile.  Data dependent (DD) MS^2^ settings  Microscans: 1, resolution: 35,000, AGC target: 2e5, maximum IT: 250 ms, loop count: 10, MSX count: 1, TopN: 10, isolation window: 1.2 m/z, isolation offset: 0.0 m/z, fixed first mass: 110.0 m/z, NCE/stepped nce: 30, spectrum data type: profile.  DD settings  Minimum AGC target: 5.00e3, intensity threshold: 2.0e4, apex trigger: none, charge exclusion: unassigned, peptide match: preferred, exclude isotope: on, dynamic exclusion: 45s. |
