# Supplementary material for: Deep Proteomics Network and Machine Learning Analysis of Human Cerebrospinal Fluid in Japanese Encephalitis Virus Infection
Source: J Proteome Res. 2023 May 23;22(6):1614–29. doi: 10.1021/acs.jproteome.2c00563 (PMC10246887; doi:10.1021/acs.jproteome.2c00563)
Supplement: Supplementary file 1 — pr2c00563_si_001.zip [file pr2c00563_si_001.zip › S13_Predictive modelling to identify a JE diagnostic protein signature.docx]

S13: Predictive modelling to identify a JE diagnostic protein signature

Table 1: Predictive modelling scores with 95% confidence intervals for the TMT-labelled study with the samples included in the DIA study removed as a training set and the DIA study as a test set^1^

| Features selected | Number of proteins | Data | AUC-ROC | Accuracy | Sensitivity | Specificity | Positive predictive value | Negative predictive value | Top 2 proteins in the model |
| --- | --- | --- | --- | --- | --- | --- | --- | --- | --- |
| All^2^ | 2176 | Training set  (n=147) | 100 (100-100) | 100 (99.6-100) | 100 (99.3-100) | 100 (99.3-100) | 100 (99.3-100) | 100 (99.3-100) | P11137, Q6P4A8 |
|  | 1736 | Training set (n=147) | 100 (100-100) | 100 (99.6-100) | 100 (99.3-100) | 100 (99.3-100) | 100 (99.3-100) | 100 (99.3-100) | Q6P4A8, Q15833 |
|  |  | Test set  (n=16) | 77.3 (61.8-92.7) | 62.5 (35.4-84.8) | 100 (47.8-100) | 45.5 (16.7-76.6) | 45.5 (16.7-76.6) | 100 (47.8-100.0) | - |
| Boruta^3^ | 68 | Training set (n=147) | 97.4 (96.4-98.4) | 95.4 (93.9-9661) | 96.0 (93.9-97.5) | 94.8 (92.5-96.6) | 94.9 (92.6-96.6) | 96.0 (93.8-97.5) | Q6P4A8, Q15833 |
|  | 64 | Training set (n=147) | 99.7 (99.4-99.9) | 95.2 (93.4-96.4) | 96.0 (93.9-97.5) | 94.4 (92.0-96.2) | 94.5 (92.1-96.3) | 95.9 (93.8-97.5) | Q6P4A8, Q15833 |
|  |  | Test set  (n=16) | 32.7 (11.4-54.1) | 62.5 (35.4-84.8) | 100 (47.8-100) | 45.5 (16.7-76.6) | 45.5 (16.7-76.6) | 100 (47.8-100) | - |
| Lasso^4^ | 24 | Training set (n=147) | 100 (100-100) | 100 (99.6-100) | 100 (99.3-100) | 100 (99.3-100) | 100 (99.3-100) | 100 (99.3-100) | Q6P4A8, Q15833 |
|  | 20 | Training set (n=147) | 100 (100-100) | 100 (99.6-100) | 100 (99.3-100) | 100 (99.3-100) | 100 (99.3-100) | 100 (99.3-100) | Q6P4A8, Q15833 |
|  |  | Test set  (n=16) | 74.5 (48.4-100) | 81.3 (54.4-96.0) | 100 (47.8-100) | 72.7 (39.0-94.0) | 62.5 (24.5-91.5) | 100 (63.1-100) | - |
| Intersect | 9 | Training set (n=147) | 98.7 (98.0-99.4) | 97.0 (95.7-98.0) | 99.8 (98.9-100) | 94.2 (91.8-96.1) | 94.5 (92.2-96.3) | 99.8 (98.8-100) | Q15833,  P11137 |
|  |  | Test set  (n=16) | 95.5 (86.6-100) | 81.3 (54.4-96.0) | 100 (47.8-100) | 72.7 (39.0-94.0) | 62.5 (24.5-91.5) | 100 (63.1-100) | - |
| Top 2 proteins  Intersect | 2 | Training set (n=147) | 97.4 (96.2-98.6) | 95.6 (94.1-97.0) | 95.6 (93.4-97.2) | 95.6 (93.4-97.2) | 95.6 (93.4-97.2) | 95.6 (93.4-97.2) | Q15833,  P11137 |
|  |  | Test set  (n=16) | 96.4 (88.0-100) | 68.8 (41.3-89.0) | 100 (47.8-100) | 54.5 (23.4-83.3) | 50.0 (18.7-81.3) | 100 (54.1-100) | - |
| Top 2 proteins  all | 2 | Training set (n=147) | 95.0 (93.3-96.8) | 96.6 (95.3-97.6) | 97.6 (95.8-98.8) | 95.6 (93.4-97.2) | 95.7 (93.5-97.3) | 97.6 (95.8-98.7) | P11137, Q6P4A8 |
|  |  | Test set  (n=16) | 79 (43.0-98.9) | 37.5 (15.2-64.6) | 100 (47.8-100) | 9.1 (0.2-41.3) | 33.3 (11.8-61.6) | 100 (2.5-100) | - |

1. An ensemble model was built using random forest, support vector machine, generalised linear model and naïve bayes, except for the modelling with all the data in which the naïve bayes model failed and the ensemble model was built the other three methods. The training set included patient samples (63 JE and 84 non-JE confirmed neurological infections) processed by TMT LC-MS/MS. 4. The test set included 10% of the patient samples from the TMT LC-MS/MS analysis processed separately by label-free DIA LC-MS/MS.

2. 2176 proteins were included in the final processed TMT LC-MS/MS dataset, however 440 were not present in the DIA data, and a model was built consisting of 1736 proteins.

3. 68 proteins were identified by the Boruta algorithm, however 4 of these were not present in the DIA data, and that a model was built consisting of 64 proteins.

4. 24 proteins were identified by Lasso, however 4 of these were not present in the DIA data, and that a model was built consisting of 20 proteins.
