# Supplementary material for: Deep Proteomics Network and Machine Learning Analysis of Human Cerebrospinal Fluid in Japanese Encephalitis Virus Infection
Source: J Proteome Res. 2023 May 23;22(6):1614–29. doi: 10.1021/acs.jproteome.2c00563 (PMC10246887; doi:10.1021/acs.jproteome.2c00563)
Supplement: Supplementary file 1 — pr2c00563_si_001.zip [file pr2c00563_si_001.zip › S3_Patient metadata_summary.docx]

S3_Summary of included patients’ demographics, clinical presentations and details of diagnosis separated into training and test groups.

Table 1: Training set

|  |  | **Virus_**  **JE**  **(N=63)** | **Virus_**  **NonJE (N=26)** | **Bacteria_**  **main**  **(N=25)** | **Bacteria_**  **TB**  **(N=6)** | **Bacteria_**  **Rickettsia (N=5)** | **Bacteria_**  **OT**  **(N=12)** | **Fungi**  **(N=7)** | **Parasite**  **(N=3)** | **Total**  **(N=147)** |
| --- | --- | --- | --- | --- | --- | --- | --- | --- | --- | --- |
| Age (yrs) | Median | 16.0 | 21.0 | 42.0 | 53.0 | 48.0 | 20.0 | 45.0 | 25.0 | 22.0 |
|  | IQR | 10.0, 22.5 | 5.0, 36.5 | 21.0, 55.0 | 47.0, 56.8 | 32.0, 53.0 | 11.8, 24.0 | 29.5, 48.5 | 24.5, 42.0 | 11.5, 41.5 |
| Sex | Female | 24 (38.1%) | 10 (38.5%) | 8 (32.0%) | 1 (16.7%) | 1 (20.0%) | 4 (33.3%) | 0 (0.0%) | 0 (0.0%) | 48 (32.7%) |
| Ethnicity | Lao loum | 34 (54.0%) | 25 (96.2%) | 19 (76.0%) | 6 (100.0%) | 5 (100.0%) | 11 (91.7%) | 6 (85.7%) | 3 (100.0%) | 109 (74.1%) |
|  | Lao sung | 14 (22.2%) | 1 (3.8%) | 2 (8.0%) | 0 (0.0%) | 0 (0.0%) | 0 (0.0%) | 0 (0.0%) | 0 (0.0%) | 17 (11.6%) |
|  | Lao theung | 1 (1.6%) | 0 (0.0%) | 0 (0.0%) | 0 (0.0%) | 0 (0.0%) | 0 (0.0%) | 0 (0.0%) | 0 (0.0%) | 1 (0.7%) |
|  | Lao unspecified | 14 (22.2%) | 0 (0.0%) | 4 (16.0%) | 0 (0.0%) | 0 (0.0%) | 1 (8.3%) | 1 (14.3%) | 0 (0.0%) | 20 (13.6%) |
| Comorbidities | N-Miss | 0 | 0 | 0 | 0 | 0 | 1 | 0 | 0 | 1 |
|  | Yes | 3 (4.8%) | 4 (15.4%) | 3 (12.0%) | 4 (66.7%) | 1 (20.0%) | 0 (0.0%) | 3 (42.9%) | 0 (0.0%) | 18 (12.2%) |
|  | HIV | 0 (0.0%) | 2 (7.7%) | 0 (0.0%) | 0 (0.0%) | 0 (0.0%) | 0 (0.0%) | 3 (42.9%) | 0 (0.0%) | 5 (3.4%) |
| Duration of illness (days) | Median | 4.0 | 3.5 | 3.0 | 6.5 | 2.0 | 5.5 | 6.0 | 1.0 | 4.0 |
|  | IQR | 4.0, 6.0 | 1.2, 5.8 | 2.0, 5.0 | 3.8, 7.0 | 2.0, 4.0 | 2.8, 8.2 | 1.5, 7.0 | 0.5, 4.0 | 2.5, 6.0 |
| Seizures | Yes | 26 (41.3%) | 9 (34.6%) | 6 (24.0%) | 0 (0.0%) | 2 (40.0%) | 3 (25.0%) | 0 (0.0%) | 0 (0.0%) | 46 (31.3%) |
| Antibiotics prior to LP | N-Miss | 11 | 1 | 2 | 2 | 0 | 3 | 2 | 0 | 21 |
|  | Yes | 41 (78.8%) | 15 (60.0%) | 9 (39.1%) | 3 (75.0%) | 2 (40.0%) | 8 (88.9%) | 0 (0.0%) | 1 (33.3%) | 79 (62.7%) |
| GCS | N-Miss | 7 | 0 | 0 | 0 | 0 | 1 | 0 | 0 | 8 |
|  | Median | 11.0 | 13.5 | 12.0 | 11.0 | 10.0 | 15.0 | 15.0 | 15.0 | 12.0 |
|  | IQR | 7.8, 13.0 | 9.2, 15.0 | 10.0, 15.0 | 9.2, 12.8 | 7.0, 14.0 | 14.0, 15.0 | 13.5, 15.0 | 15.0, 15.0 | 9.0, 15.0 |
| Blood WCC (10^6/uL) | N-Miss | 9 | 1 | 4 | 0 | 0 | 1 | 0 | 0 | 15 |
|  | Median | 12.4 | 11.1 | 10.6 | 11.5 | 8.3 | 13.0 | 11.0 | 7.5 | 11.5 |
|  | IQR | 8.4, 16.8 | 7.8, 13.0 | 8.4, 17.1 | 9.6, 12.3 | 7.0, 13.0 | 10.0, 13.6 | 6.6, 14.8 | 7.4, 8.9 | 8.0, 14.9 |
| Blood CRP (mg/L) | N-Miss | 45 | 10 | 11 | 4 | 1 | 5 | 1 | 1 | 78 |
|  | Median | 45.0 | 45.1 | 184.5 | 20.3 | 41.6 | 102.5 | 33.5 | 9.8 | 46.7 |
|  | IQR | 24.7, 73.1 | 4.5, 93.1 | 106.3, 196.5 | 16.5, 24.0 | 3.9, 103.4 | 33.6, 131.3 | 10.6, 45.5 | 5.1, 14.6 | 19.4, 112.6 |
| CSF opening pressure (cm of H_2_0) | N-Miss | 10 | 3 | 0 | 0 | 0 | 1 | 0 | 0 | 14 |
|  | Median | 23.0 | 20.0 | 25.0 | 24.8 | 15.0 | 23.0 | 36.0 | 27.0 | 23.0 |
|  | IQR | 18.5, 26.0 | 15.5, 28.2 | 11.5, 35.0 | 16.1, 29.2 | 11.5, 20.0 | 20.0, 25.0 | 18.8, 39.5 | 22.5, 28.0 | 17.0, 29.0 |
| CSF colour | N-Miss | 4 | 1 | 3 | 0 | 0 | 4 | 0 | 1 | 13 |
|  | Clear | 45 (76.3%) | 17 (68.0%) | 5 (22.7%) | 5 (83.3%) | 3 (60.0%) | 7 (87.5%) | 5 (71.4%) | 1 (50.0%) | 88 (65.7%) |
|  | Red | 1 (1.7%) | 3 (12.0%) | 1 (4.5%) | 0 (0.0%) | 1 (20.0%) | 0 (0.0%) | 0 (0.0%) | 0 (0.0%) | 6 (4.5%) |
|  | Turbid | 13 (22.0%) | 4 (16.0%) | 14 (63.6%) | 1 (16.7%) | 0 (0.0%) | 0 (0.0%) | 2 (28.6%) | 1 (50.0%) | 35 (26.1%) |
|  | Yellow | 0 (0.0%) | 1 (4.0%) | 2 (9.1%) | 0 (0.0%) | 1 (20.0%) | 1 (12.5%) | 0 (0.0%) | 0 (0.0%) | 5 (3.7%) |
| CSF WCC (10^6/L) | N-Miss | 3 | 2 | 2 | 1 | 2 | 1 | 1 | 0 | 12 |
|  | Median | 115.0 | 147.5 | 640.0 | 260.0 | 25.0 | 60.0 | 140.0 | 500.0 | 145.0 |
|  | IQR | 48.8, 215.0 | 36.2, 255.0 | 172.5, 2297.5 | 125.0, 335.0 | 17.5, 35.0 | 47.5, 132.5 | 32.5, 307.5 | 492.5, 1115.0 | 52.5, 325.0 |
| CSF % neutrophils | N-Miss | 3 | 2 | 2 | 1 | 2 | 2 | 1 | 0 | 13 |
|  | Median | 24.0 | 29.0 | 90.0 | 27.0 | 89.0 | 67.0 | 100.0 | 72.0 | 42.6 |
|  | IQR | 9.3, 46.2 | 12.4, 62.8 | 67.5, 97.2 | 20.0, 33.3 | 69.5, 94.5 | 58.9, 78.0 | 88.0, 100.0 | 66.5, 85.0 | 14.3, 89.0 |
| CSF % lymphocytes | N-Miss | 3 | 2 | 2 | 1 | 2 | 2 | 1 | 0 | 13 |
|  | Median | 76.0 | 71.0 | 10.0 | 73.0 | 11.0 | 33.0 | 0.0 | 28.0 | 57.4 |
|  | IQR | 53.8, 91.4 | 37.2, 87.6 | 2.8, 32.5 | 66.7, 80.0 | 5.5, 30.5 | 22.0, 41.1 | 0.0, 12.0 | 15.0, 33.5 | 11.0, 85.7 |
| CSF RCC (10^6/L) | N-Miss | 2 | 0 | 3 | 0 | 0 | 0 | 0 | 0 | 5 |
|  | Median | 0.0 | 0.0 | 2.0 | 0.0 | 75.0 | 14.5 | 10.0 | 75.0 | 0.0 |
|  | IQR | 0.0, 0.0 | 0.0, 28.8 | 0.0, 193.8 | 0.0, 18.8 | 5.0, 1005.0 | 0.0, 37.5 | 2.5, 24.0 | 37.5, 85.0 | 0.0, 28.8 |
| CSF total protein (mg/dL) | N-Miss | 6 | 1 | 1 | 0 | 1 | 2 | 0 | 0 | 11 |
|  | Median | 60.0 | 63.0 | 135.0 | 196.0 | 72.5 | 93.5 | 39.0 | 96.0 | 75.5 |
|  | IQR | 38.0, 88.0 | 30.0, 119.0 | 58.8, 242.2 | 95.8, 273.0 | 60.0, 75.2 | 57.8, 139.5 | 26.5, 65.0 | 59.0, 108.0 | 38.0, 121.5 |
| CSF glucose (mmol/L) | N-Miss | 4 | 2 | 1 | 0 | 1 | 2 | 0 | 0 | 10 |
|  | Median | 3.7 | 3.2 | 2.7 | 2.7 | 5.1 | 3.4 | 2.9 | 2.9 | 3.5 |
|  | IQR | 2.9, 4.8 | 1.9, 5.1 | 1.8, 4.7 | 1.5, 5.2 | 3.7, 5.8 | 3.1, 6.5 | 2.2, 3.0 | 2.6, 4.2 | 2.4, 5.3 |
| Duration of admission (days) | N-Miss | 32 | 3 | 4 | 0 | 1 | 3 | 3 | 0 | 46 |
|  | Median | 13.0 | 9.0 | 12.0 | 20.0 | 9.5 | 5.0 | 25.5 | 8.0 | 11.0 |
|  | IQR | 8.5, 17.0 | 7.0, 12.5 | 5.0, 16.0 | 11.8, 22.2 | 6.5, 12.0 | 5.0, 8.0 | 20.2, 30.0 | 7.5, 10.0 | 7.0, 16.0 |
| Outcome | N-Miss | 24 | 3 | 4 | 0 | 1 | 2 | 4 | 0 | 38 |
|  | Died | 9 (23.1%) | 2 (8.7%) | 7 (33.3%) | 1 (16.7%) | 0 (0.0%) | 1 (10.0%) | 0 (0.0%) | 0 (0.0%) | 20 (18.3%) |

Table 2: Test set

|  |  | **Virus_**  **JE**  **(N=5)** | **Virus_**  **NonJE (N=1)** | **Bacteria_**  **main**  **(N=7)** | **Bacteria_**  **TB**  **(N=1)** | **Bacteria_**  **Rickettsia (N=0)** | **Bacteria_**  **OT**  **(N=0)** | **Fungi**  **(N=2)** | **Parasite**  **(N=0)** | **Total**  **(N=16)** |
| --- | --- | --- | --- | --- | --- | --- | --- | --- | --- | --- |
| Age (yrs) | Median | 40.0 | 25.0 | 28.0 | 20.0 |  |  | 26.0 |  | 27.0 |
|  | IQR | 19.0, 51.0 | 25.0, 25.0 | 18.0, 44.5 | 20.0, 20.0 |  |  | 22.5, 29.5 |  | 19.0, 42.8 |
| Sex | Female | 0 (0.0%) | 0 (0.0%) | 1 (14.3%) | 0 (0.0%) |  |  | 0 (0.0%) |  | 1 (6.2%) |
| Ethnicity | Lao loum |  |  |  |  |  |  |  |  |  |
|  | Lao sung | 1 (20.0%) | 0 (0.0%) | 4 (57.1%) | 1 (100.0%) |  |  | 2 (100.0%) |  | 8 (50.0%) |
|  | Lao theung | 2 (40.0%) | 0 (0.0%) | 1 (14.3%) | 0 (0.0%) |  |  | 0 (0.0%) |  | 3 (18.8%) |
|  | Lao unspecified | 0 (0.0%) | 0 (0.0%) | 1 (14.3%) | 0 (0.0%) |  |  | 0 (0.0%) |  | 1 (6.2%) |
| Comorbidities | N-Miss | 2 (40.0%) | 1 (100.0%) | 1 (14.3%) | 0 (0.0%) |  |  | 0 (0.0%) |  | 4 (25.0%) |
|  | Yes | 0 | 0 | 0 | 0 |  |  | 0 |  | 0 |
|  | HIV | 0 (0.0%) | 1 (100.0%) | 0 (0.0%) | 0 (0.0%) |  |  | 2 (100.0%) |  | 3 (18.8%) |
| Duration of illness (days) | Median | 0 (0.0%) | 1 (100.0%) | 0 (0.0%) | 0 (0.0%) |  |  | 2 (100.0%) |  | 3 (18.8%) |
|  | IQR | 3.0 | 1.0 | 2.0 | 1.0 |  |  | 3.5 |  | 2.5 |
| Seizures | Yes | 3.0, 4.0 | 1.0, 1.0 | 2.0, 5.5 | 1.0, 1.0 |  |  | 2.8, 4.2 |  | 2.0, 4.2 |
| Antibiotics prior to LP | N-Miss | 3 (60.0%) | 0 (0.0%) | 0 (0.0%) | 0 (0.0%) |  |  | 1 (50.0%) |  | 4 (25.0%) |
|  | Yes | 2 | 0 | 1 | 0 |  |  | 1 |  | 4 |
| GCS | N-Miss | 3 (100.0%) | 0 (0.0%) | 3 (50.0%) | 1 (100.0%) |  |  | 1 (100.0%) |  | 8 (66.7%) |
|  | Median | 0 | 0 | 1 | 0 |  |  | 0 |  | 1 |
|  | IQR | 11.0 | 15.0 | 12.5 | 4.0 |  |  | 12.0 |  | 12.0 |
| Blood WCC (10^6/uL) | N-Miss | 11.0, 13.0 | 15.0, 15.0 | 9.0, 14.5 | 4.0, 4.0 |  |  | 10.5, 13.5 |  | 8.5, 15.0 |
|  | Median | 0 | 0 | 1 | 0 |  |  | 0 |  | 1 |
|  | IQR | 16.0 | 12.6 | 16.2 | 8.2 |  |  | 4.4 |  | 12.8 |
| Blood CRP (mg/L) | N-Miss | 10.0, 19.0 | 12.6, 12.6 | 13.3, 26.4 | 8.2, 8.2 |  |  | 4.0, 4.8 |  | 9.1, 18.4 |
|  | Median | 5 | 1 | 7 | 1 |  |  | 2 |  | 16 |
|  | IQR | NA | NA | NA | NA |  |  | NA |  | NA |
| CSF opening pressure (cm of H_2_0) | N-Miss | NA | NA | NA | NA |  |  | NA |  | NA |
|  | Median | 1 | 0 | 0 | 0 |  |  | 0 |  | 1 |
|  | IQR | 20.5 | 19.0 | 20.0 | 24.0 |  |  | 14.5 |  | 19.0 |
| CSF colour | N-Miss | 16.8, 24.5 | 19.0, 19.0 | 13.2, 25.0 | 24.0, 24.0 |  |  | 12.8, 16.2 |  | 16.5, 24.5 |
|  | Clear | 0 | 0 | 0 | 0 |  |  | 0 |  | 0 |
|  | Red | 5 | 1 | 1 | 1 |  |  | 1 |  | 9 |
|  | Turbid |  |  |  |  |  |  |  |  |  |
|  | Yellow | 0 | 0 | 6 | 0 |  |  | 1 |  | 7 |
| CSF WCC (10^6/L) | N-Miss |  |  |  |  |  |  |  |  |  |
|  | Median | 0 | 1 | 0 | 0 |  |  | 0 |  | 1 |
|  | IQR | 75.0 | NA | 955.0 | 35.0 |  |  | 135.0 |  | 315.0 |
| CSF % neutrophils | N-Miss | 65.0, 315.0 | NA | 500.0, 1300.0 | 35.0, 35.0 |  |  | 85.0, 185.0 |  | 50.0, 727.5 |
|  | Median | 0 | 1 | 1 | 0 |  |  | 0 |  | 2 |
|  | IQR | 33.3 | NA | 96.0 | 100.0 |  |  | 57.9 |  | 76.4 |
| CSF % lymphocytes | N-Miss | 0.0, 72.6 | NA | 80.0, 99.2 | 100.0, 100.0 |  |  | 43.2, 72.6 |  | 41.0, 96.5 |
|  | Median | 0 | 1 | 1 | 0 |  |  | 0 |  | 2 |
|  | IQR | 66.7 | NA | 4.0 | 0.0 |  |  | 42.1 |  | 23.6 |
| CSF RCC (10^6/L) | N-Miss | 27.4, 100.0 | NA | 0.8, 20.0 | 0.0, 0.0 |  |  | 27.4, 56.8 |  | 3.5, 59.0 |
|  | Median | 0 | 0 | 0 | 0 |  |  | 0 |  | 0 |
|  | IQR | 0.0 | 0.0 | 0.0 | 0.0 |  |  | 0.0 |  | 0.0 |
| CSF total protein (mg/dL) | N-Miss | 0.0, 0.0 | 0.0, 0.0 | 0.0, 0.0 | 0.0, 0.0 |  |  | 0.0, 0.0 |  | 0.0, 0.0 |
|  | Median | 0 | 0 | 0 | 0 |  |  | 0 |  | 0 |
|  | IQR | 95.0 | 54.0 | 83.0 | 110.0 |  |  | 64.0 |  | 86.5 |
| CSF glucose (mmol/L) | N-Miss | 66.0, 108.0 | 54.0, 54.0 | 60.5, 339.0 | 110.0, 110.0 |  |  | 42.0, 86.0 |  | 53.2, 108.5 |
|  | Median | 0 | 0 | 1 | 0 |  |  | 0 |  | 1 |
|  | IQR | 3.4 | 3.3 | 3.6 | 1.4 |  |  | 1.4 |  | 3.3 |
| Duration of admission (days) | N-Miss | 3.0, 4.1 | 3.3, 3.3 | 2.0, 7.2 | 1.4, 1.4 |  |  | 1.1, 1.6 |  | 1.7, 4.0 |
|  | Median | 2 | 0 | 1 | 0 |  |  | 0 |  | 3 |
|  | IQR | 15.0 | 7.0 | 10.0 | 5.0 |  |  | 8.5 |  | 11.0 |
| Outcome | N-Miss | 13.0, 17.5 | 7.0, 7.0 | 7.5, 17.0 | 5.0, 5.0 |  |  | 6.2, 10.8 |  | 7.0, 15.0 |
|  | Died | 2 | 0 | 3 | 0 |  |  | 1 |  | 6 |
