# Supplementary material for: Deep Proteomics Network and Machine Learning Analysis of Human Cerebrospinal Fluid in Japanese Encephalitis Virus Infection
Source: J Proteome Res. 2023 May 23;22(6):1614–29. doi: 10.1021/acs.jproteome.2c00563 (PMC10246887; doi:10.1021/acs.jproteome.2c00563)
Supplement: Supplementary file 1 — pr2c00563_si_001.zip [file pr2c00563_si_001.zip › S7_Assessment of data processing and effectiveness of batch correction.docx]

# Supplementary Data 7: Investigation of data processing and the effectiveness of batch correction.

This document contains the R script and output of investigation of batch effects and correction from merging the pilot (n=15; JE=8 and non-JE=7) and larger (n=148; JE=60 and non-JE=88) TMT LC-MS/MS studies using the R package proBatch. <https://rdrr.io/bioc/proBatch/f/inst/doc/proBatch.pdf>

The pilot study involved the analysis of two batches of TMT 11-plex and the larger study included ten batches of TMT 16-plex. The pilot study included seven of the same samples repeated in each batch, while the larger study included a reference pool in each batch. Data processing for each dataset was performed in the same way (MSstatsTMT -> DreamAI -> jitter), except that for the larger study MSstatsTMT protein summarization was performed by normalising to the reference pool.

It was our impression that increasing the number of samples would improve the power of the study, and equally that incorporating two different datasets would reduce the overfitting in machine learning models. Nonetheless, we evaluated carefully for batch effects in the merged dataset, see below. Overall, the different types of figures incorporated in the analysis illustrate the necessity and effectiveness for the data processing applied (RobNorm for normalisation followed by ComBat for batch correction).

Loading required packages

bioc_deps <- c("GO.db", "impute", "preprocessCore", "pvca", "sva")

cran_deps <- c("corrplot", "data.table", "ggplot2", "ggfortify", "lazyeval", "lubridate",

"pheatmap", "reshape2", "readr", "rlang", "tibble", "dplyr", "tidyr", "wesanderson",

"WGCNA")

library(tidyverse)

library(proBatch)

library(limma)

library(readxl)

library(writexl)

library(caret)

library(lattice)

# A) Analysis of baseline uncorrected data

data_precorrection = as.data.frame(read_excel("data_jitter_combined_pre_RobNorm and ComBat.xlsx"))

table(rowSums(is.na(data_precorrection)))

##

## 0

## 2176

row.names(data_precorrection) = data_precorrection$Protein

data_precorrection$Protein = NULL

Import the metadata

metadata = read_excel("C:/Users/tehmi/Dropbox/Documents/PhD/Proteomics/TMT_Verification/Data analysis/Variables/Combined_patient_data.xlsx")

Creating an annotation file that aligns with the data and processing the data file to the correct format (colnames as samples, rownames as feature IDS, log2transformed)

metadata$jev[metadata$jev == 1] = "JEV"

metadata$jev[metadata$jev == 0] = "NonJEV"

metadata$Dataset[metadata$Dataset == 1] = "Pilot"

metadata$Dataset[metadata$Dataset == 2] = "Ver"

metadata$names = names(data_precorrection)

names = names(data_precorrection)

metadata = unite(metadata, "metadata", c("names", "Dataset", "jev", "category of infection"),

sep = ":")

names = metadata$metadata

names(data_precorrection) = names

annotation = data_precorrection

annotation = pivot_longer(data_precorrection, c(1:163), names_to = "FullRunName",

values_to = "Abundance")

annotation = tidyr::separate(annotation, FullRunName, c("Batch", "Replicate"), remove = FALSE,

sep = ";")

annotation = separate(annotation, Replicate, c("Channel", "Dataset", "JEV_nonJEV",

"Bio_rep"), remove = TRUE, sep = ":")

annotation = select(annotation, -c(7))

annotation = distinct(annotation, FullRunName, .keep_all = TRUE)

annotation$Batch = factor(annotation$Batch, levels = c("Pilot1", "Pilot2", "Pilot",

"Ver1", "Ver2", "Ver3", "Ver4", "Ver5", "Ver6", "Ver7", "Ver8", "Ver9", "Ver10"))

annotation$Channel = factor(annotation$Channel, levels = c("126", "127N", "127C",

"128N", "128C", "129N", "129C", "130N", "130C", "131N", "131C", "132N", "132C",

"133N", "133C", "134N"))

annotation$Dataset = as.factor(annotation$Dataset)

annotation$JEV_nonJEV = as.factor(annotation$JEV_nonJEV)

annotation$Bio_rep = as.factor(annotation$Bio_rep)

annotation = unite(annotation, "FullRunName", c("Batch", "Channel", "Bio_rep"), remove = FALSE)

names(data_precorrection) = annotation$FullRunName

Saving a dataframe in a wide format and long format

log_transformed_df = data_precorrection

log_transformed_long = matrix_to_long(log_transformed_df)

## Plotting the mean protein abundance for samples in each batch

plot_sample_mean(log_transformed_df, annotation, sample_id_col = "FullRunName", batch_col = "Batch",

color_by_batch = TRUE, order_col = "Batch", color_scheme = "brewer")

**Figure 1: Mean protein abundance of patient samples (y axis) categorised by batch (y axis).**

The figure demonstrates that there is a systematic difference in the mean protein abundances of patient samples analysed in the pilot (Pilot) study as compared to the larger (Ver) study.


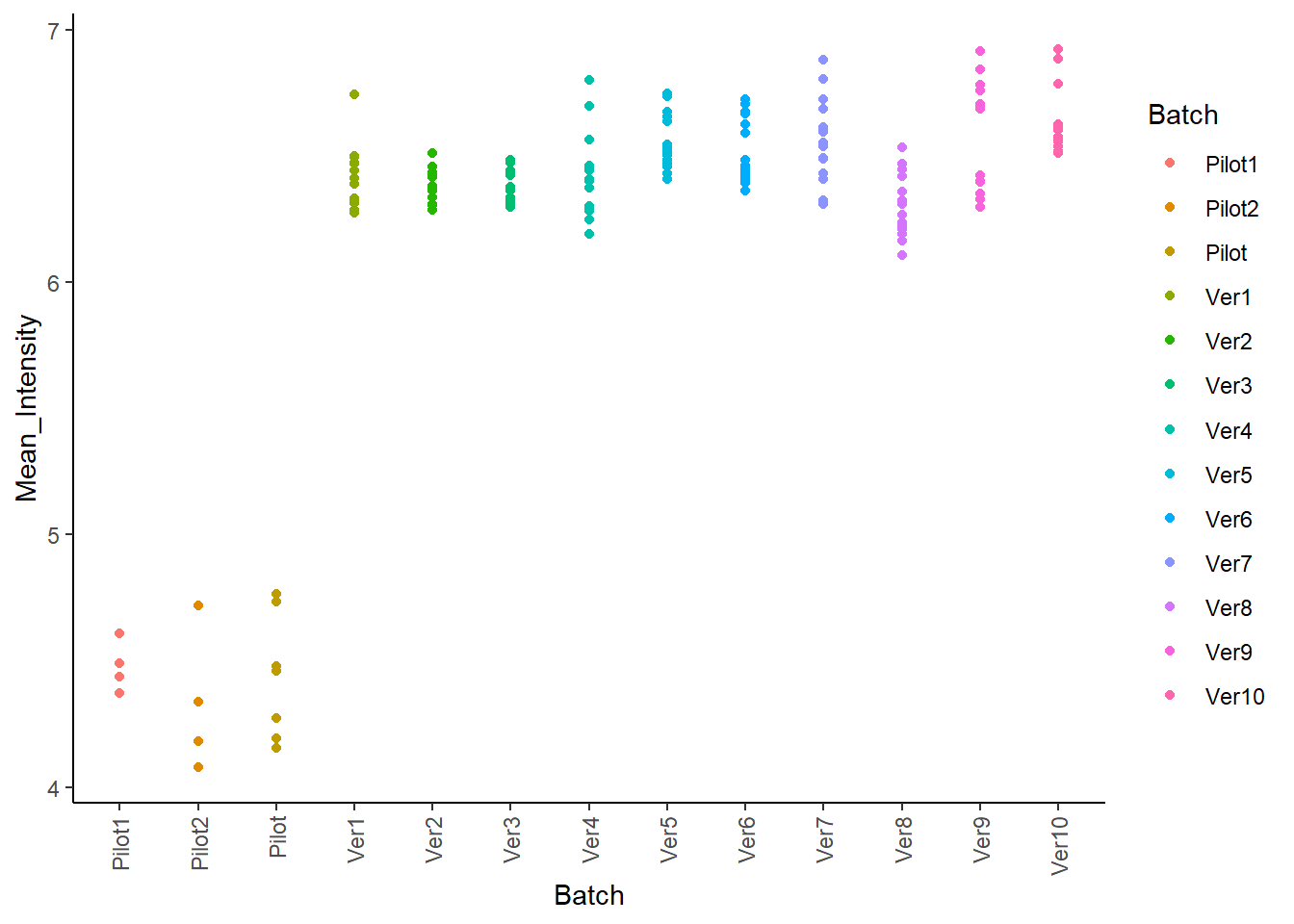


## Plotting the distribution of protein abundance for each batch

plot_boxplot(log_transformed_long, sample_annotation = annotation, sample_id_col = "FullRunName",

batch_col = "Batch", order_col = "Batch", color_scheme = "brewer")

**Figure 2: Protein abundance (y axis) categorised by batch (y axis).**

The figure demonstrates that there is a systematic difference in the protein abundances of patient samples analysed in the pilot (Pilot) as compared to the verification (Ver) study.


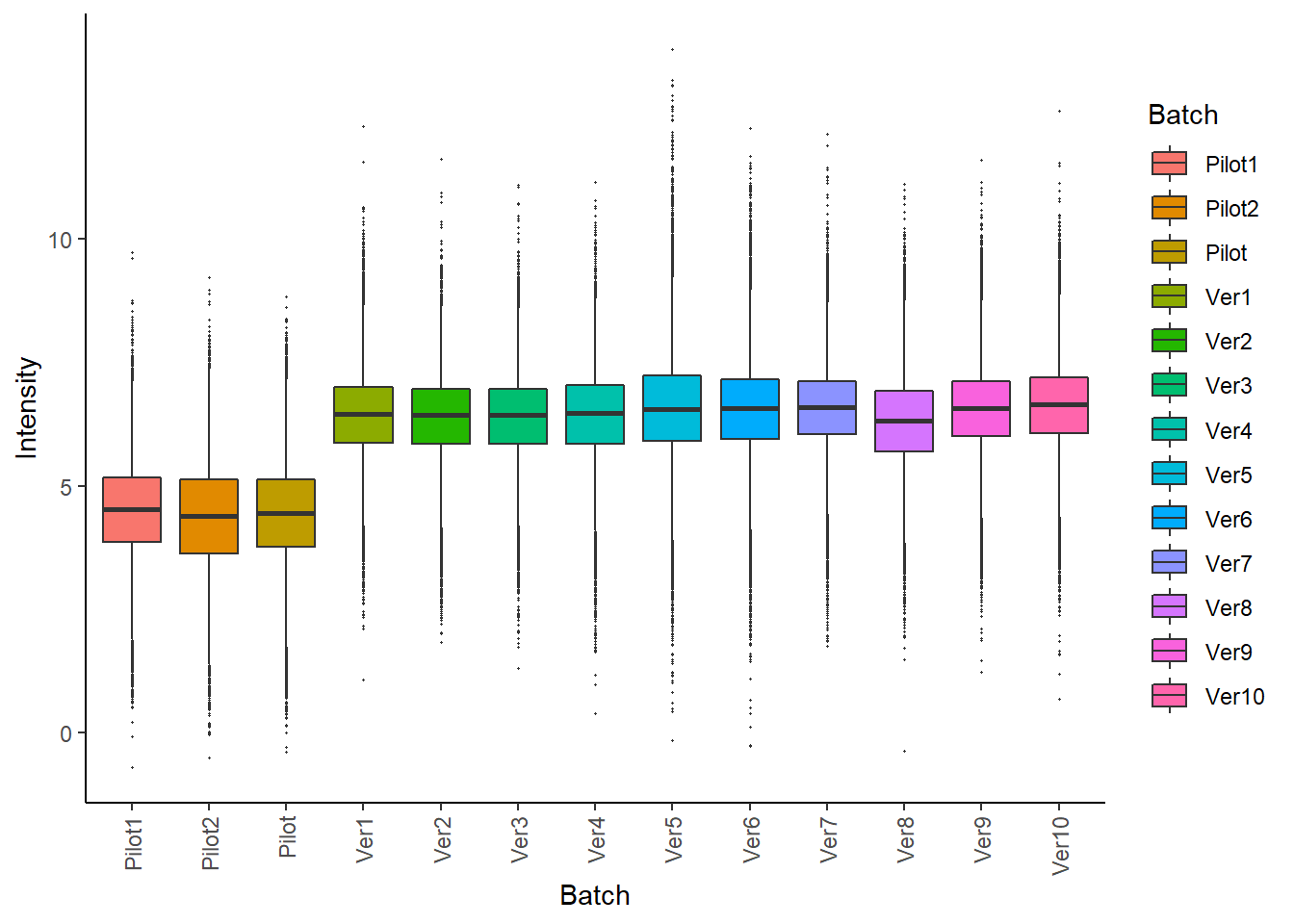


## Plotting the distribution of protein abundance for each biological replicates

plot_boxplot(log_transformed_long, sample_annotation = annotation, sample_id_col = "FullRunName",

batch_col = "Batch", color_by_batch = TRUE, filename = "Batch effects and correction/PD_raw_samples_boxplot.tiff",

ylimits = c(0, 20), width = 320, height = 100, color_scheme = "brewer")

**Figure 3: Protein abundance (y axis) categorised by patient samples (y axis).**

The figure demonstrates that there is a systematic difference in the protein abundances of patient samples analysed in the pilot (Pilot) as compared to the verification (Ver) study.


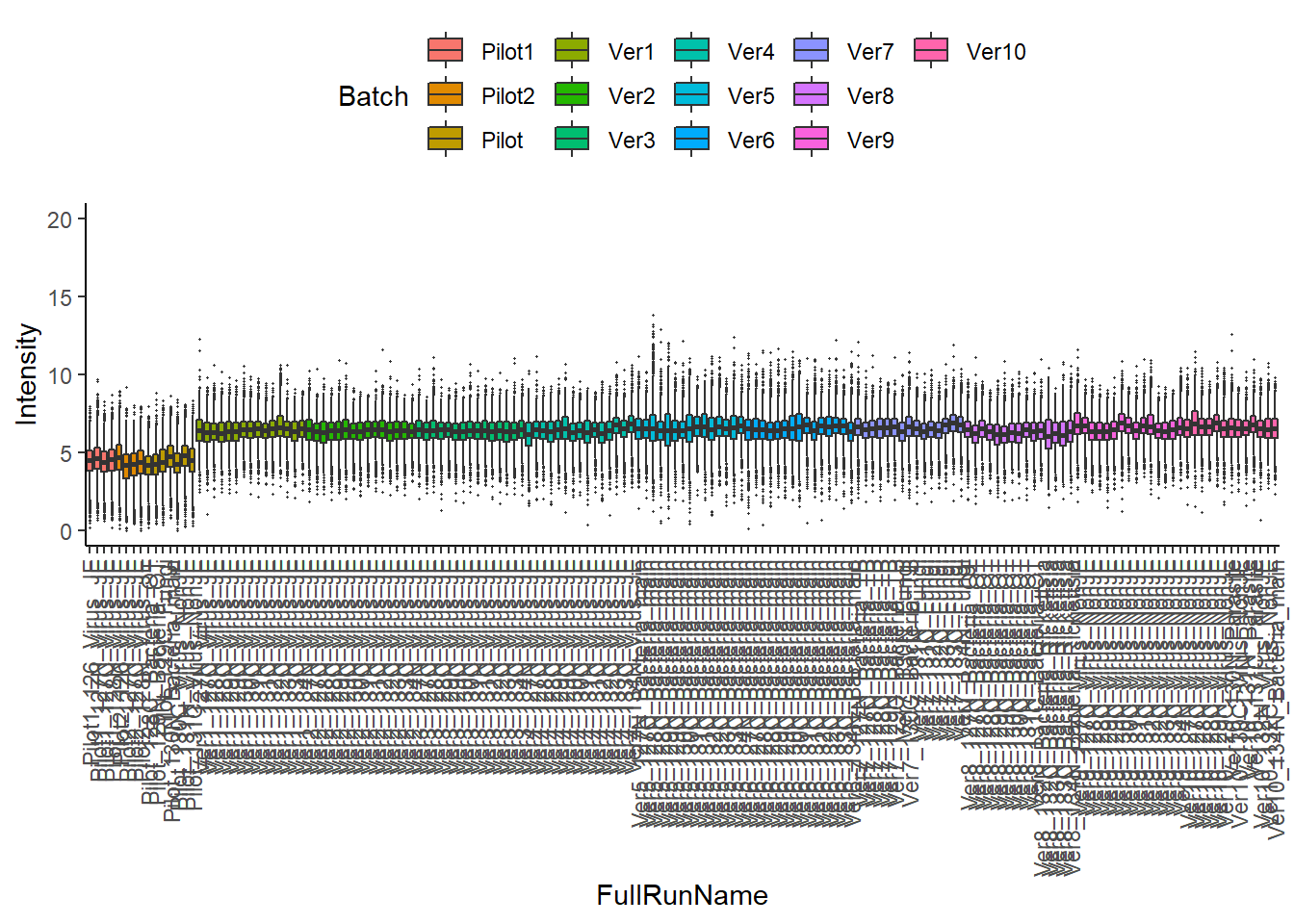


## Density plot

plotDensities(log2(log_transformed_df), group = annotation$Batch, col = c("red",

"green", "black", "pink", "yellow", "purple", "orange", "grey", "brown", "blue"),

legend = TRUE)

**Figure 4: Density plot illustrating the distribution of protein abundances (x axis) across patient samples in batches (colour coded).**

The figure demonstrates that there is a systematic difference in the mean protein abundances of patient samples analysed in the pilot (Pilot) as compared to the verification (Ver) studies.


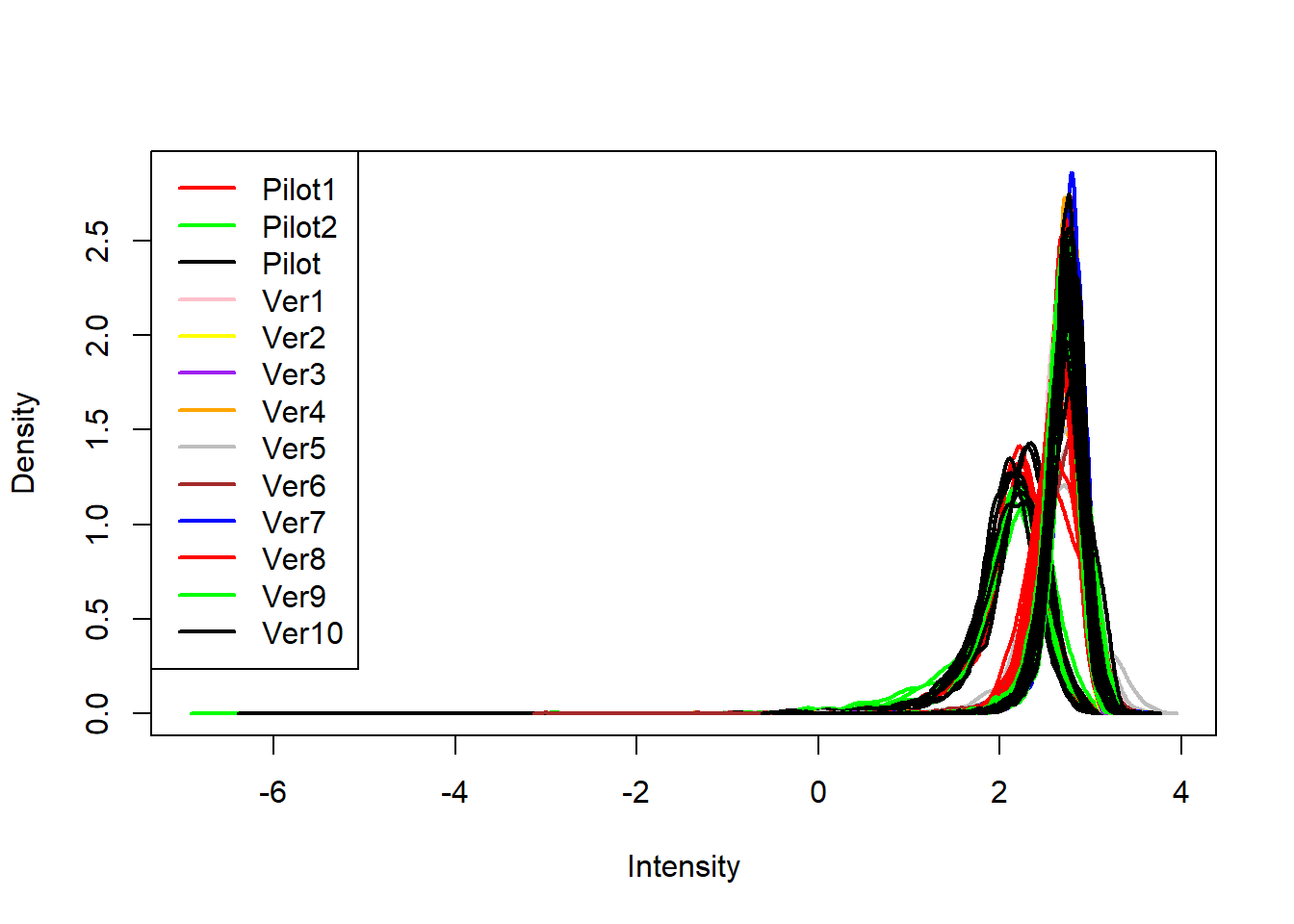


## Hierarchical clustering analysis

plot_hierarchical_clustering(log_transformed_df, annotation, factors_to_plot = c("Batch",

"Dataset", "JEV_nonJEV", "Channel", "Bio_rep"), sample_id_col = "FullRunName",

distance = "euclidean", agglomeration = "ward.D2", label_samples = FALSE)

**Figure 5: Hierarchical clustering of the protein expression of patient samples using Euclidean distancing and the Ward.D2 method.**

The figure demonstrates that there is a systematic difference in the protein expression of patient samples analysed in the pilot (Pilot) as compared to the verification (Ver) studies, while the same is not seen for batch or TMT channel. There is some biological variation, between JE and non-JE patient samples, however the true biological variation may be masked by the differences between the datasets.


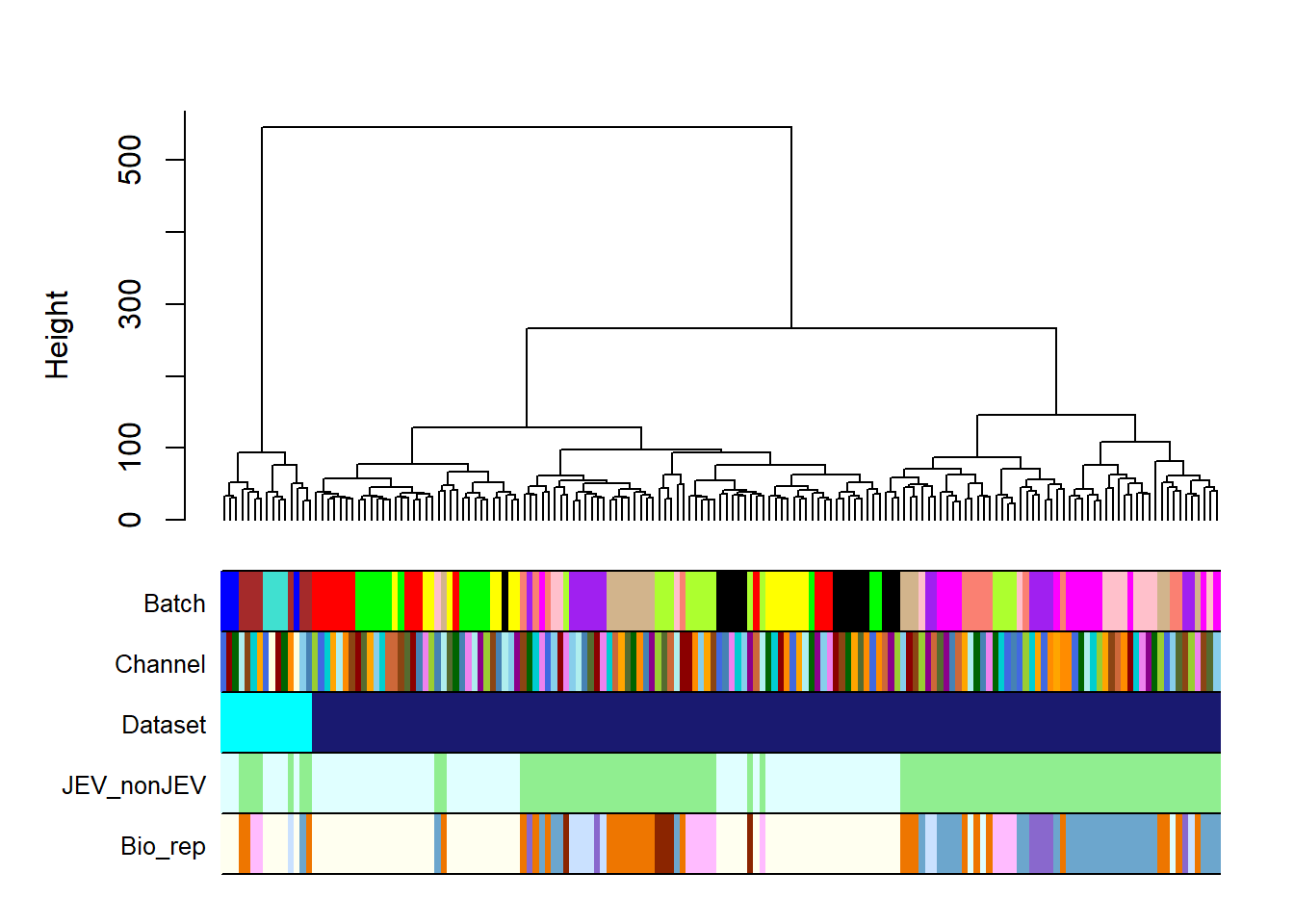


## Heatmap

plot_heatmap_diagnostic(log_transformed_df, annotation, factors_to_plot = c("Batch",

"Dataset", "JEV_nonJEV"), sample_id_col = "FullRunName", cluster_cols = TRUE,

show_rownames = FALSE, show_colnames = FALSE)

**Figure 6: Heatmap of the protein expression of patient samples.**

The figure emphasises previous findings, that there is systematic differences in the protein expression of patient samples analysed in the pilot (Pilot) as compared to the verification (Ver) studies, while the same is not seen for batch. There is some biological variation, between JE and non-JE patient samples, however the true biological variation may be masked by the differences between the datasets.


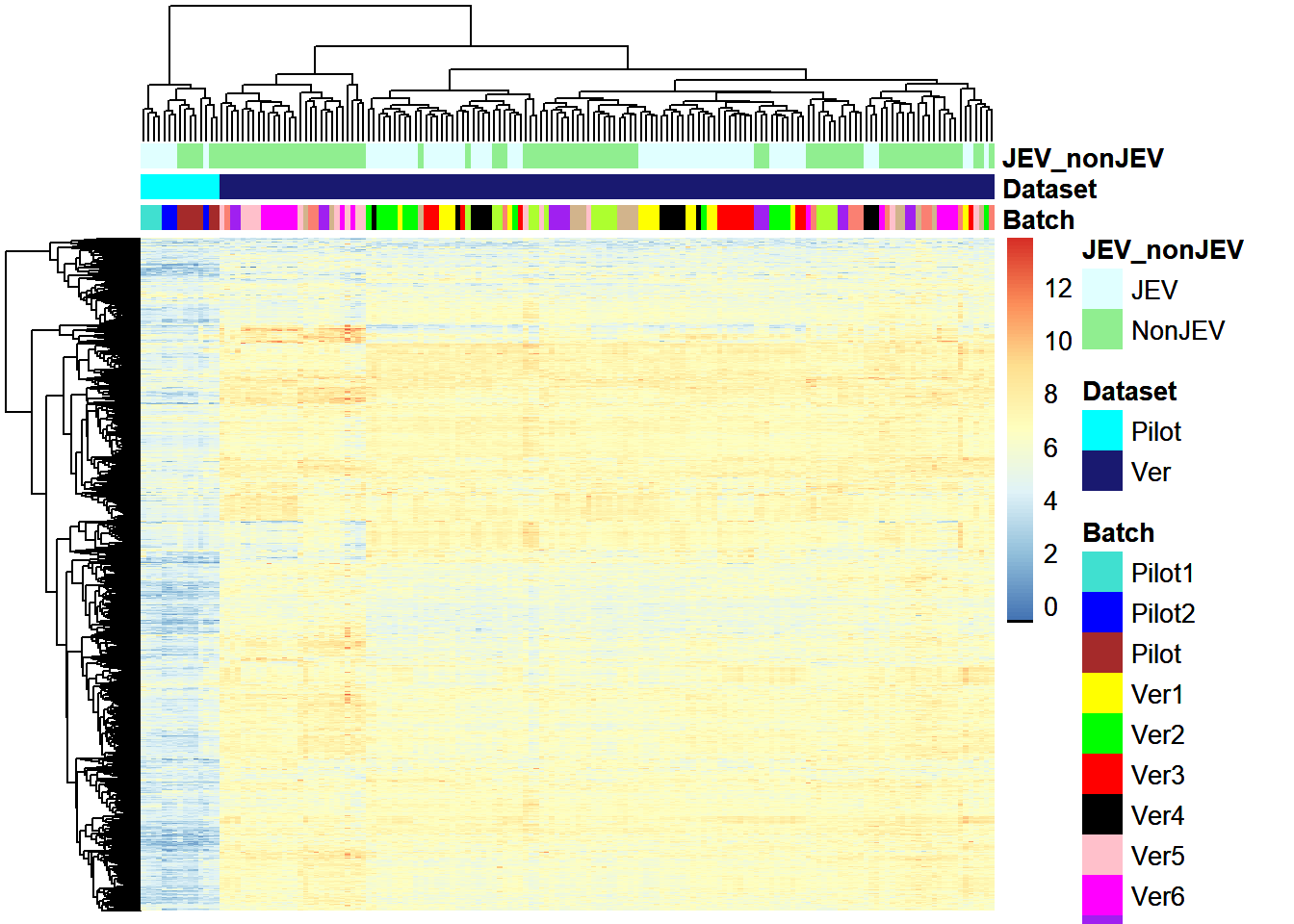


## Principle component analysis (PCA) plot

plot_PCA(log_transformed_df, annotation, color_by = "Batch", plot_title = "Batch")

**Figure 7a: Principle component analysis (PCA) of the protein expression in patient samples (individual dots) across different batches (different colours).**

There is systematic difference between the protein expression of patient samples in the pilot as compared to the verification study.


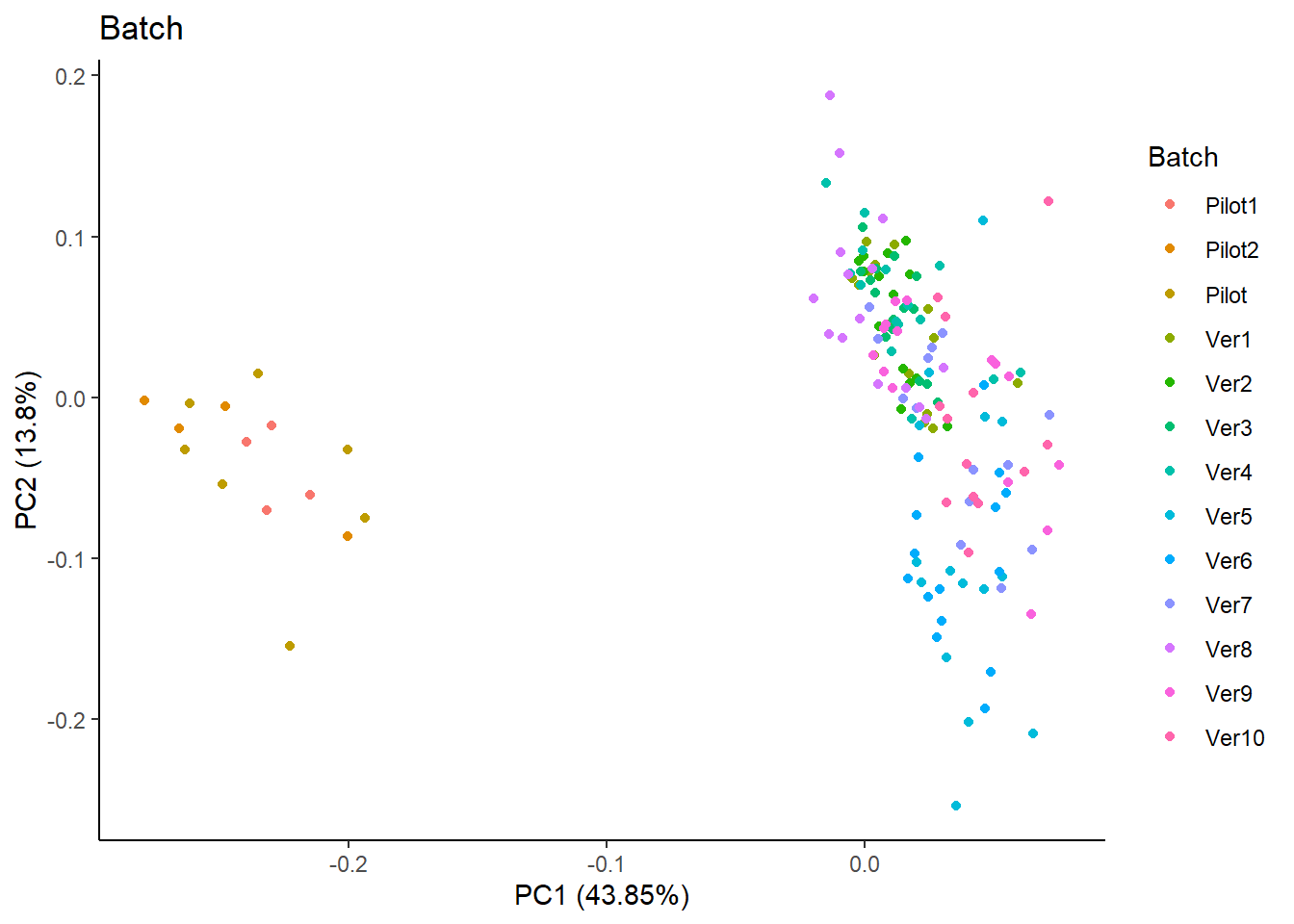


plot_PCA(log_transformed_df, annotation, color_by = "JEV_nonJEV", plot_title = "JEV_nonJEV")

**Figure 7b: Principle component analysis (PCA) of the protein expression in patient samples (individual dots) in JE (red) and non-JE infections (blue).**

There is systematic difference between the protein expression of patient samples in the pilot as compared to the verification study, and there is a risk that this masks any true biological differences.


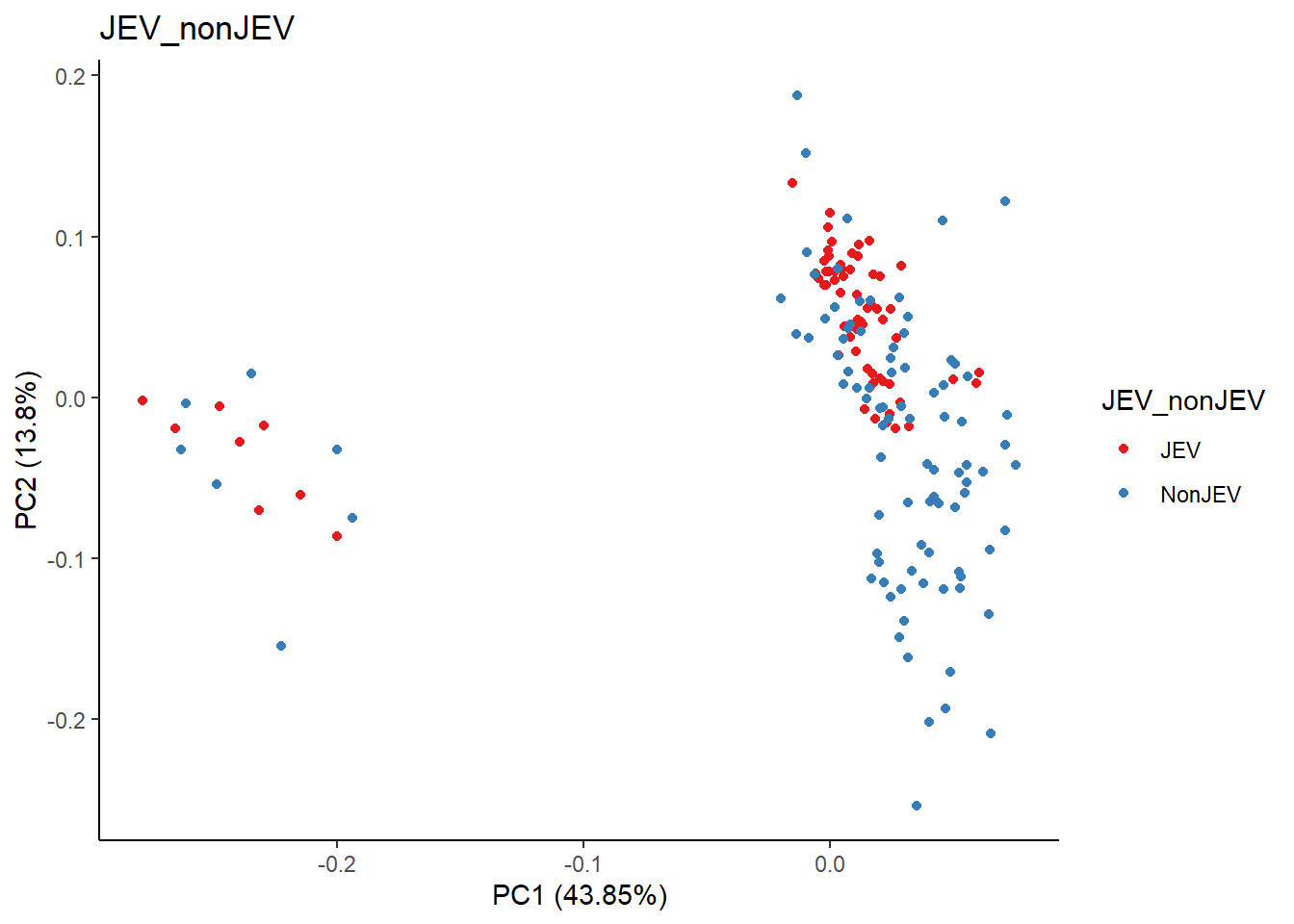


## Principle component variance analysis (PCVA) plot

plot_PVCA(log_transformed_df, annotation, technical_factors = c("Dataset"), biological_factors = c("JEV_nonJEV",

"Bio_rep"))

**Figure 8: Principle component variance analysis of protein expression of patient samples.**

Overall, differences between protein expression between the datasets outweighs any true biological differences.


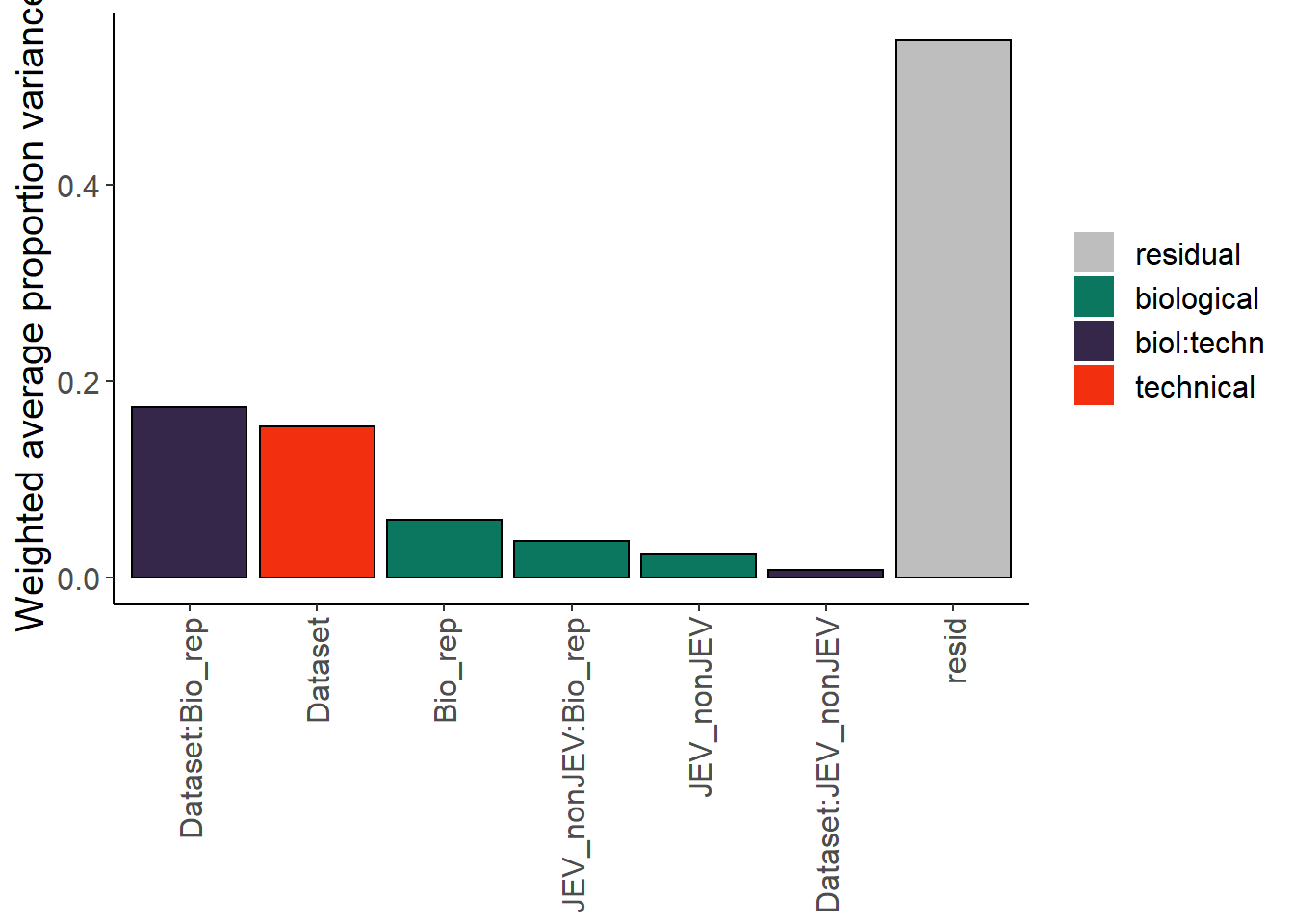


# B) Analysis of corrected data (normalised with RobNorm and then corrected for batch effects with sva ComBat)

data_postcorrection = as.data.frame(read_excel("data_jitter_combined_post_RobNorm and ComBat.xlsx"))

table(rowSums(is.na(data_postcorrection)))

##

## 0

## 2176

row.names(data_postcorrection) = data_postcorrection$Protein

data_postcorrection$Protein = NULL

Import the metadata

metadata = read_excel("C:/Users/tehmi/Dropbox/Documents/PhD/Proteomics/TMT_Verification/Data analysis/Variables/Combined_patient_data.xlsx")

Creating an annotation file that aligns with the data and processing the data file to the correct format (colnames as samples, rownames as feature IDS, log2transformed)

metadata$jev[metadata$jev == 1] = "JEV"

metadata$jev[metadata$jev == 0] = "NonJEV"

metadata$Dataset[metadata$Dataset == 1] = "Pilot"

metadata$Dataset[metadata$Dataset == 2] = "Ver"

metadata$names = names(data_postcorrection)

names = names(data_postcorrection)

metadata = unite(metadata, "metadata", c("names", "Dataset", "jev", "category of infection"),

sep = ":")

names = metadata$metadata

names(data_postcorrection) = names

annotation = data_postcorrection

annotation = pivot_longer(data_postcorrection, c(1:163), names_to = "FullRunName",

values_to = "Abundance")

annotation = tidyr::separate(annotation, FullRunName, c("Batch", "Replicate"), remove = FALSE,

sep = ";")

annotation = separate(annotation, Replicate, c("Channel", "Dataset", "JEV_nonJEV",

"Bio_rep"), remove = TRUE, sep = ":")

annotation = select(annotation, -c(7))

annotation = distinct(annotation, FullRunName, .keep_all = TRUE)

annotation$Batch = factor(annotation$Batch, levels = c("Pilot1", "Pilot2", "Pilot",

"Ver1", "Ver2", "Ver3", "Ver4", "Ver5", "Ver6", "Ver7", "Ver8", "Ver9", "Ver10"))

annotation$Channel = factor(annotation$Channel, levels = c("126", "127N", "127C",

"128N", "128C", "129N", "129C", "130N", "130C", "131N", "131C", "132N", "132C",

"133N", "133C", "134N"))

annotation$Dataset = as.factor(annotation$Dataset)

annotation$JEV_nonJEV = as.factor(annotation$JEV_nonJEV)

annotation$Bio_rep = as.factor(annotation$Bio_rep)

annotation = unite(annotation, "FullRunName", c("Batch", "Channel", "Bio_rep"), remove = FALSE)

names(data_postcorrection) = annotation$FullRunName

Saving a dataframe in a wide format and long format

log_transformed_df = data_postcorrection

log_transformed_long = matrix_to_long(log_transformed_df)

## Plotting the mean protein abundance for patient samples in each batch

plot_sample_mean(log_transformed_df, annotation, sample_id_col = "FullRunName", batch_col = "Batch",

color_by_batch = TRUE, order_col = "Batch", color_scheme = "brewer")

**Figure 9: Mean protein abundance of patient samples (y axis) categorised by batch (y axis).**

The previously seen systematic differences between datasets has been removed.


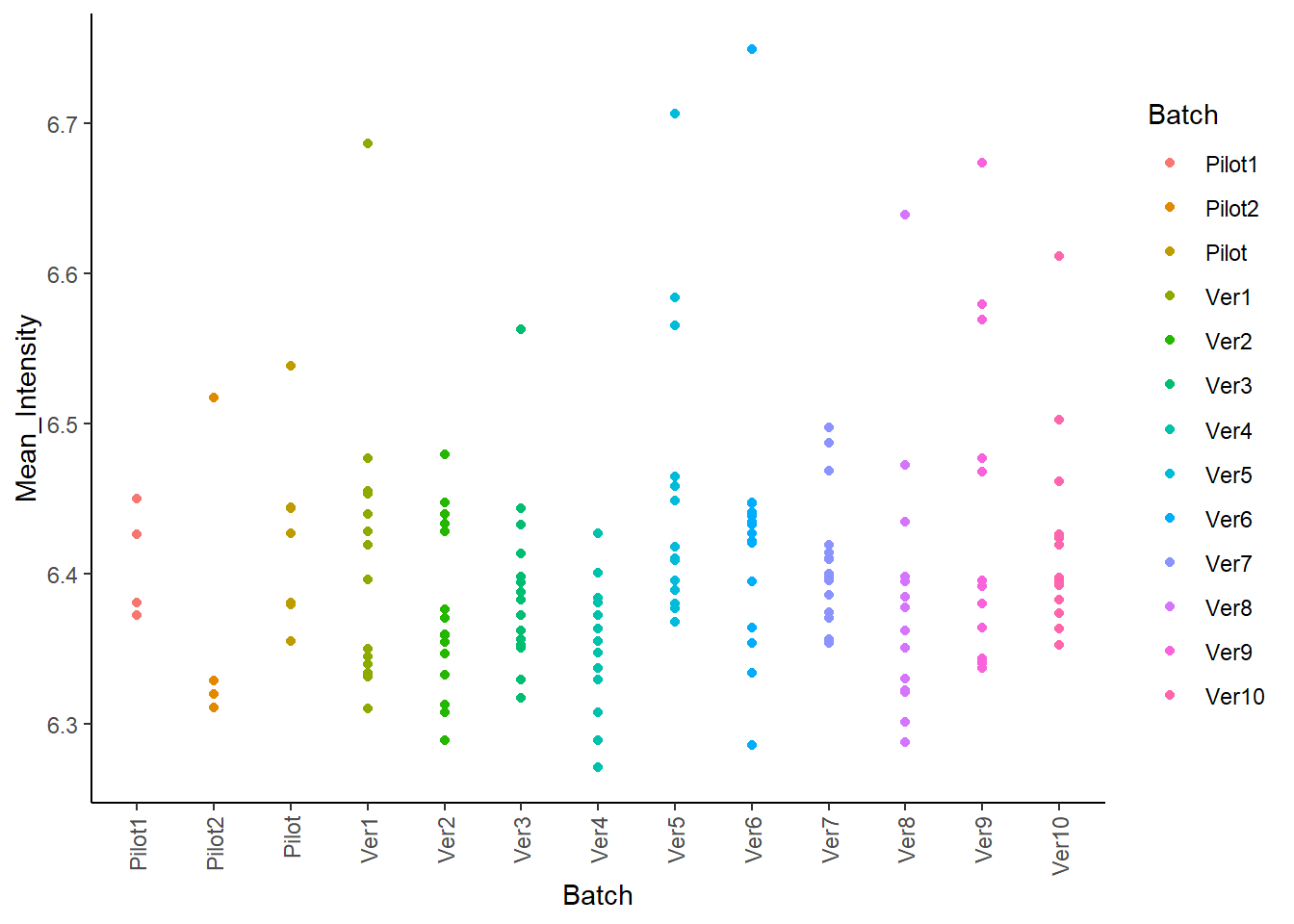


## Plotting the distribution of protein abundance for each batch

plot_boxplot(log_transformed_long, sample_annotation = annotation, sample_id_col = "FullRunName",

batch_col = "Batch", order_col = "Batch", color_scheme = "brewer")

**Figure 10: Protein abundance (y axis) categorised by batch (y axis).**

The previously seen systematic differences between datasets has been removed.


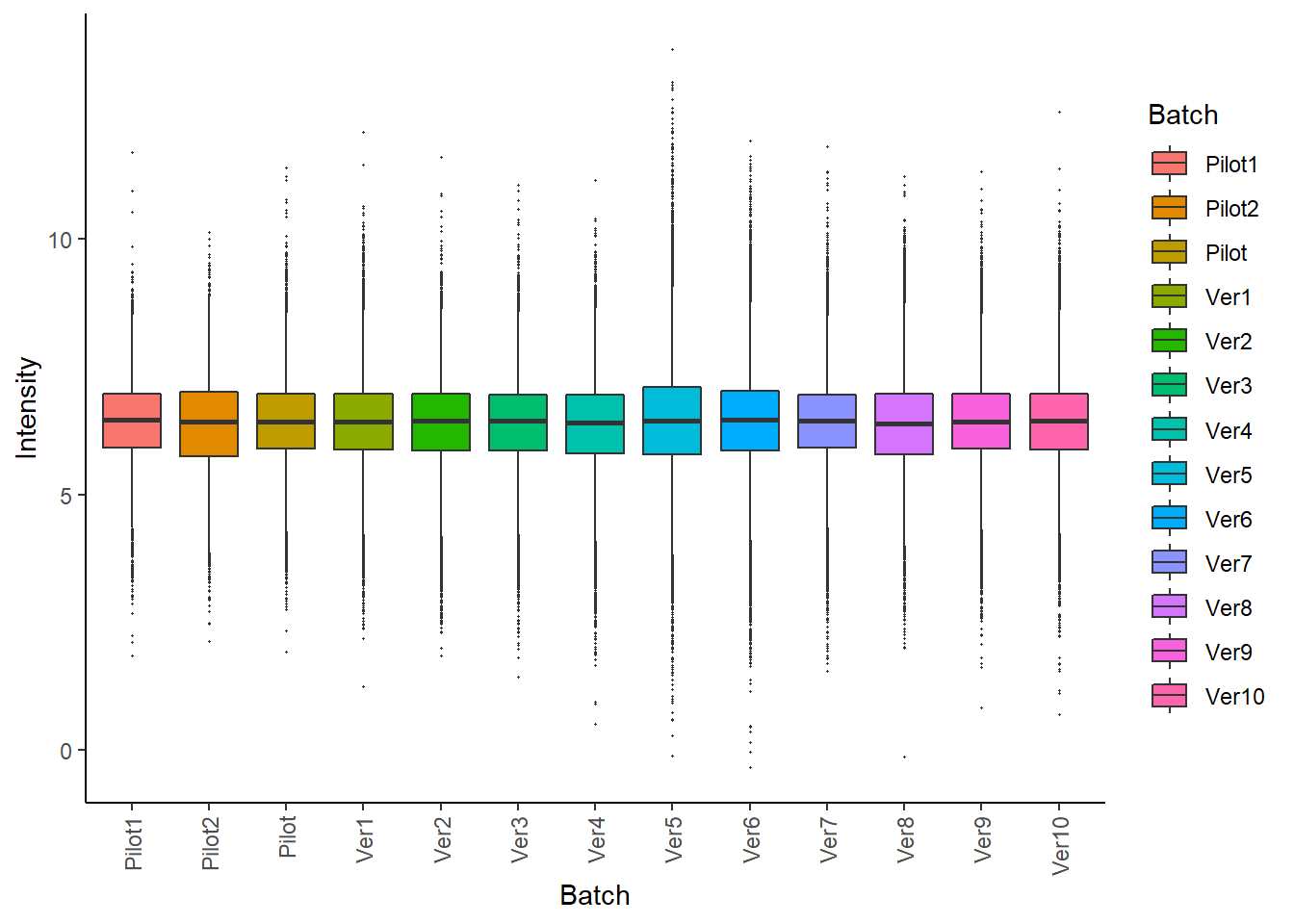


## Plotting the distribution of protein abundance for each biological replicates

plot_boxplot(log_transformed_long, sample_annotation = annotation, sample_id_col = "FullRunName",

batch_col = "Batch", color_by_batch = TRUE, filename = "Batch effects and correction/PD_raw_samples_boxplot.tiff",

ylimits = c(0, 20), width = 320, height = 100, color_scheme = "brewer")

**Figure 11: Protein abundance (y axis) categorised by patient samples (y axis).**

The previously seen systematic differences between datasets has been removed.


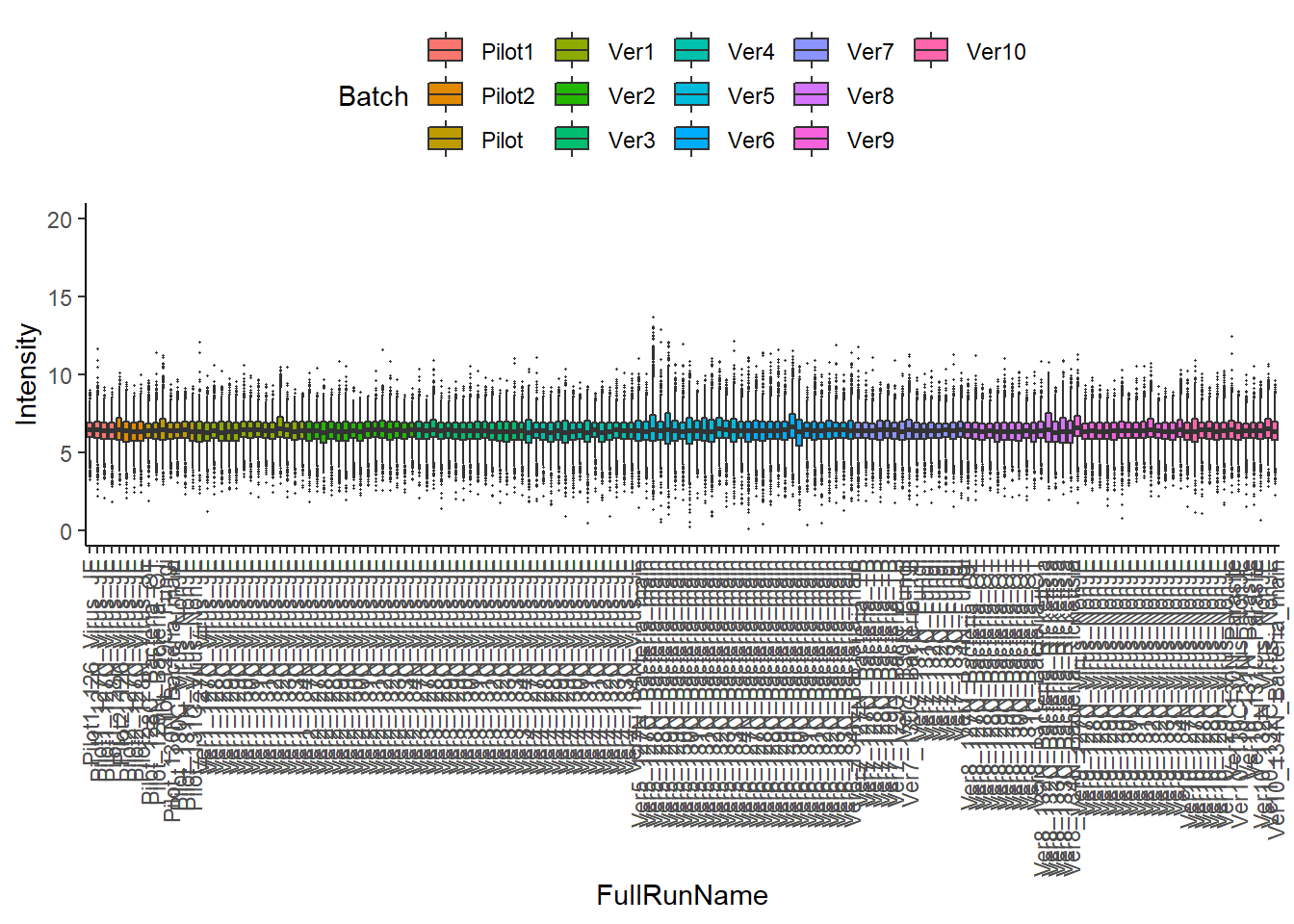


## Density plot

plotDensities(log2(log_transformed_df), group = annotation$Batch, col = c("red",

"green", "black", "pink", "yellow", "purple", "orange", "grey", "brown", "blue"),

legend = TRUE)

**Figure 12: Density plot illustrating the distribution of protein abundances (x axis) across patient samples in batches (colour coded).**

The previously seen systematic differences between datasets has been removed.


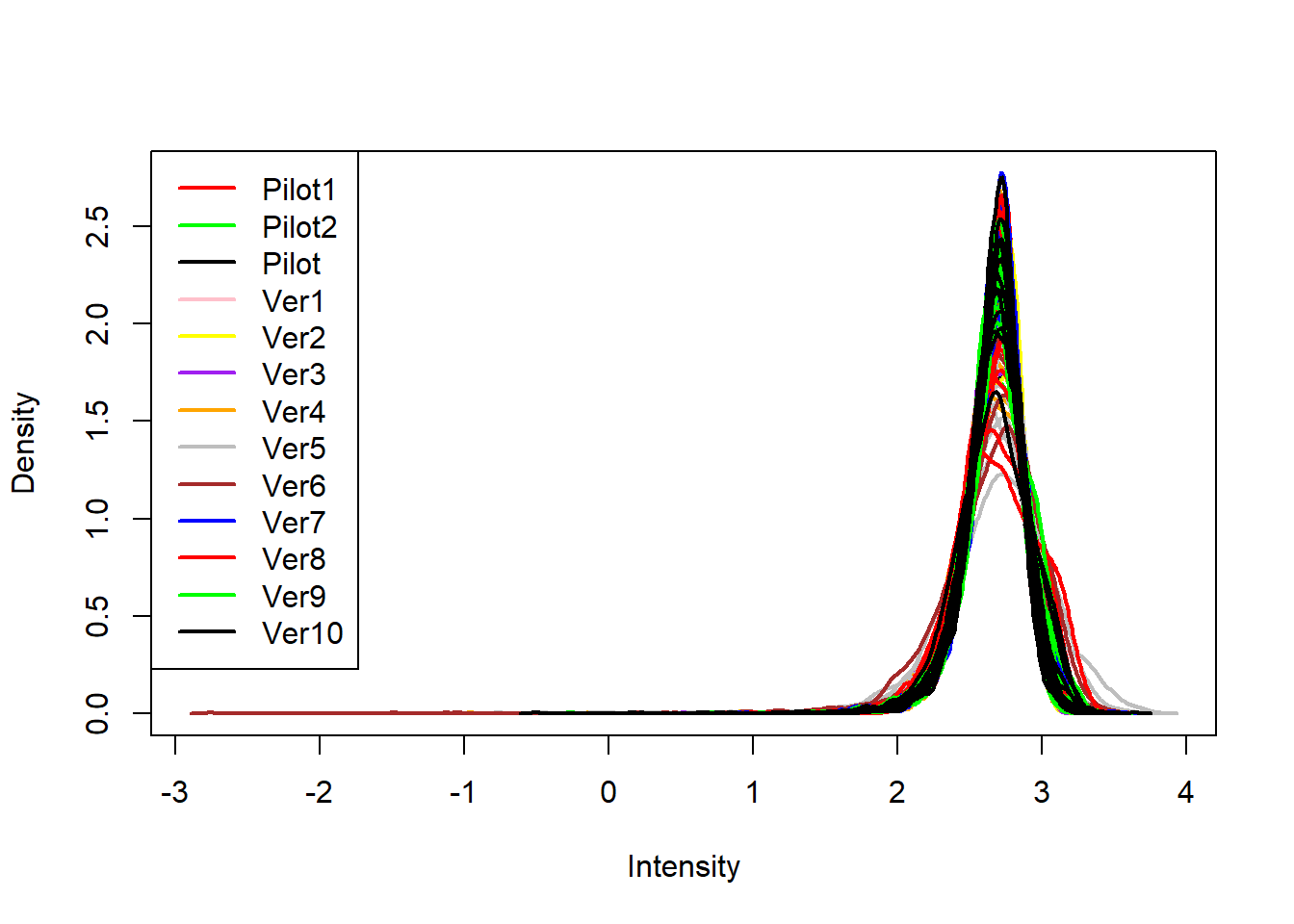


## Hierarchical clustering analysis

plot_hierarchical_clustering(log_transformed_df, annotation, factors_to_plot = c("Batch",

"Dataset", "JEV_nonJEV", "Channel", "Bio_rep"), sample_id_col = "FullRunName",

distance = "euclidean", agglomeration = "ward.D2", label_samples = FALSE)

**Figure 13: Hierarchical clustering of the protein expression of patient samples using Euclidean distancing and the Ward.D2 method.**

The previously seen systematic differences between datasets has been removed.


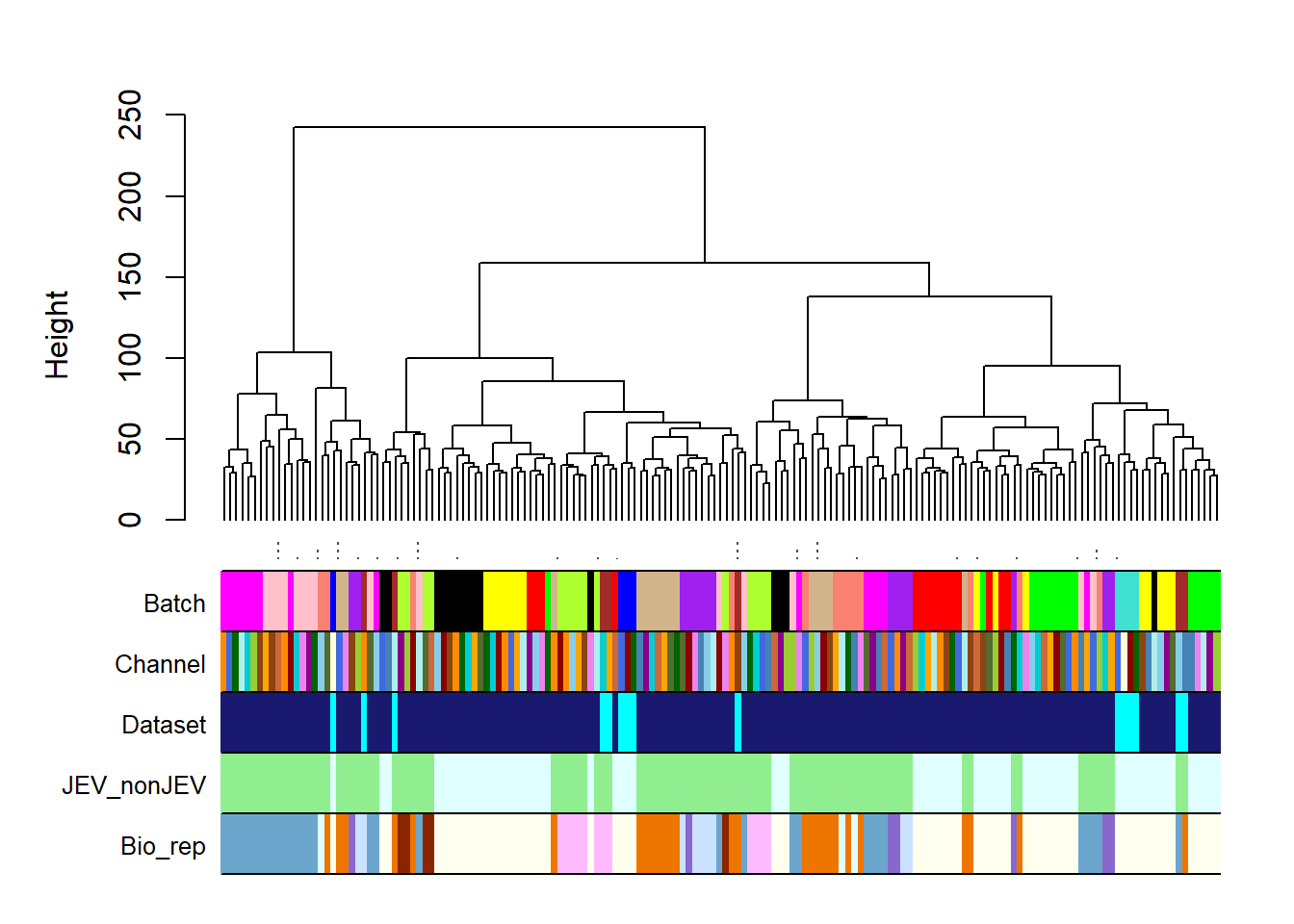


## Heatmap

plot_heatmap_diagnostic(log_transformed_df, annotation, factors_to_plot = c("Batch",

"Dataset", "JEV_nonJEV"), sample_id_col = "FullRunName", cluster_cols = TRUE,

show_rownames = FALSE, show_colnames = FALSE)

**Figure 14: Heatmap of the protein expression of patient samples.**

The previously seen systematic differences between datasets has been removed.


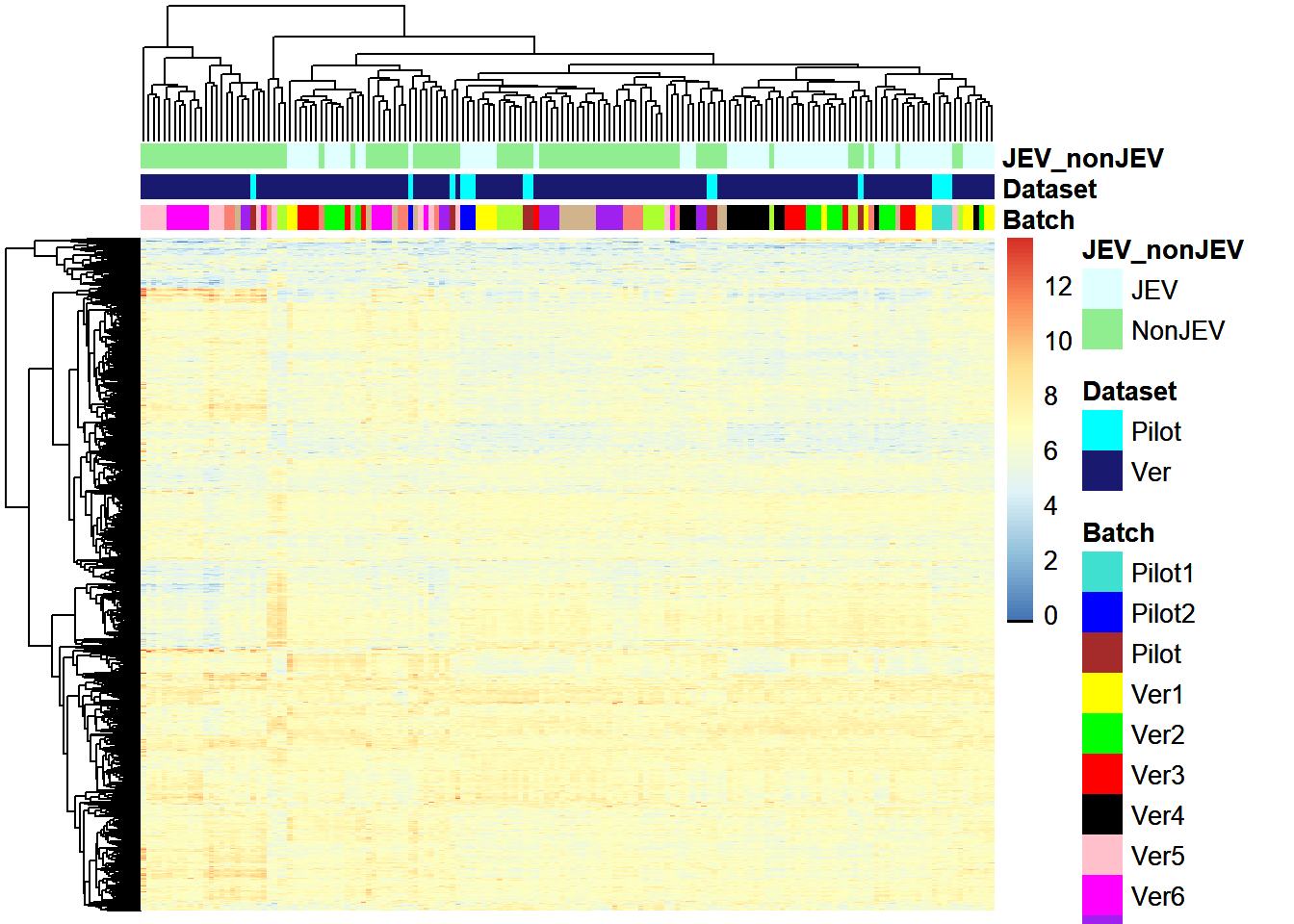


## Principle component analysis (PCA) plot

plot_PCA(log_transformed_df, annotation, color_by = "Batch", plot_title = "Batch")

**Figure 15a: Principle component analysis (PCA) of the protein expression in patient samples (individual dots) across different batches (different colours).**

The previously seen systematic differences between datasets has been removed.


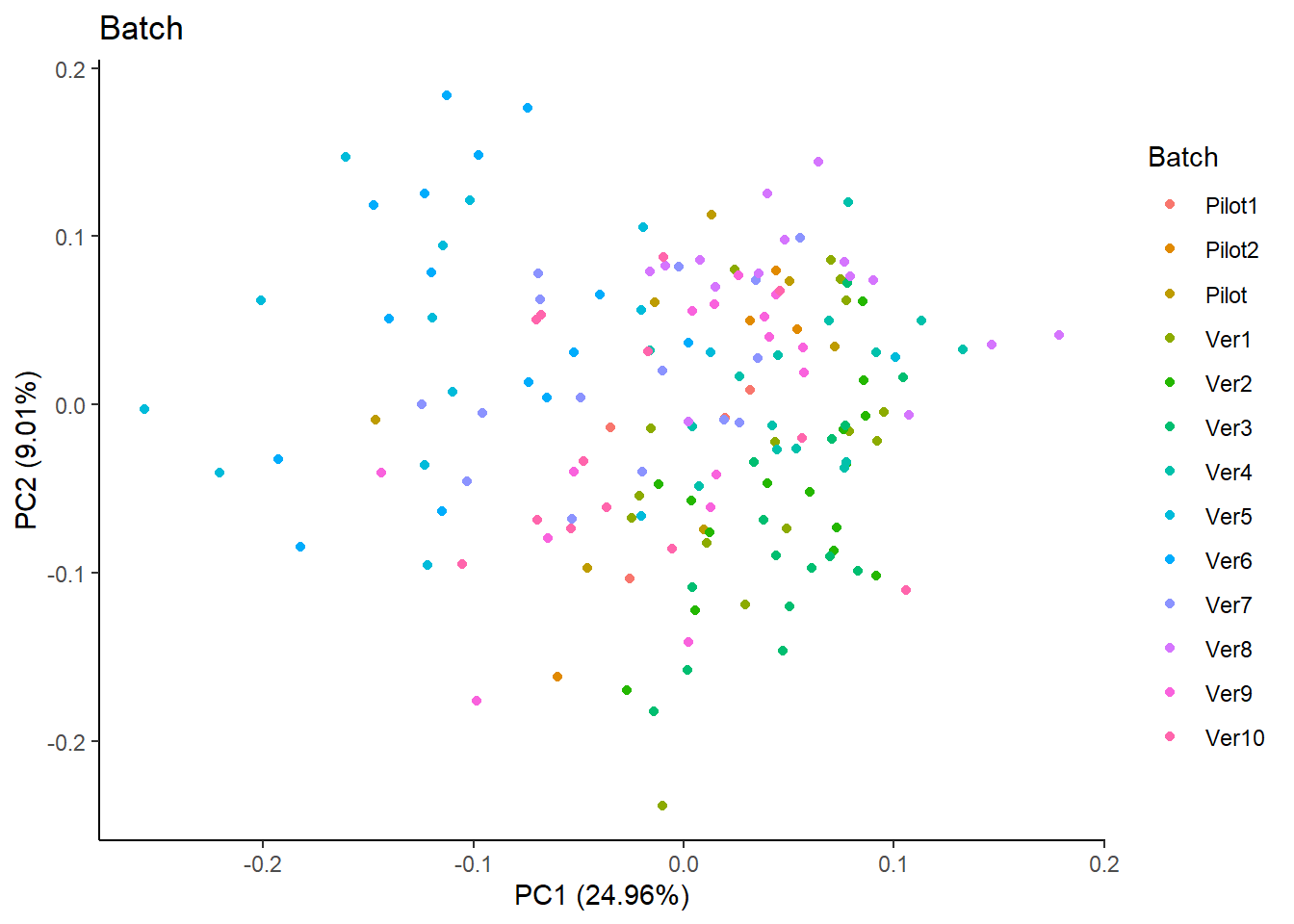


plot_PCA(log_transformed_df, annotation, color_by = "JEV_nonJEV", plot_title = "JEV_nonJEV")

**Figure 15b: Principle component analysis (PCA) of the protein expression in patient samples (individual dots) in JE (red) and non-JE infections (blue).**

The plot illustrates that there is variation between protein expression of patient samples with JE as compared to non-JE infections.


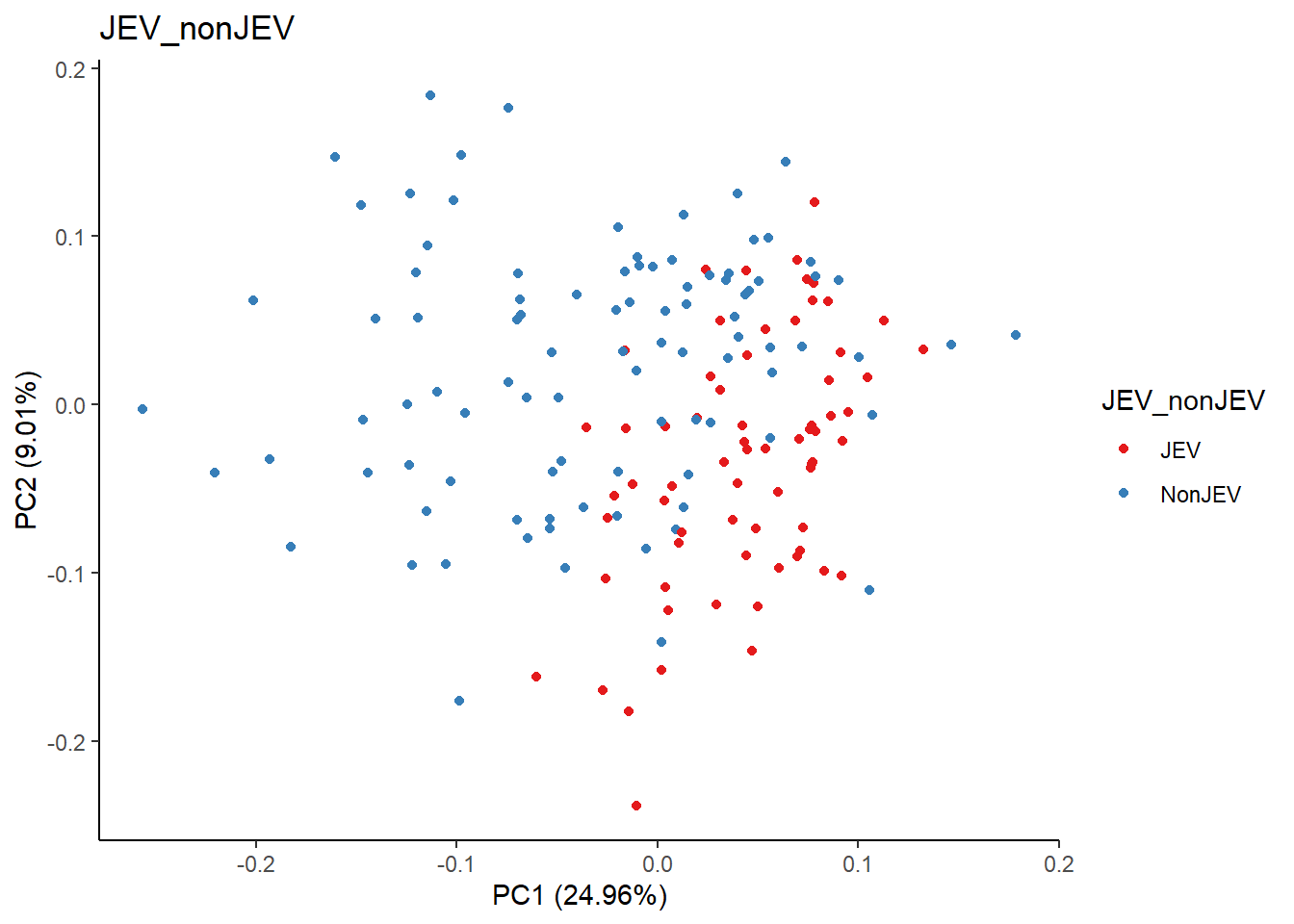


## Principle component variance analysis (PCVA) plot

plot_PVCA(log_transformed_df, annotation, technical_factors = c("Dataset"), biological_factors = c("JEV_nonJEV",

"Bio_rep"))

**Figure 16: Principle component variance analysis of protein expression of patient samples.**

The technical variation of protein expression of patient samples has been reduced to less than 1%, allowing the unmasking of true biological variation.


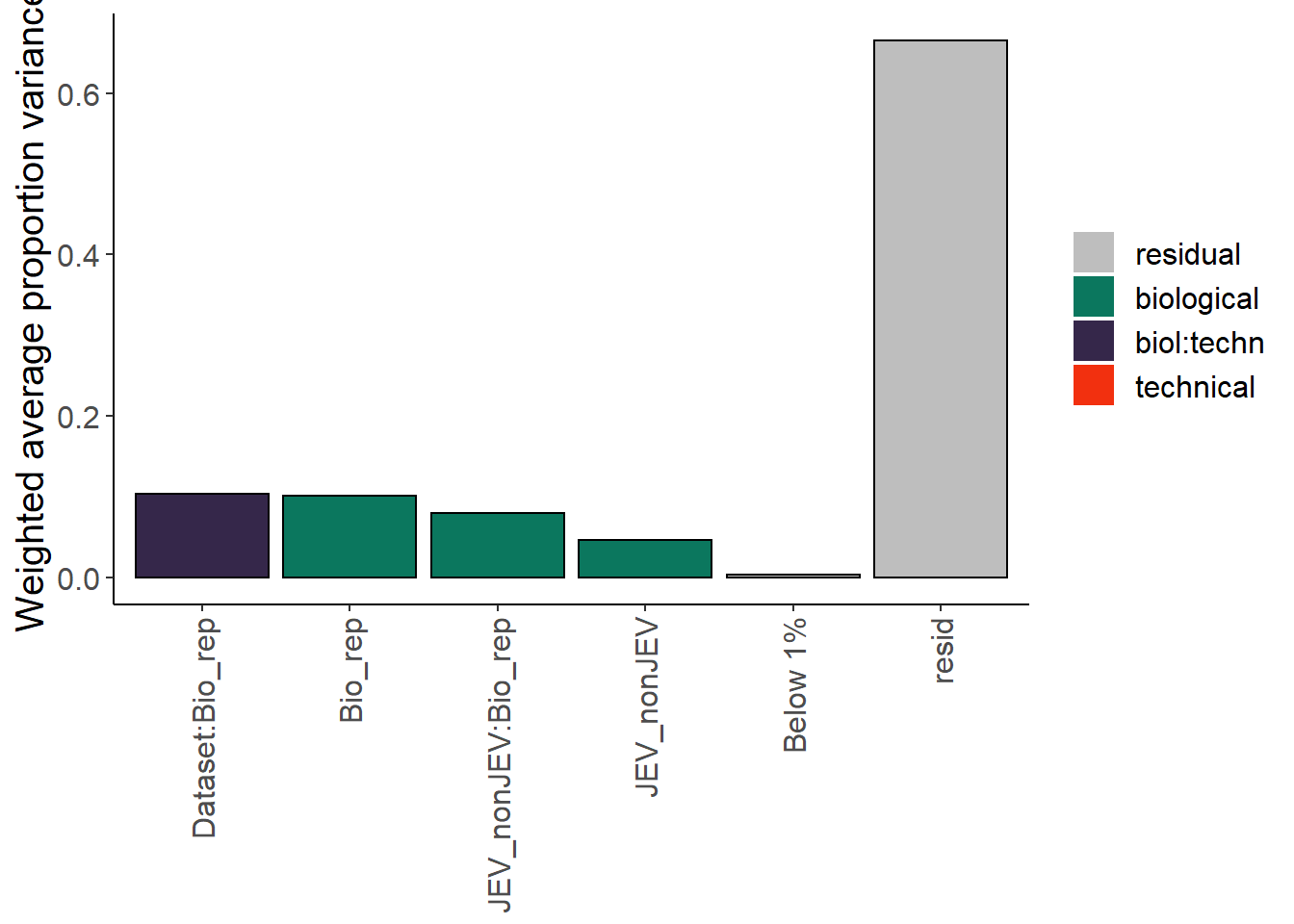


dev.off()

## null device

## 1

**Figure 17: Correlation heatmap of LC-MS data from 16 samples (JE=5, non-JE=11) processed by TMT and DIA methods* produced using MetaboAnalyst.**


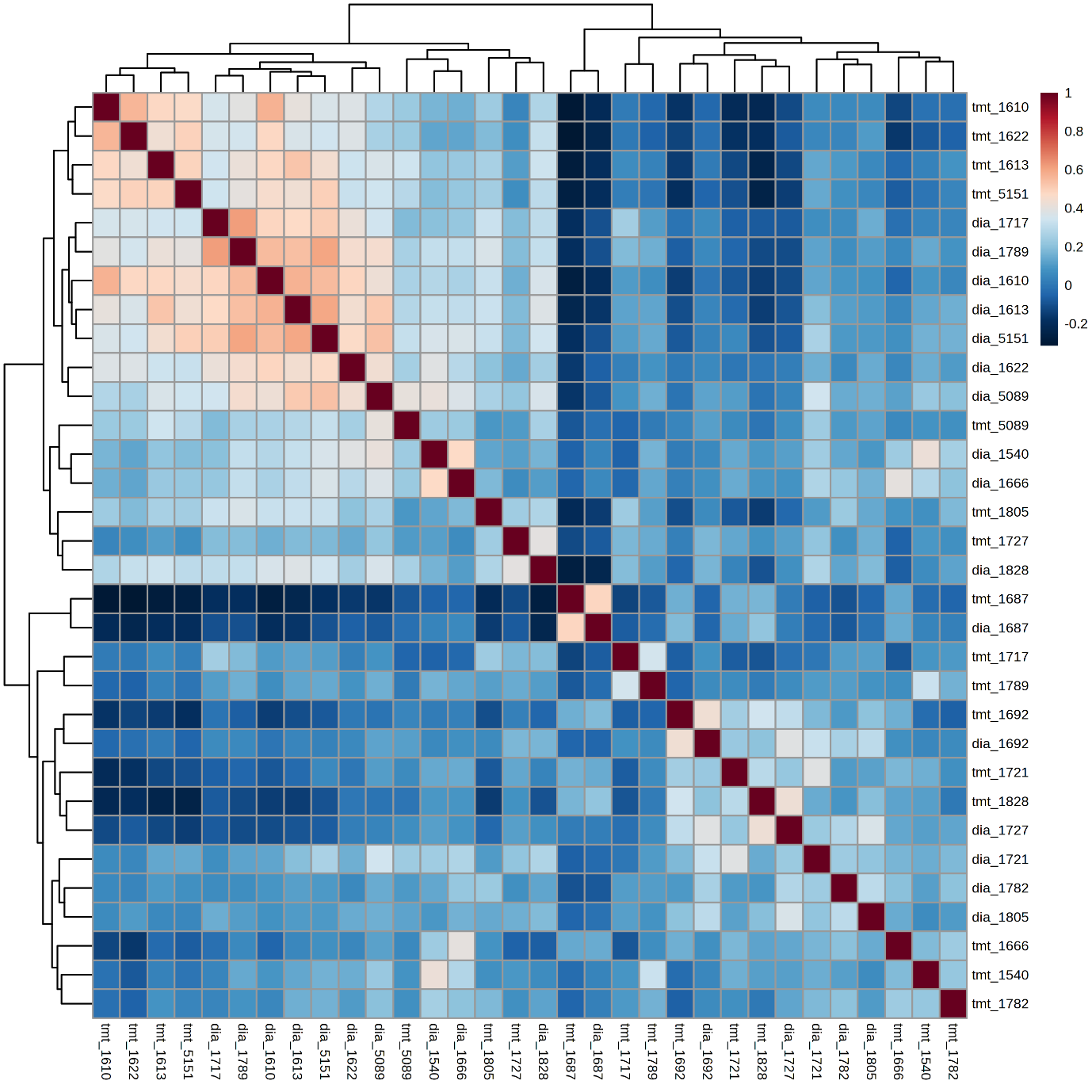


*JE samples = 1610, 1613, 1622, 5151 and 5089.
