# Supplementary material for: Deep Proteomics Network and Machine Learning Analysis of Human Cerebrospinal Fluid in Japanese Encephalitis Virus Infection
Source: J Proteome Res. 2023 May 23;22(6):1614–29. doi: 10.1021/acs.jproteome.2c00563 (PMC10246887; doi:10.1021/acs.jproteome.2c00563)

S8: JE vs. non-JE protein set enrichment analysis: Results of protein set enrichment analysis performed using the WebGestalt online tool

Figure 1: Summary of results for biological process (red), cellular component (blue) and molecular function (green) categories


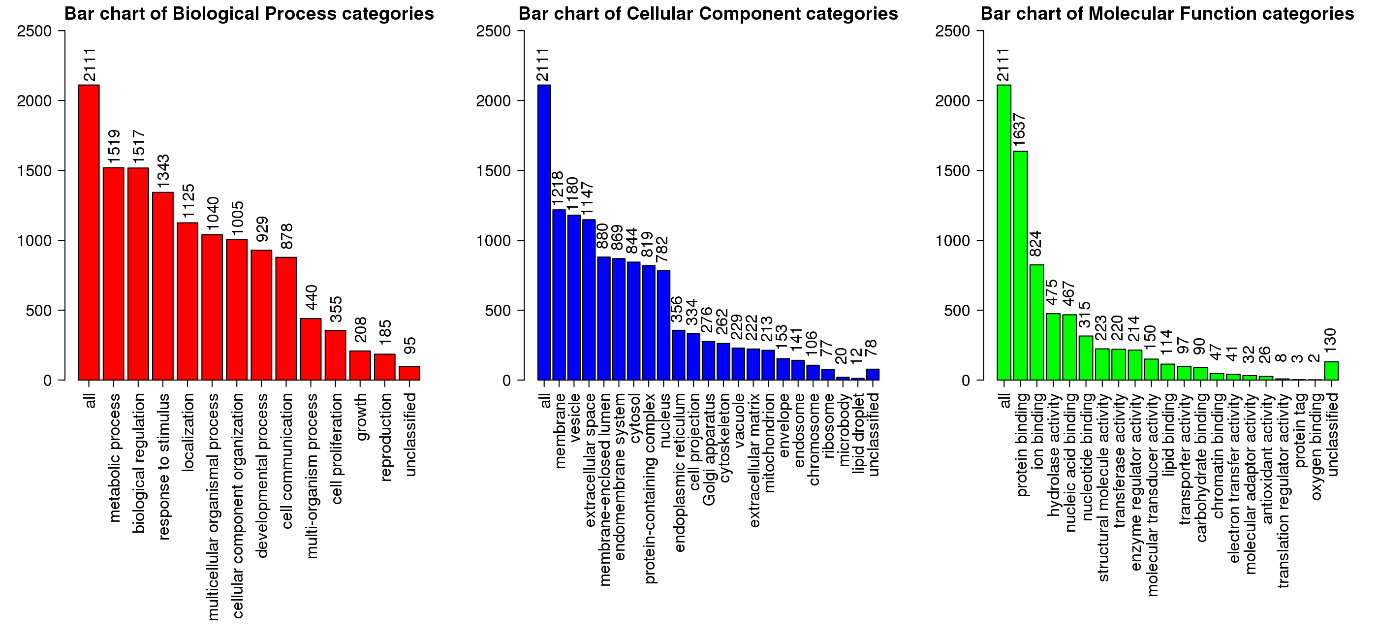


Table 1: Protein set enrichment results using WebGestalt and gene set enrichment analysis (GSEA)

| Gene set | Description | Size | Leading edge  number | Enrichment  score | Normalised  enrichment score | P value | FDR |
| --- | --- | --- | --- | --- | --- | --- | --- |
| GO:0090559 | regulation of membrane permeability | 20 | 8 | 0.62269 | 2.6492 | <2.2e-16 | <2.2e-16 |
| GO:0070972 | protein localization to endoplasmic reticulum | 73 | 57 | 0.41535 | 2.5592 | <2.2e-16 | 0.000452 |
| GO:0031345 | negative regulation of cell projection organization | 58 | 31 | 0.43136 | 2.5193 | <2.2e-16 | 0.000603 |
| GO:0090150 | establishment of protein localization to membrane | 103 | 54 | 0.37351 | 2.4649 | <2.2e-16 | 0.001808 |
| GO:0051961 | negative regulation of nervous system development | 76 | 44 | 0.39456 | 2.4312 | <2.2e-16 | 0.002531 |
| GO:0051156 | glucose 6-phosphate metabolic process | 13 | 11 | -0.55836 | -2.6216 | <2.2e-16 | 0.003206 |
| GO:0010721 | negative regulation of cell development | 86 | 48 | 0.37508 | 2.3705 | <2.2e-16 | 0.004067 |
| GO:0010975 | regulation of neuron projection development | 108 | 60 | 0.3562 | 2.3451 | <2.2e-16 | 0.004519 |
| GO:0006732 | coenzyme metabolic process | 86 | 63 | -0.22953 | -2.7121 | <2.2e-16 | 0.004809 |
| GO:0099177 | regulation of trans-synaptic signalling | 69 | 43 | 0.38908 | 2.3496 | <2.2e-16 | 0.004906 |
| GO:0006413 | translational initiation | 89 | 49 | 0.35313 | 2.287 | <2.2e-16 | 0.007029 |
| GO:0021700 | developmental maturation | 55 | 38 | 0.38412 | 2.2422 | <2.2e-16 | 0.009761 |
| GO:0006605 | protein targeting | 120 | 62 | 0.3277 | 2.2143 | <2.2e-16 | 0.011092 |
| GO:0050808 | synapse organization | 104 | 60 | 0.32806 | 2.1727 | <2.2e-16 | 0.013696 |
| GO:0010821 | regulation of mitochondrion organization | 38 | 13 | 0.4264 | 2.181 | <2.2e-16 | 0.013707 |
| GO:0072524 | pyridine-containing compound metabolic process | 58 | 43 | -0.24724 | -2.4019 | <2.2e-16 | 0.017233 |
| GO:0006401 | RNA catabolic process | 112 | 54 | 0.32018 | 2.1393 | <2.2e-16 | 0.018015 |
| GO:0048638 | regulation of developmental growth | 73 | 38 | 0.35038 | 2.1414 | <2.2e-16 | 0.018915 |
| GO:0034502 | protein localization to chromosome | 16 | 16 | -0.47828 | -2.4233 | 0.005495 | 0.02084 |
| GO:0007626 | locomotory behaviour | 38 | 26 | 0.40371 | 2.1159 | <2.2e-16 | 0.022029 |
| GO:0061564 | axon development | 136 | 72 | 0.30344 | 2.0962 | <2.2e-16 | 0.025306 |
| GO:0046683 | response to organophosphorus | 20 | 15 | 0.48717 | 2.0789 | 0.001185 | 0.029071 |
| GO:0007006 | mitochondrial membrane organization | 27 | 17 | 0.42589 | 2.0266 | 0.004474 | 0.045712 |
| GO:0016072 | rRNA metabolic process | 36 | 30 | 0.39682 | 2.0155 | 0.001086 | 0.045791 |
| GO:0048857 | neural nucleus development | 12 | 6 | 0.57727 | 2.02 | 0.003841 | 0.046183 |
| GO:0070585 | protein localization to mitochondrion | 31 | 8 | 0.40898 | 1.9983 | 0.003315 | 0.047979 |
| GO:0008637 | apoptotic mitochondrial changes | 31 | 10 | 0.4141 | 2.0032 | 0.002235 | 0.048393 |

Figure 2: Bar chart of protein set enrichment results using WebGestalt and gene set enrichment analysis (GSEA)


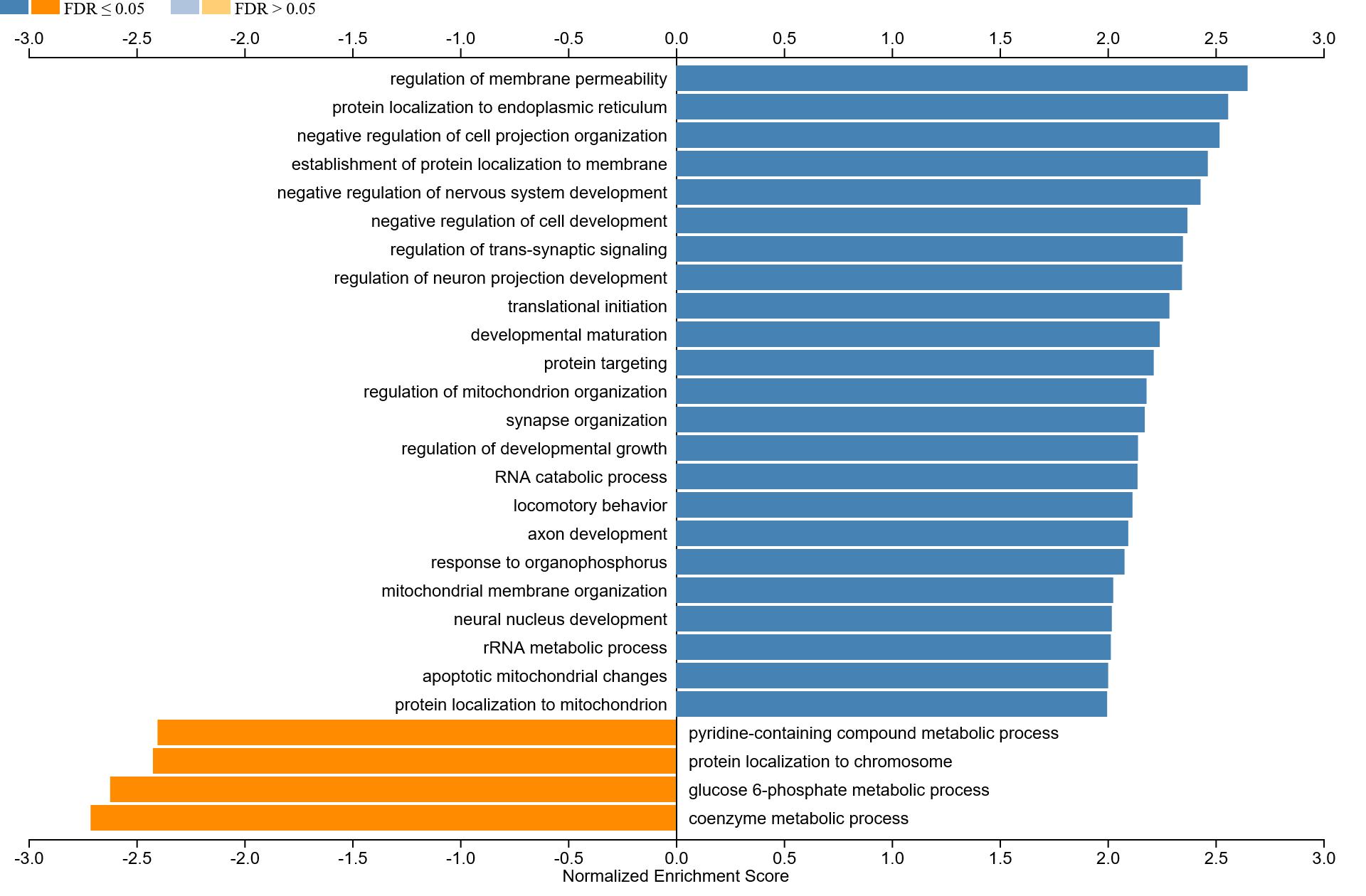


Figure 3: Directed acyclic graph (DAG) with redundancy reduction for weighted set coverage to reveal relationships among the terms obtained using protein set enrichment results using WebGestalt and gene set enrichment analysis (GSEA)
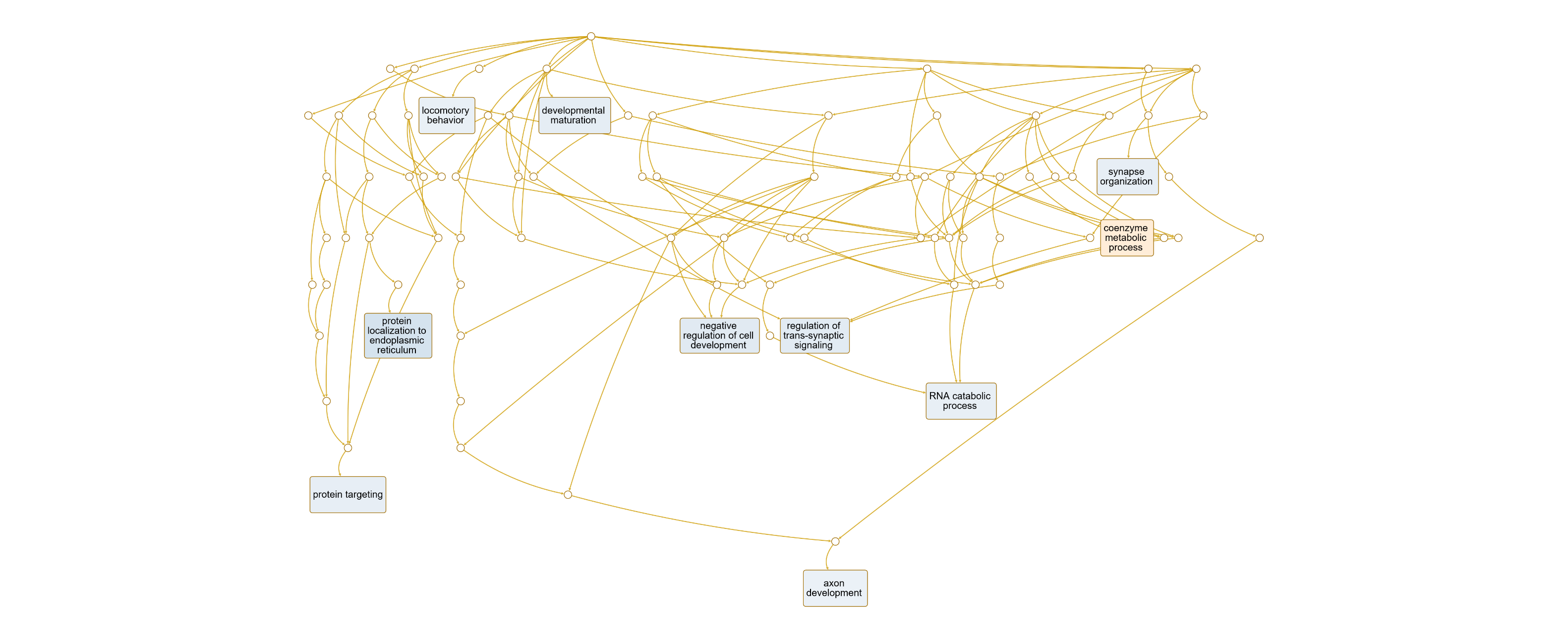

Supplement: Supplementary file 1 — pr2c00563_si_001.zip [file pr2c00563_si_001.zip › S8_JE vs. non-JE protein set enrichment analysis.docx]
