# Supplementary figures and images for: Deep Proteomics Network and Machine Learning Analysis of Human Cerebrospinal Fluid in Japanese Encephalitis Virus Infection
Source: J Proteome Res. 2023 May 23;22(6):1614–29. doi: 10.1021/acs.jproteome.2c00563 (PMC10246887; doi:10.1021/acs.jproteome.2c00563)

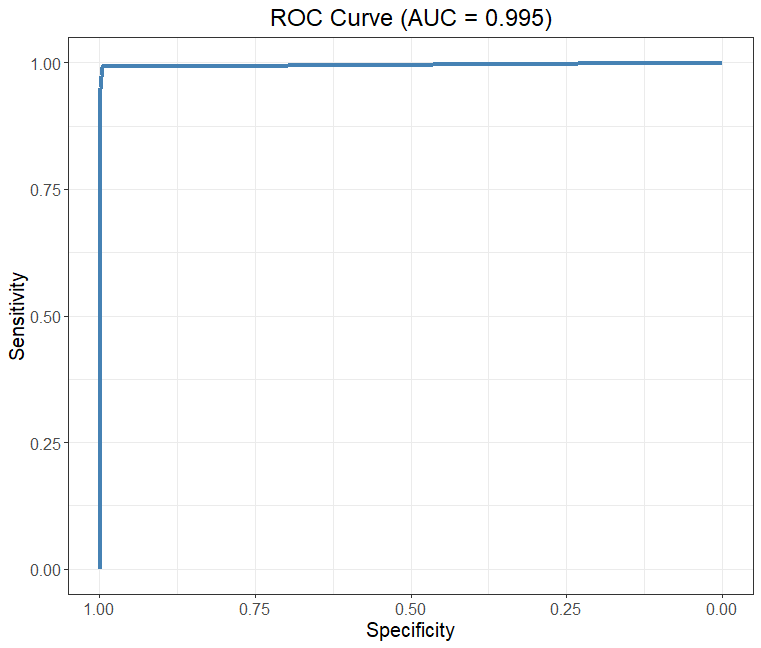

Supplement: Supplementary file 1 — pr2c00563_si_001.zip [file pr2c00563_si_001.zip › S14_ROC.tif]

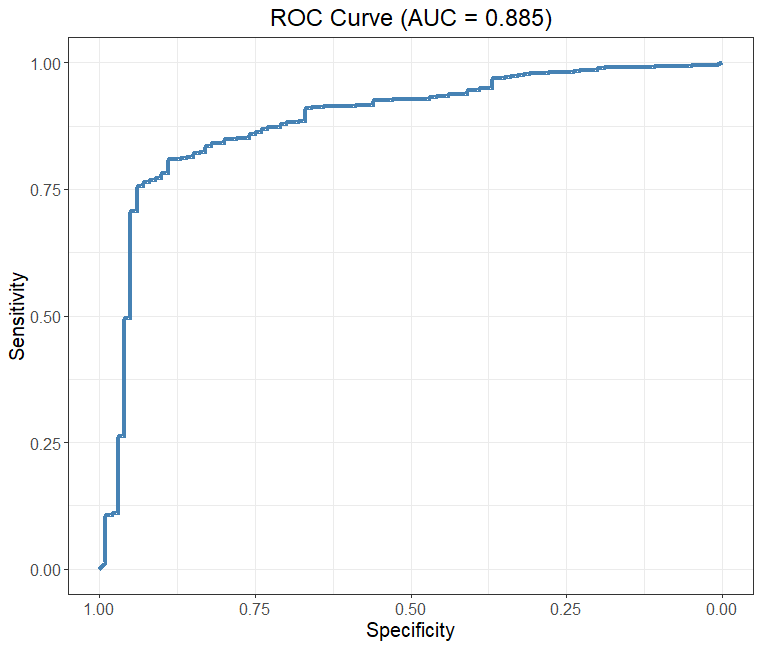

Supplement: Supplementary file 1 — pr2c00563_si_001.zip [file pr2c00563_si_001.zip › S16_ROC outcome.tif]

## Sample clustering to detect outliers

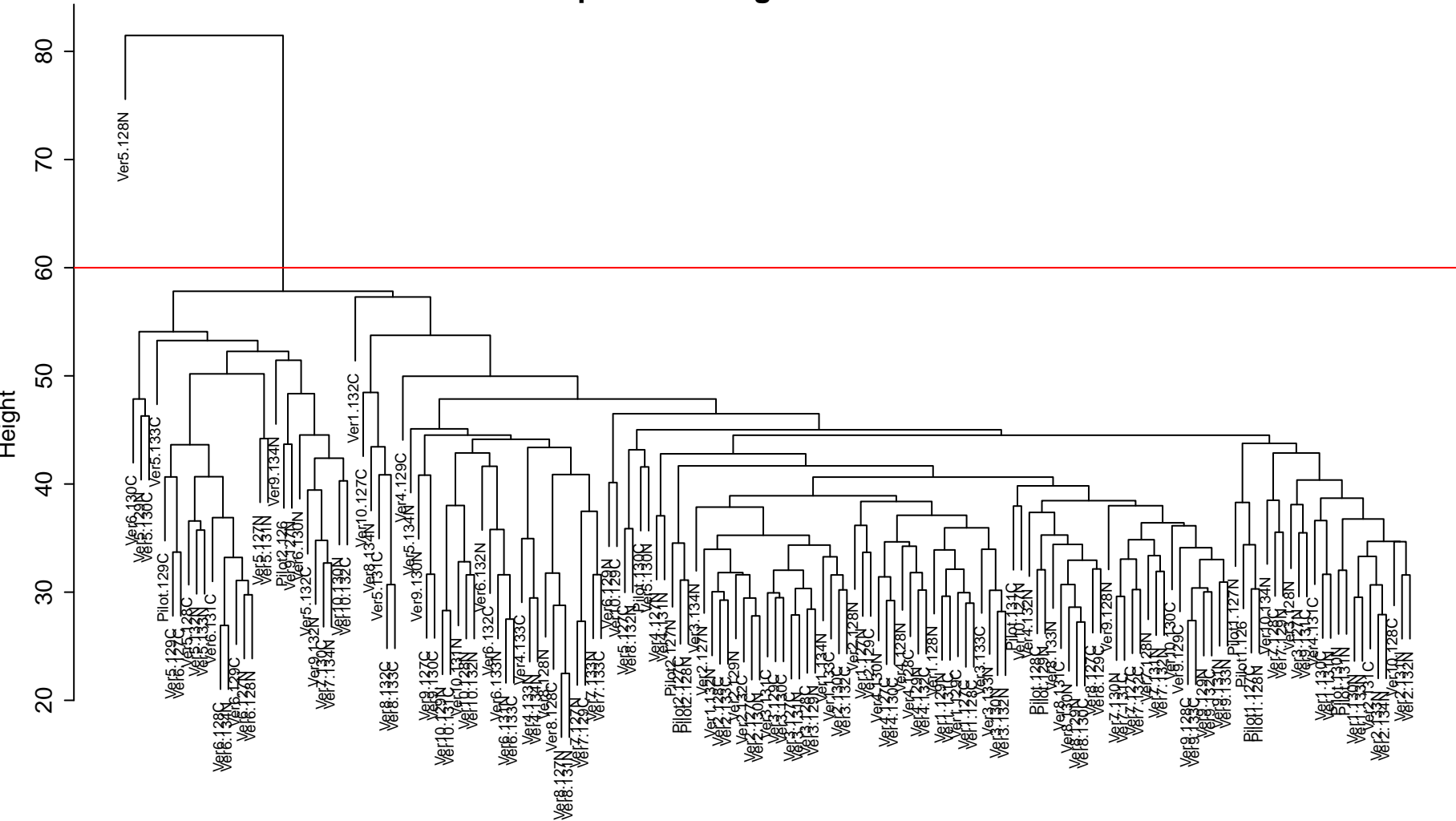

Supplement: Supplementary file 1 — pr2c00563_si_001.zip [file pr2c00563_si_001.zip › S9_WGCNA sample clustering to detect outliers.pdf]

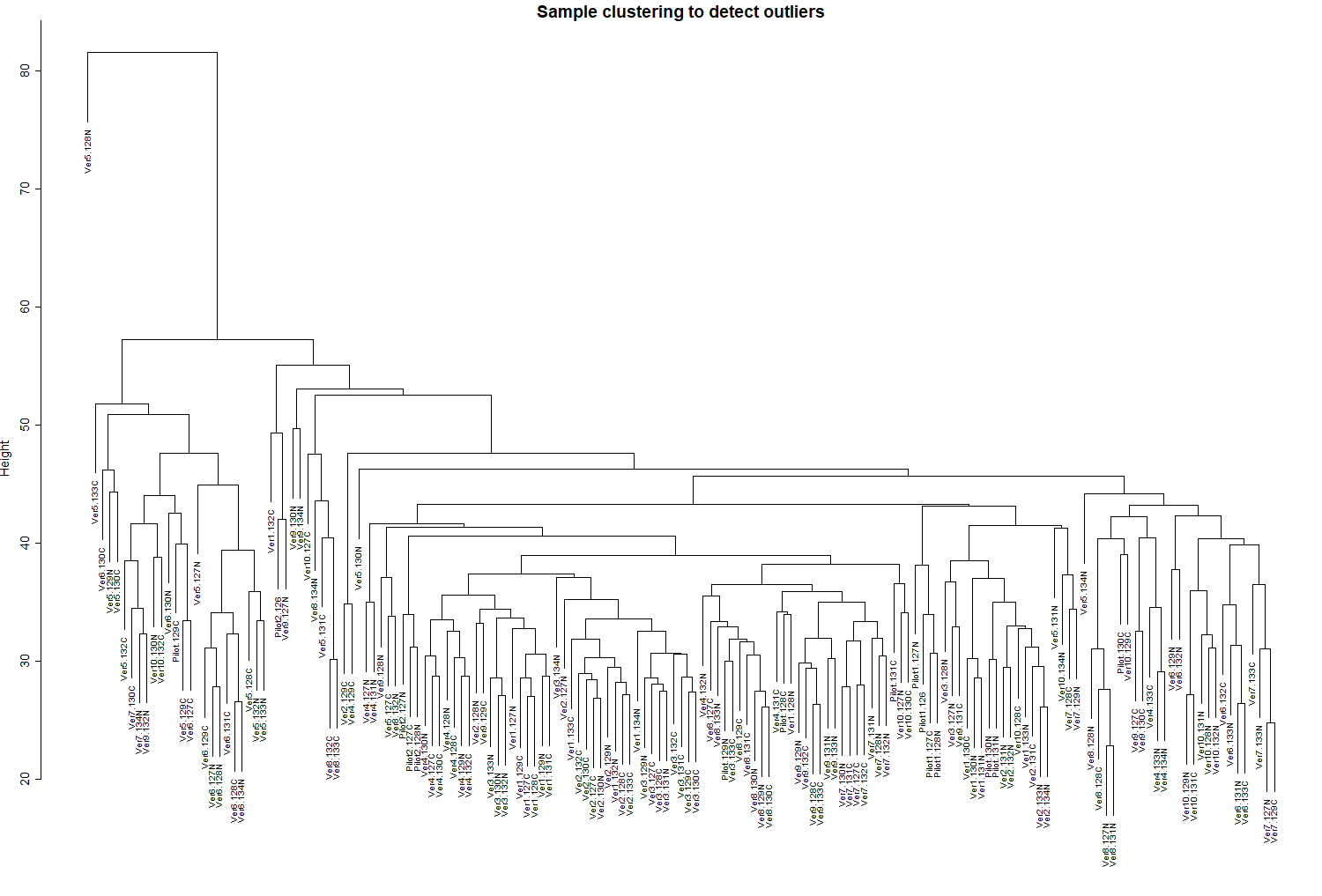

Supplement: Supplementary file 1 — pr2c00563_si_001.zip [file pr2c00563_si_001.zip › S9_WGCNA sample clustering to detect outliers.tif]
